# A new smart healthcare framework for real-time heart disease detection based on deep and machine learning

**DOI:** 10.7717/peerj-cs.646

**Published:** 2021-07-28

**Authors:** Haitham Elwahsh, Engy El-shafeiy, Saad Alanazi, Medhat A. Tawfeek

**Affiliations:** 1 Computer Science Department, Faculty of Computers and Information,, Kafrelsheikh University, Kafrelsheikh, Egypt; 2 Department of Computer Science, Faculty of Computers and Artificial Intelligence, University of Sadat City, Sadat City, Egypt; 3 Department of Computer Science, College of Computer and Information Sciences, Jouf University, Al Jouf, Saudi Arabia; 4 Department of Computer Science, Faculty of Computers and Information, Egypt, Menoufia University, Menoufia, Egypt

**Keywords:** Deep learning, Machine learning, Neural network, ATmega32Microcontroller, Smart application, Firebase cloud, Optimization, Heart diseases

## Abstract

Cardiovascular diseases (CVDs) are the most critical heart diseases. Accurate analytics for real-time heart disease is significant. This paper sought to develop a smart healthcare framework (SHDML) by using deep and machine learning techniques based on optimization stochastic gradient descent (SGD) to predict the presence of heart disease. The SHDML framework consists of two stage, the first stage of SHDML is able to monitor the heart beat rate condition of a patient. The SHDML framework to monitor patients in real-time has been developed using an ATmega32 Microcontroller to determine heartbeat rate per minute pulse rate sensors. The developed SHDML framework is able to broadcast the acquired sensor data to a Firebase Cloud database every 20 seconds. The smart application is infectious in regard to displaying the sensor data. The second stage of SHDML has been used in medical decision support systems to predict and diagnose heart diseases. Deep or machine learning techniques were ported to the smart application to analyze user data and predict CVDs in real-time. Two different methods of deep and machine learning techniques were checked for their performances. The deep and machine learning techniques were trained and tested using widely used open-access dataset. The proposed SHDML framework had very good performance with an accuracy of 0.99, sensitivity of 0.94, specificity of 0.85, and F1-score of 0.87.

## Introduction

The heart pumps blood to all areas of the body on a regular basis. It beats 100,000 times per minute and circulates approximately 19,000 l of blood through our bodies (Lett, Ali & Whooley, 2008). The blood transports waste and provides oxygen and nutrients to our tissues. Anomalies in natural blood flow are the cause of a variety of heart disorders. Heart-related diseases, also known as coronary artery disease, are a category of diseases that affect the heart. Statistics from the World Health Organization indicate that cardiovascular diseases are the leading cause of death in the world. Heart attacks take place when something blocks the bloodflow to the heart so that it does not receive enough oxygen. There are some common symptoms of heart attack as follows:

- Rapid or irregular heartbeats
- Pain in other areas of the body, including the arms and left shoulder
- Shortness of breathing.

Coronary artery disease or coronary heart disease (CHD) develops when the major blood vessels that supply your heart become damaged or diseased. The coronary arteries are the blood vessels that supply oxygen and blood to the heart. Often CHD can cause heart attack. It is the most common form of heart disease in the US, where it accounts for over 370,000 deaths per year (Hage et al., 2009). Wireless continuous health monitoring has been a significant application of newly emerging health care technology in the last decade. Generally speaking, the idea of a continuous health monitoring system and smart medical devices has immense potential for businesses and people’s well-being. This technology provides a real-time device that can provide people with early warnings about their health status. Before clarifying the main points of this paper, it would be better to discuss some physiological sensors that can be used to build continuous health monitoring systems. These sensors can be used to track people in real-time. Additionally, they can be used for both diagnostic and monitoring purposes. Continuous remote health monitoring employs a variety of wireless protocols and networks, including Bluetooth, Wi-Fi, Zigbee, and others (LANs, WANs). Physiological sensors, gateway (access connectivity), and cloud are the basic building blocks of continuous health monitoring (storing data). Heart rate shows our heart’s soundness and assists in determining cardiovascular system health. In the clinical setting, heart rate is monitored under controlled conditions such as measurement of blood, measurement of heart sound, and electrocardiogram (ECG). However, it can also be monitored at home. Our heart pounds to pump oxygen-rich blood to our tissues and take produced cell waste away from our tissues. The more our heart works to achieve these things, the more we use our muscles, the quicker our heart will pound to pump more blood (Ornish, 2010). A heart rate monitor is essentially a wearable system that takes a snapshot of heartbeats and measures the beats per minute (bpm) so that heart rhythm can be accurately monitored using the details. There are two types of heart monitoring methods to create-electrical and optical methods. The electrical approach has a 1 percent average error and a $150.00 average cost. The optical method has a 15 percent accuracy rating and an average of $20 cost. The average resting human heart rate for adult males is about 70 bpm and for adult females is around 75 bpm. The heart rate varies greatly from one person to another relying on fitness, age and genetics. There are several methods for measuring heart rate, such as Phonocardiogram (PCG), ECG, blood pressure wave shape and pulse meters, but such methods are clinical and costly (Kaisti et al., 2019). Over the last two decades, machine learning algorithms have become increasingly popular in a variety of fields. In recent years, machine learning algorithms have been used in the field of health care to analyze large amounts of data in order to increase disease understanding. As a result, machine learning algorithms can be useful in uncovering hidden patterns in medical datasets. Deep learning and Machine learning have witnessed tremendous progress. In Masethe & Masethe (2014), an experiment was carried out to predict heart attacks and a comparison was made to find the best method of prediction. This can be a useful tool for doctors to predict critical cases in their practice and provide appropriate advice. The predictive accuracy determined by the J48, REPTREE and SIMPLE CART algorithms suggests that the parameters used are reliable indicators for predicting the presence of heart diseases, but this research has the drawback of not being able to predict heart disease in real time. In Chaurasia & Pal (2013), three classifiers, ID3, CART and DT, are used to construct the model, with CART being the most accurate with 83.49 percent and 0.23 s. The important characteristics of heart diseases are cp (chest pain), slope (the slope of the peak exercise segment), Exang (exercise induced angina) and Restecg (resting electrocardiographic). However, this research has the drawback of not being able to predict heart disease in real time. Sudhakar & Manimekalai (2014) describe the various methods that have been used in recent years to calculate the prediction rate in heart disease. ANN, BN, decision trees and classification algorithms are some of the methods used, but this research has the drawback of not being able to predict heart disease in real time. In the study of heart disease diagnosis using neural networks arbitration by Olaniyi, Oyedotun & Adnan (2015), two methods are used: SVM and ANN. Support vector machine is the best algorithm for diagnosis of heart disease, with an accuracy rate of 87.5 percent and high values of sensitivity and specificity but predict of heart disease is static and no real time. In Otoom et al. (2015), a pulse sensor is used to monitor heart rate and transmit it wirelessly to a mobile device via an Arduino microcontroller. Three classifiers are used to create the model: BayesNet, SVM and FT, with SVM being the most accurate with 88.3% accuracy. Joshi & Nair (2015) confirm that three methods of heart disease prediction such as Decision Trees, Nave Bayes, and K Nearest Neighbour were used to develop the model using classification based data mining techniques, with decision tree being the most accurate with 92.3% accuracy. There has been no real-time forecast in this work. Sultana, Haider & Uddin (2016) assert that five classifiers are used in the analysis of data mining methods for heart disease prediction: KStar, J48, SMO, Bayes Net and multilayer perceptron, with SMO showing the highest performance of 89 percent accuracy. In this work, no real-time projection has been made. For patients with heart problems, sensors are used to provide continuous monitoring. For instance, consider the work of Ali (2017). Using a heartbeat sensor and a thresholding algorithm for abnormality detection, an Arduino-based heart rate monitoring and heart attack detection system was proposed. Due to the non-ideal nature of the components, the system has the limitation of not providing accurate monitoring system results. A state-of-the-art survey on physiological parameters and activity monitoring systems developed in a wearable platform is presented in Majumder, Mondal & Deen (2017). Deep learning methods have gained significant interest from medical researchers. A recent survey indicates that about half of the healthcare organizations are planning to use deep learning (Kamilaris & Prenafeta-Boldú, 2018). Deep learning algorithms offer a great opportunity for the early detection of heart diseases. The use of deep neural networks in dataset analysis thus enables a significantly faster and more reliable diagnosis of heart diseases than with conventional methods. The methods called deep learning learn to represent objects from a low level (a raw input) to a high and more abstract one (Makantasis et al., 2015). This is built up by several layers that represent the different levels of representation; hence the name deep learning (Gulli & Pal, 2017). Deep learning is defined as an automatic algorithm structured or hierarchical structure that emulates human learning in order to obtain certain knowledge. It stands out because it does not require previously programmed rules, but the system itself is capable of learning by itself to carry out a task through a pre-training phase. It is also characterized by being composed of intertwined artificial neural networks for information processing. It is mainly used for predictive analysis automation. The algorithms that make up a deep learning system are found in different neuronal layers composed of weights (numbers). The system is mainly divided into three layers:

1. Input layer: It is made up of the neurons that assimilate the input data, such as an image or a database.
2. Layer: It is the network that performs the information processing on and do intermediate calculations. Every more neurons in this layer there are, more complex are the calculations performed.
3. Output Layer: It is the last link in the chain, and it is the network that makes the decision or makes a conclusion by providing output data (Najafabadi et al., 2015).

**Figure 1:**
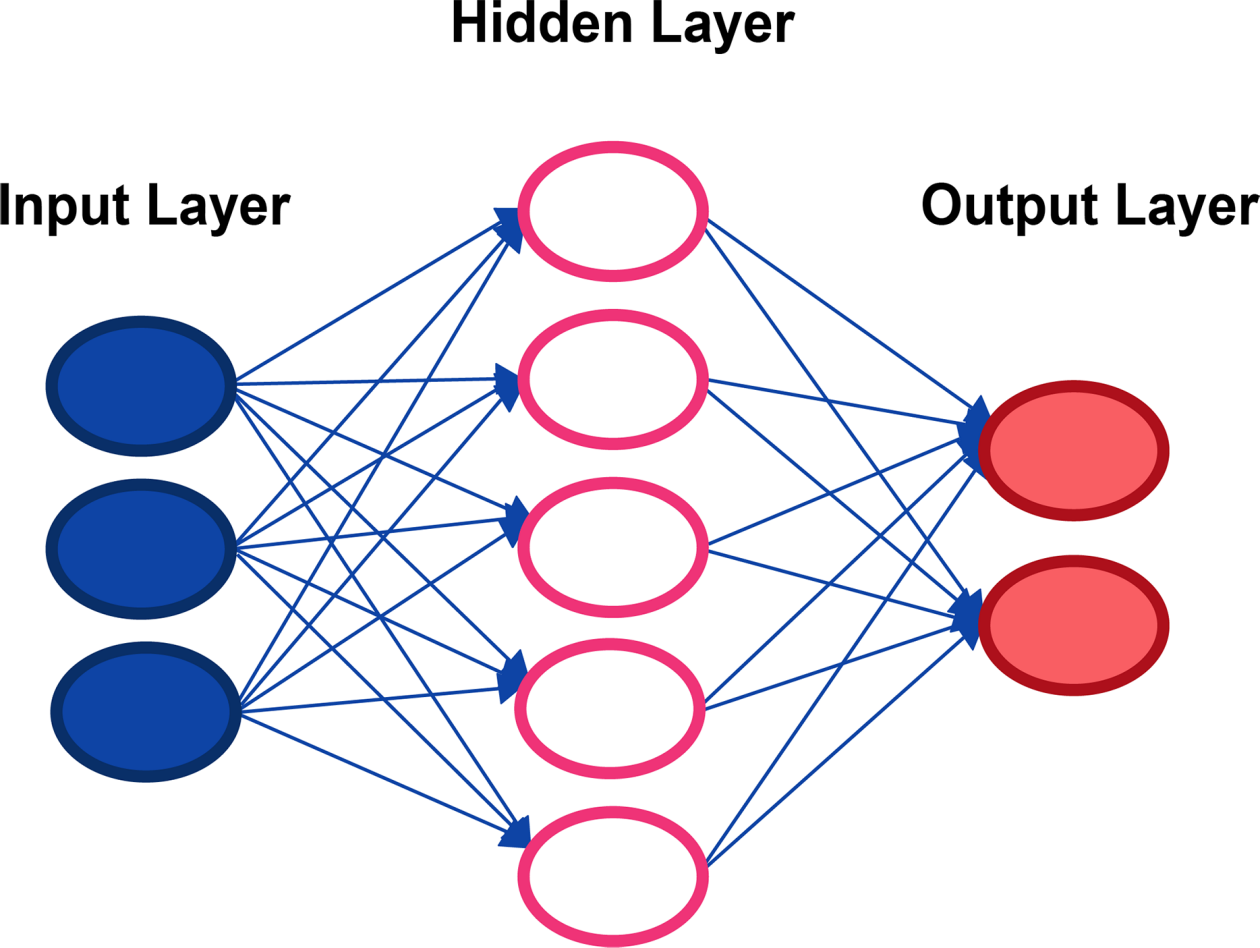
Representation of a classical neural network.

How deep learning works computer programs that use deep learning go through the same process as the young child who learns to identify an object? Each algorithm in the hierarchy applies a nonlinear transformation to its input and uses the learn rate to create a statistical model as an output. The iterations continue until the output has reached an acceptable level of precision. It is called deep learning because all the data must pass through all hidden layers for processing step. The typical and essential example of a deep learning type method is multi-layer perceptrons, which is basically a mathematical function that maps an input to an output; This is a composition (or a network) of multiple simpler non-linear functions (Karlik & Olgac, 2011) called neurons or perceptrons (like the one in Fig. 1), where the weights associated with each one in the composition are trained (typically by back propagation) to learn the function that relates the inputs to the outputs of a supervised learning problem.

**Figure 2:**
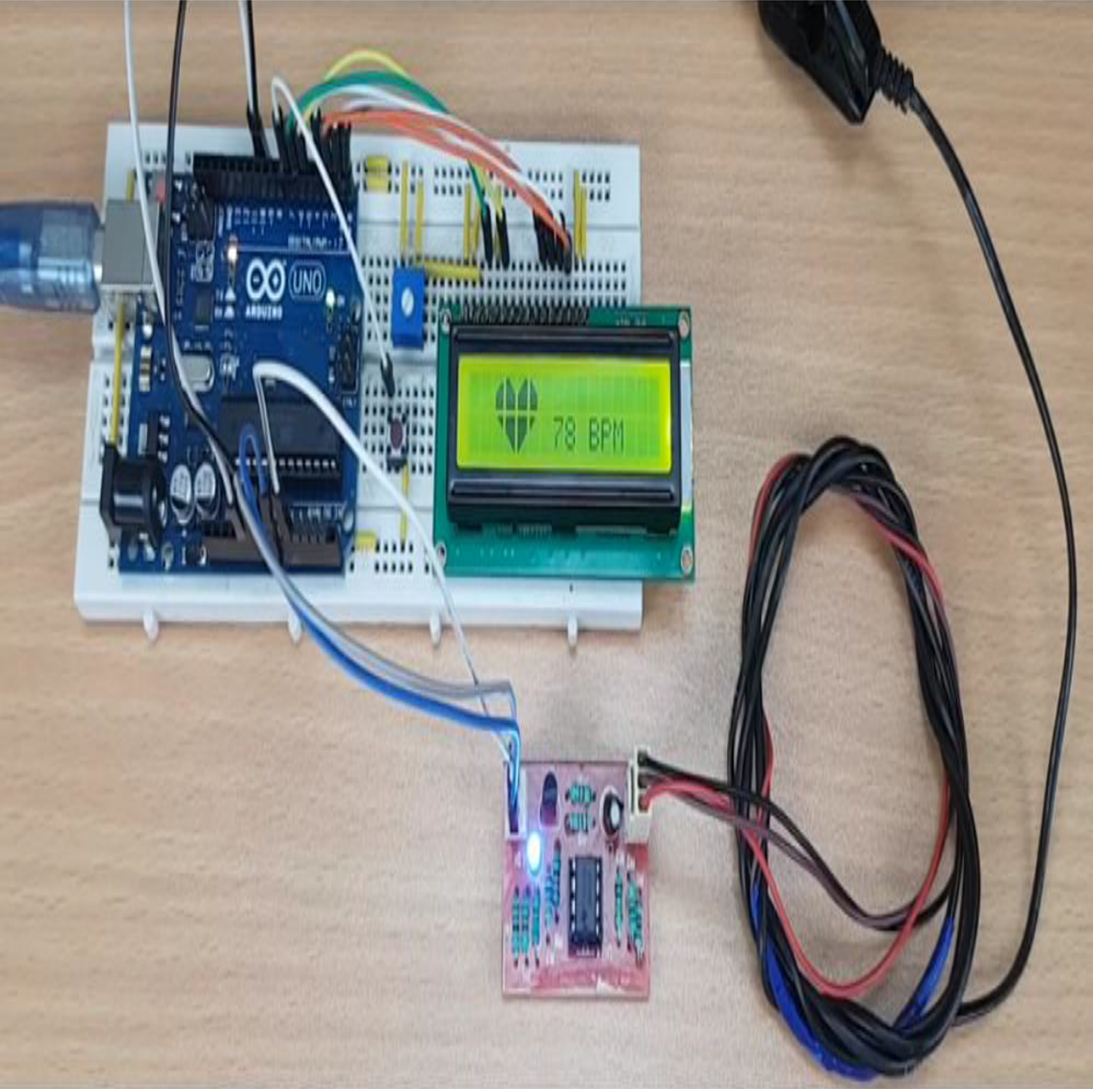
Microcontrollers used in vehicle engine control systems, medical equipment, and remote controls.

Several problems arise, once the classical approach such as the multi-layer perceptrons is used. For instance, these models have a very high number of weights, therefore the generalization error that can occur if the data are not balanced is potentially high. Furthermore, there is no invariance with respect to the type of distortion in the input data (Olaniyi, Oyedotun & Adnan, 2015). In particular, there are three architectures that make use of deep learning generators (CNN networks, and that have evolved from the same origin: RCNN (Yu et al., 2019), Fast R-CNN (Girshick, 2015) and Faster R-CNN (Jiang & Learned-Miller, 2017)). As their names suggest, each one is implemented with the goal of being faster than the previous one and each one has stood out in the first places with their performance in well-known databases in the period when they were published. In Fig. 2, the scheme of the R-CNN system is broadly shown. From an input data are generated by photoplethysmogram sensor (PPG), which sequentially enter a network convolutional that extracts characteristics of each one and classifies them as an object with a certain probability. Our contribution of this paper is to design and develop a one-of-a-kind system that can both monitor and predict disease at an early stage. The following are the research challenges hindered in designing the proposed system:

- Collecting, storing, and displaying real-time sensor data
- Optional features using deep learning and machine learning algorithms to predict heart disease in real time.

Our goal is to develop a system that addresses those research challenges. If we achieve our objectives, patients will be less likely to visit hospitals on a regular basis. This is especially vital for the elderly, who require special health care and monitoring around the clock. Another source of concern is the number of deaths caused by CVD that go unnoticed. We can create a system which predicts heart disease beforehand. To do so, we must investigate the risk factors for heart disease, such as heart rate, age, cholesterol, chest pain, blood sugar, etc. We can design a system capable of preventing health-related disasters using physiological sensors and a microcontroller for a continuous health monitoring system. As a result, we propose a smartphone-based heart disease prediction system that can perform both monitoring and prediction. The proposed research is applicable not only to the elderly, but also to infants, adults, and stroke patients in terms of predicting heart disease. There are three challenges to be observed. First, the data must be preprocessed. This means that the input signal must be treated to eliminate everything that does not contribute information to the model. In addition, it will be necessary to ensure that the distribution of the set data is uniform. To get this done, we have generated a new data that allowed training the model with a collection of data with balanced classes. It is proposed to use two different methods to test the dataset. The following is the problem of automatic identification of diseases from the dataset. This will be the most complex problem to deal with, since a system capable of predicting the kind of diagnosis must be designed and developed from ECG signals also different architectures based on the combination of convolutional neural networks, Recurrent neural networks and classical neural networks. The objective is to obtain a model of great precision in diagnosis. Finally, it is intended to develop an application that allows patients to send the data corresponding to the recording of an electrocardiogram. The result of this request will be the display of the information contained in the registry and a diagnostic proposal, which will include the probability estimated that one of the diseases detected automatically is suffered by the model based on deep learning. This could potentially result, from this work or future investigations as a result of it, in a tool of great value for the diagnosis of patients. Consequently, it is a useful contribution to our community. Therefore, it is among the objectives to achieve an extrapolated system to other scenarios beyond the training dataset. Many factors contributed to our motivation in the area of health and the use of physiological sensors to improve health care. Saving lives, as the main concern of all of us, is the first and last factor that guided us towards this area. Every day, many heartbreaks and sorrows occur when family members succumb to heart disease; there is beyond anyone’s ignorance of this disease. Thus, this paper aims to the following:

- Conduct a review of the literature on physiological wearable sensors, machine learning algorithms, interfacing devices, and software.
- Conducting a review of the literature on physiological sensors linked to heart disease and selecting appropriate sensors.
- Assess machine learning algorithms and choose one model that performs best in predicting heart disease.
- Create an android application to visualize and display the prediction results.

This paper is organized as follows: “Methodology” introduces the methodology used in SHDML framework. “The Smart Healthcare Framework SHDML” describes our proposal SHDML framework used throughout this paper, “Experimental Result and Comparison” describes the SHDML experiences and results. Finally, “Discussion and Conclusion” presents our conclusions.

## Methodology

There are many of methodologies used in SHDML framework such as:

- Wearable embedded system
- Android
- Desktop
- ML
- DL

### Wearable embedded system

**Figure 3:**
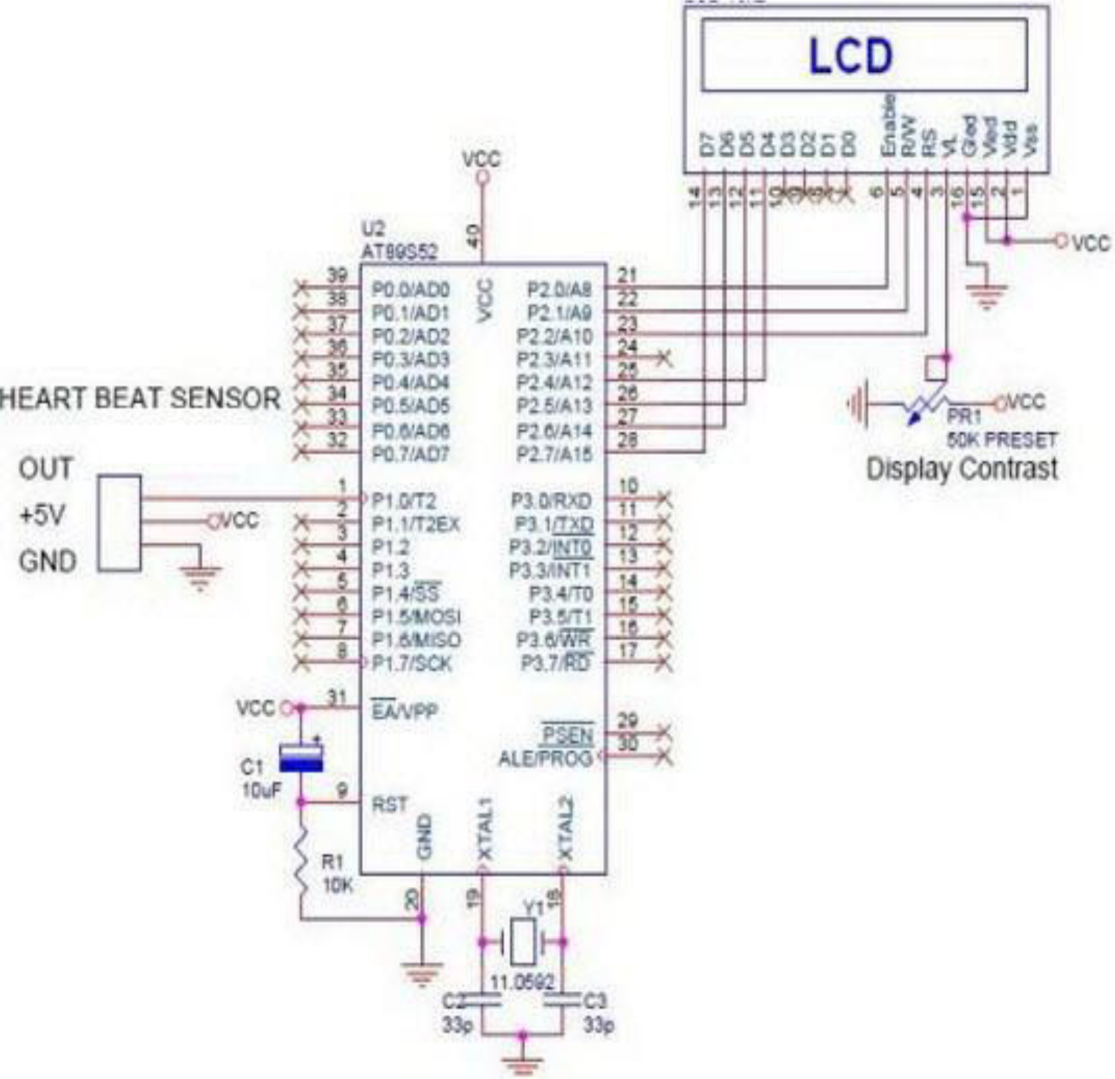
ADC analog to digital converter interfaces and timer with microcontrollers.

**Figure 4:**
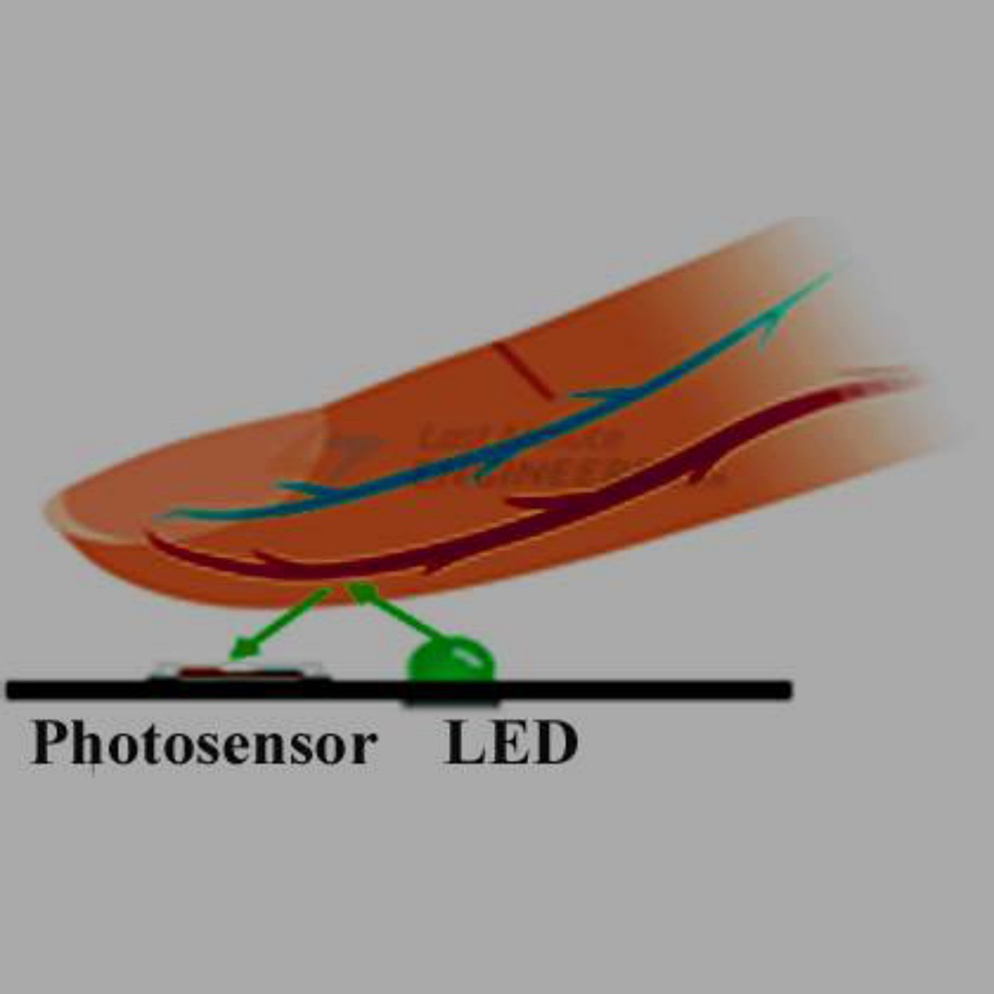
The light sensor.

**Figure 5:**
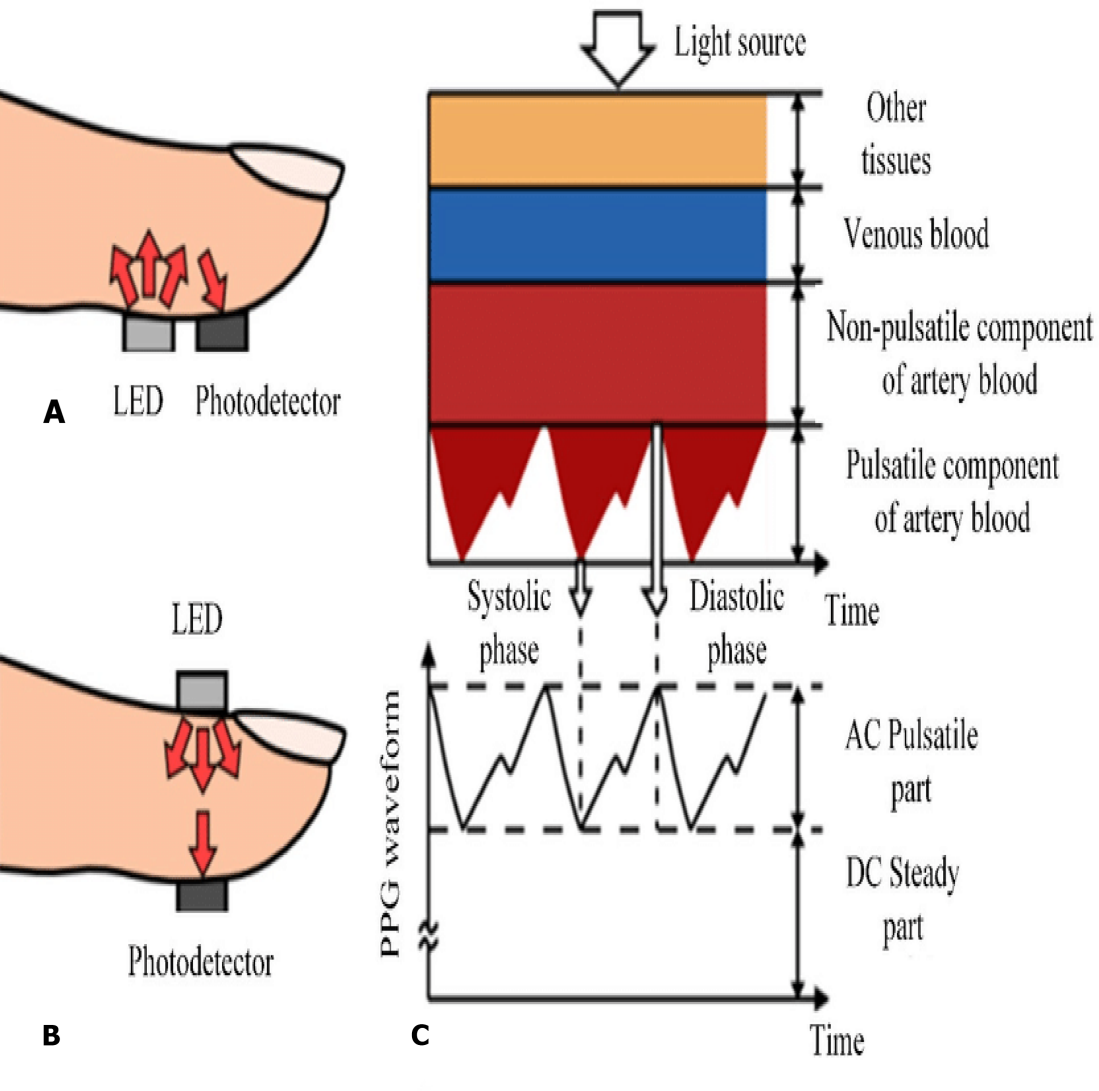
5-s representation of an electrocardiogram of a patient from the training data.

In the recent years, smart watches have commenced to be released by major electronics companies as well as by new start-ups. One of the first offerings was the Samsung Galaxy Gear in September 2013. About a year later, the Apple Watch was relased in April 2015. Most proposed frameworks for health monitoring leverage a three architecture: A Wireless Body Area Network (WBAN) consisting of wearable sensors as the data acquisition unit, communication and networking and the service layer. We have proposed an embedded system that contain Microcontroller linked with pulse sensor that measures heart rate of human. Microcontroller transmits gathered medical data to an Android App through Wireless Bluetooth communication. The Android app store heart rate in cloud server, and user can access measured heart rate; step counts thorough android app. User confirms whether he suffers from coronary heart disease or not using deep learning module through android/desktop app. The built-in feature is used to build a wearable computer. The embedded system is a ‘computer system’ that consists of a combination of micro processing, memory and input/output Peripheral devices with a specific purpose, with modern embedded systems based mainly on an integrated microcontroller as shown in Fig. 2. Wearable system is implemented on the basis of atmega32 microcontroller with pulse sensor and other modules, pulse sensor based on PPG technology, PPG (photoplethysmography) sensors use light-based technology to measure the blood flow rate as controlled by the heart’s pumping action. We have added the Bluetooth module for interacting with the Android app for sending BPM beats per minute and the LCD monitor for viewing BPM on a wearable devices. The proposed framework is implemented by developing a “Pulse Sensor Library” which handles ADC analog to digital converter interfaces and TIMER with microcontrollers, as shown in Fig. 3. The LED emits light which will fall on the vein directly. The veins will have more blood flow inside them only when the heart is pumping (systolic), the veins will have little blood flow inside them during (diastolic) so if we monitor the flow of blood, we can monitor the heart beats as well. The light sensor as shown in Fig. 4, will pick up more lights since they will be reflected by the blood flow. Threshold Value ‘T’, which is defined to be the start point for each beat (25% or 50%) of signal, the analog and digital converter (ADC) is the start to read an analog signal at T point which represent the received light of blood flow. Signal is converted into digital value as shown in Appendix 1 and applying noise cancellation as shown in Fig. 5 (dicrotic notch which represents closure of the aortic valve). At each 2 ms ADC halt the microprocessor for reading from sensor, this operation was achieved using an INTERRUPT mechanism. Calculate average for 10 or more beats to determine our heart beats in minutes.

### Smart android app

The android app was developed with the JAVA programming language. Our android app represents friendly main UI which include many features as daily heart rate measurement, step counts, SMS emergency for heart attack, coronary heart disease prediction, Medical analyses history and Nearest hospitals maps. The user has to create an account to grant an access to the android app features. The info that the user has to import is a name, an email, a phone number, a password, a birthdate, an emergency phone number and a profile picture. The daily steps count, heart rate check and CHD prediction are uploaded to a Firebase Cloud server and the user can retrieve their history data. An emergency ASA is sent to the emergency number when the BPM is at critical state. We used a lot of APIs in our app, but will focus on and discuss about the most important used APIs. Important used APIs

- Bluetooth: Android Bluetooth APIs allow applications to link wirelessly to other Bluetooth devices, allowing point-to-point and wireless multipoint functions. The Android application may use the Bluetooth APIs to scan for other Bluetooth devices request the local Bluetooth adapter for paired Bluetooth devices Transfer data to and from another device, to communicate with the wearable device.
- SMS Manager: handles SMS processes, such as sending SMS messages, email, and PDU (Protocol Data Unit) SMS messages. Used for sending emergency SMS to the emergency phone number that the user registered when the heart rate is in critical case.
- Maps API: Your app can request the last known location of the user’s device using the Google Play location services APIs. You are mainly interested in the actual position of the consumer, which is typically equal to the last known location of the device. Used for locate the nearest health care centers.
- PyTorch: PyTorch Android API Running ML on edge devices are significantly increasing as applications that continue to require lower latency. This is also a fundamental item for the defense of privacy strategies, such as federated learning. As of PyTorch 1.3, PyTorch supports the end-to-end workflow from Python to iOS and Android deployment. This is an early, experimental release that we will expand on in a variety of areas in the coming months: to include APIs that cover specific pre-processing and integration tasks required to integrate ML into mobile applications Further performance enhancements and coverage for mobile CPUs and GPUs org.pytorch: PyTorch android are the key dependency of the PyTorch Android API. Used for sending users health data to the local Pytorch model that predicts whether the user will be a CHD patient in the next 10 years or not to provide a predictive response to the android UI app.

### Machine learning model

In the section of machine learning, the inputs (features) are obtained from the UI, doing some operations on it then, predicting the output. So far, this can be done by simple traditional programming. However, but we are dealing with a massive number of combinations among our dataset, so we have to use machine learning in which the machine will find out the best numerical function for predicting the result out of the inputs. Machine learning is an application of artificial intelligence (AI) that provides systems with the ability to automatically learn and improve from experience without being explicitly programmed. Machine learning focuses on the development of computer programs that can access data and use it to learn for themselves.

#### Support vector machine (SVM)

**Figure 6:**
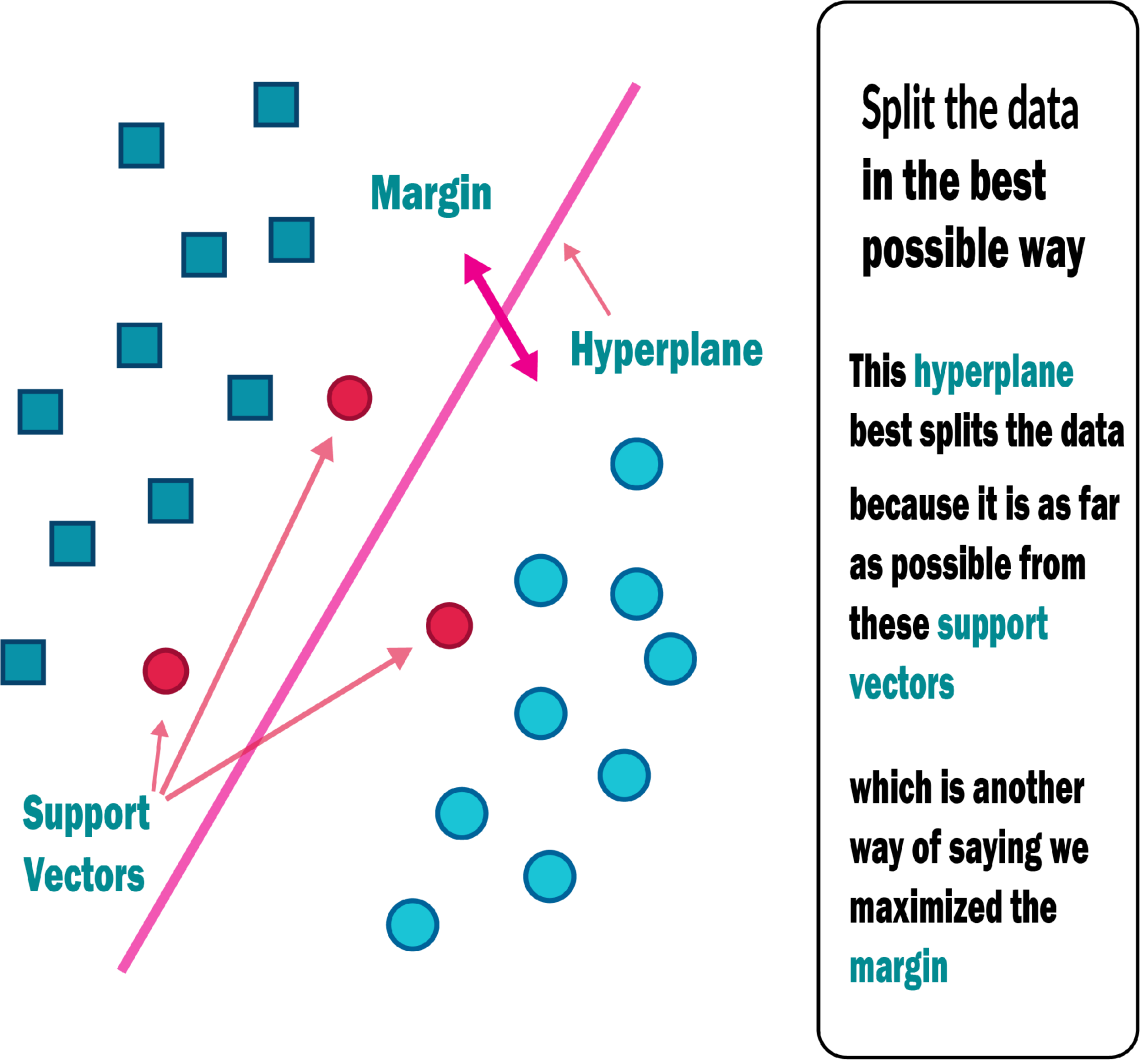
Support vector machine.

An SVM classifies data by locating the hyperplane that maximises the difference between two classes. The support vectors are the vectors that define the hyperplane shown in Fig. 6. The use of the SVM for data set classification has its own set of benefits and drawbacks. By observing properties, a medical data set can be non-linear and have a high dimensionality. SVM is undoubtedly one of the most common classification algorithms.

- To begin, regularisation parameters are used to avoid the problem of overfitting, which is one of the most difficult problems in decision trees.
- A kernel tree is used to prevent expert knowledge by using kernel knowledge. All of the features listed above may be useful for medical diagnose datasets, resulting in a more effective prediction framework for physicians (Nashif et al., 2018).

#### Naive Bayes

The Naive Bayes algorithm is an efficient predictive modelling algorithm. It’s a statistical classifier that makes no assumptions about attribute dependencies and tries to maximise the posterior likelihood in deciding the class. Based on the above formula, a Bayesian classifier determines the conditional probability of an instance belonging to each class, and then classifies the instance into the class with the highest conditional probability. The probabilistic model can then be used to make predictions with new data using the Nave Bayes Theorem if these probabilities are determined (Nashif et al., 2018).

(1) }{}P(A/B) = \displaystyle{{P(B/A)P(A)} \over {p(B)}}

where, P(A) = prior Probability of class, P(B) = prior Probability of predictor, P(B/A) = Likelihood, P(A/B) = Posterior Probability.

#### Random forest and decision tree

Random Forest is a well-known and highly efficient machine learning algorithm. Bagging, also known as Bootstrap Aggregation, is a type of machine learning algorithm. The bootstrap is a very effective statistical method for estimating a value from a data set, such as mean. Many samples of data are taken, the mean is estimated, and then all of the mean values are combined to get a better estimate of the true mean value. The same approach is used in bagging, but decision trees are commonly used instead of estimating the mean of each data set. Several samples of the training data are taken into account, and models are produced for each sample. Although a forecast for any data is needed, each model provides one, which is then averaged to obtain a more accurate estimate of the actual output value (Nashif et al., 2018).

#### Simple logistic regression

Logistic regression is a machine learning methodology that originates in the field of statistics. This approach can be used for binary classification, where two classes are used to classify values. The aim of logistic regression, like linear regression, is to measure the values of the coefficients within each input variable. In contrast to linear regression, the output prediction is made using a non-linear function known as a logistic function. Any value between 0 and 1 is transformed by the logistic function. The likelihood of a data instance falling into either class 0 or class 1 is calculated using logistic regression predictions. This could be required in circumstances where more justification for a prediction is required. When attributes are unrelated to the output variable and attributes that are associated to one another are omitted, logistic regression performs better (Nashif et al., 2018).

### Deep learning model

Deep Learning that subfield of machine learning is important in our project for helping embedded system to think and the android application to anticipate the user will have a heart attack in the next ten years. We used specifically ‘Pytorch’ library because it’s an easy library to learn and build a model as well as it’s combatable with android applications in connecting so it’s the best choice from deep learning libraries. We used ‘CNN’ to help us in improving our performance from the relations and features extraction that increases the features of our data by finding new relations between them all of that get the best pattern and combination of our data to predict the results

#### Artificial neural networks (ANN)

Biologically inspired artificial neural networks, also known as MultiLayer Perceptrons (MLP), are capable of modelling highly complex non-linear functions. One of the most important methods of machine learning is artificial neural networks (ANNs). They are brain-oriented systems that are designed to mimic how humans think, as the word “neural” implies. The input, output, and hidden layers of neural networks are made up of three layers. A hidden layer is generally made up of units that convert the input into a pattern that the output layer can control. ANNs are excellent tools for extracting and teaching a computer to identify patterns that are too complicated or vague for a human programmer to recognise. Since the 1940s, neural networks have been in use, and in recent decades, they have become an important part of artificial intelligence due to the arrival of a new technique known as “back propagation”, which enables networks to know how to change their hidden layers of neurons in cases where the outcomes do not meet the creator’s expectations (Nashif et al., 2018).

#### Recurrent neural networks

The outputs from previous states are fed as input to the current state in recurrent neural networks (RNN). RNN’s hidden layers have the ability to remember details. The output generated in the previous state is used to update the hidden state. RNN can be used to forecast time series because it has Long-Short Term Memory (Ali et al., 2021), which allows it to recall previous inputs.

## The Smart Healthcare Framework SHDML

**Figure 7:**
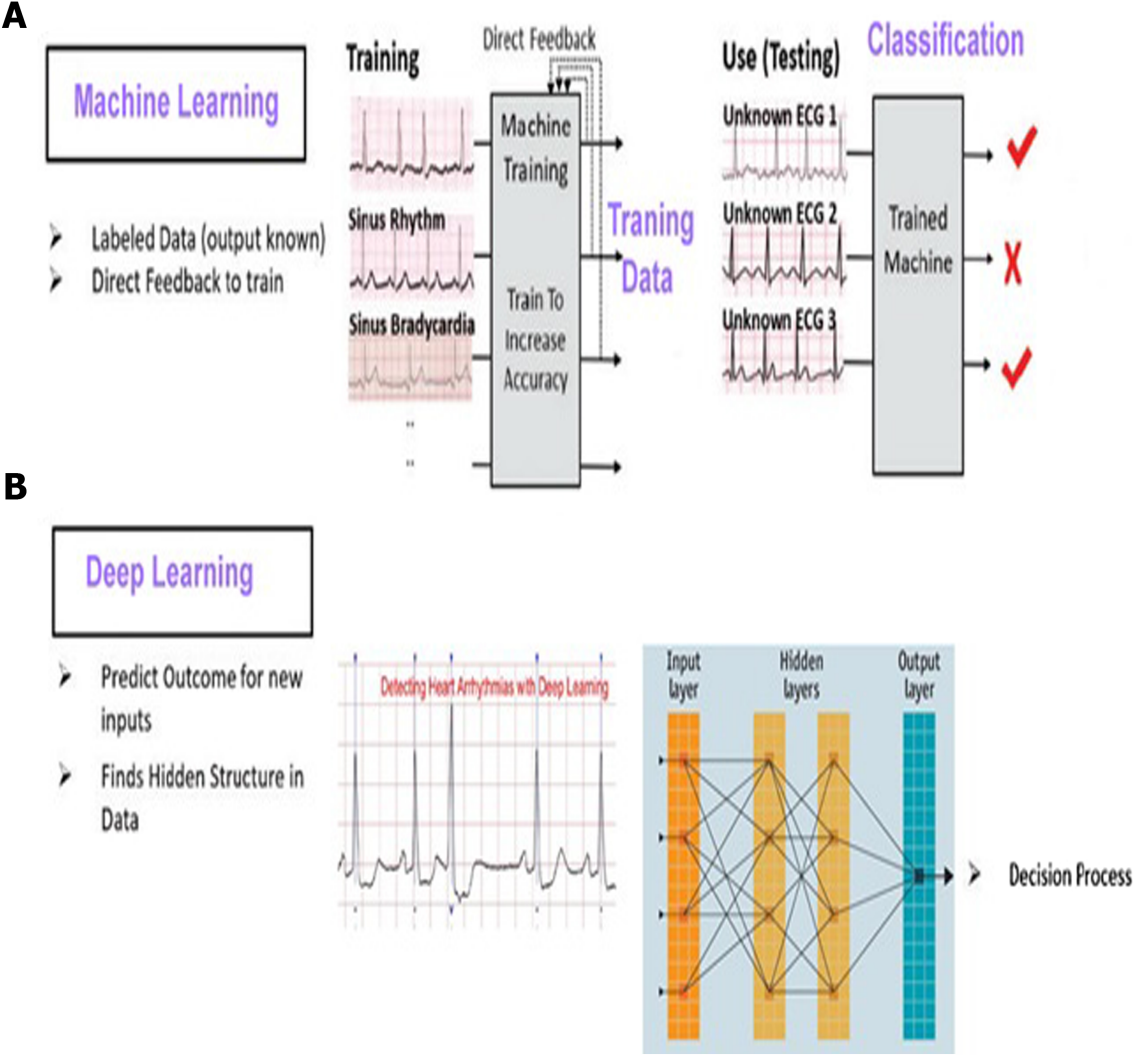
SHDML framework consist of ML and DL.

The SHDML framework consists of two stages. The first stage SHDML is able to monitor the heart beat rate condition of a patient. The heart beat rate is detected using photoplethysmogram sensor (PPG). The signal is processed using ATmega32 Microcontroller to determine heart beat rate per minute. Then, it sends the heart rate represented as BPM to Android App Via Bluetooth communication. Then Android app sends SMS alert to the mobile phone of medical experts, patient’s family member, or their relatives via SMS containing user’s current location. After that, Android app counts daily steps. Android/Desktop app allow user to check nearest hospitals, cardiac centers, Health centers and also user’s current location. Android/Desktop app let the recognize whether he suffers from heart disease (Heart Disease) or not. The second stage, SHDML has been used in medical decision support systems to predict and diagnose heart diseases. By training the Deep and machine learning technique that analyze user’s data to detect heart disease as shown in Fig. 7. As our data—on which the machine will train—includes the desired output, so the used DL and ML type is supervised machine learning,

**The First Stage**

**Figure 8:**
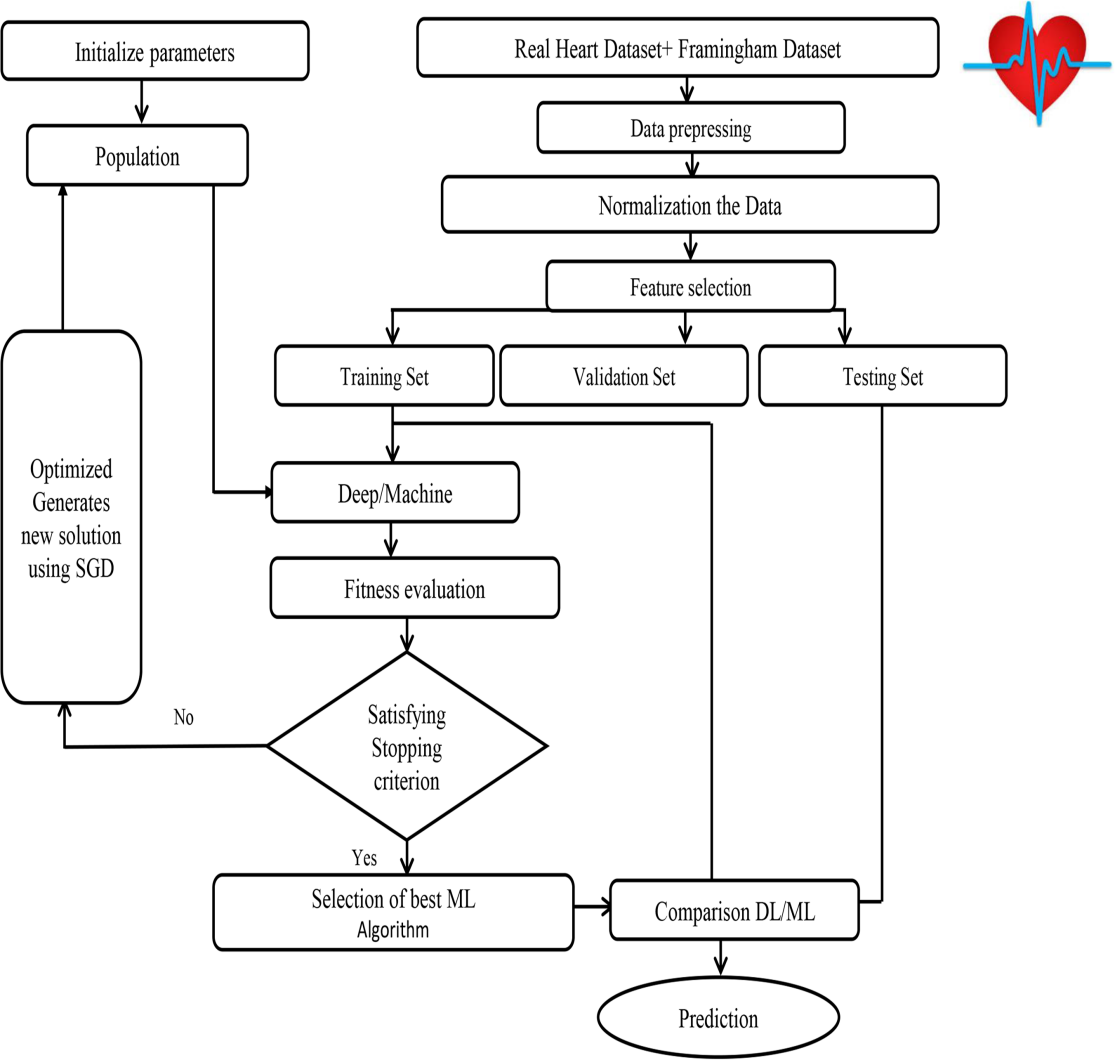
SHDML framework flowchart.

**Figure 9:**
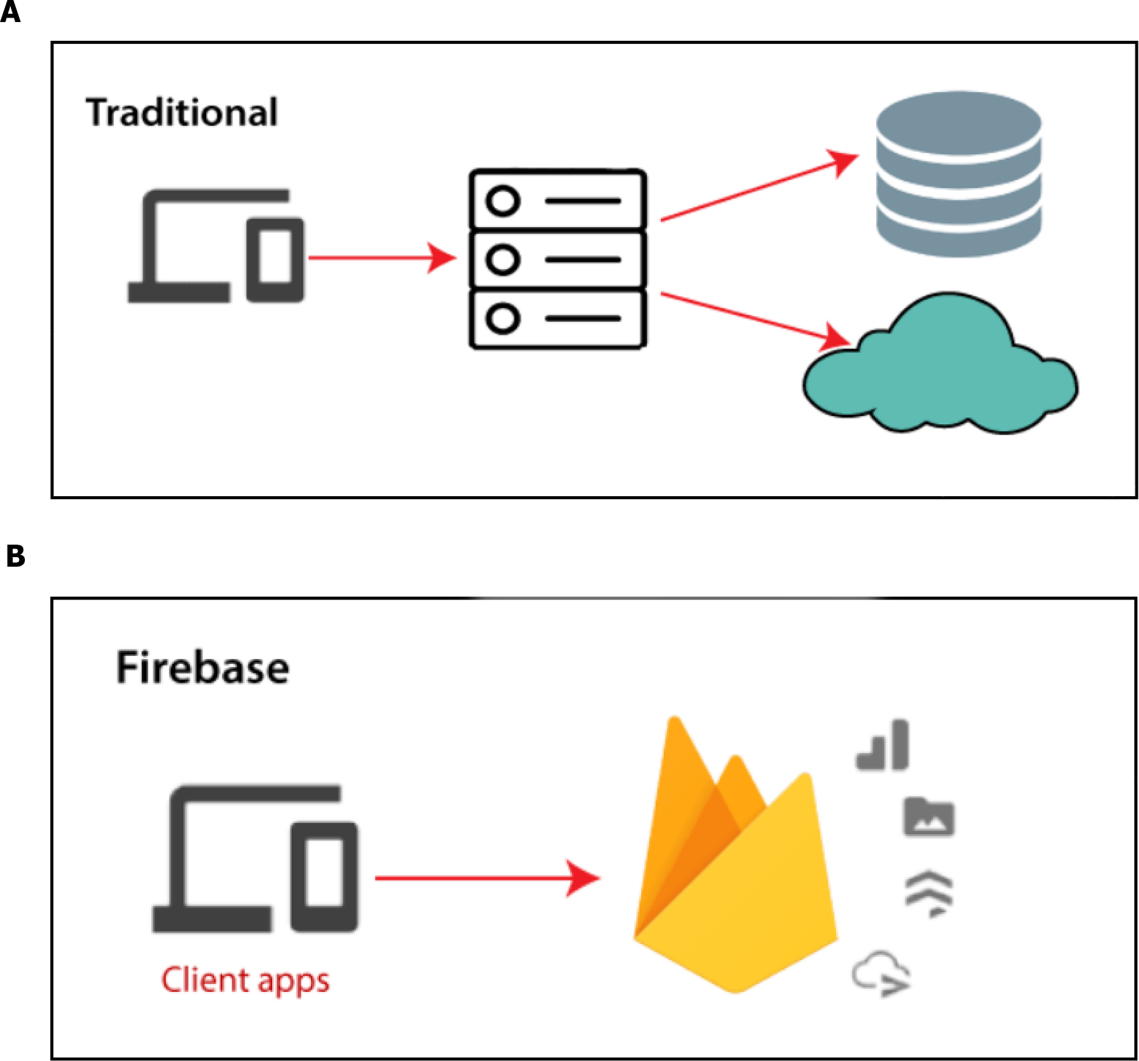
Firebase real-time database.

Input data: We are collecting our data on which the model will train and it has to be accurate, trusted and balanced, so we got it from Framingham University (D’agostino et al., 2008) which is specialized on the research of heart diseases in addition to the data obtained from real sensor data as shown in Fig. 8. We use a Firebase Cloud real-time database to collect and store authentic data unlike a traditional relational database in Fig. 9A. Firebase: is a backend-as-a-service (BaaS) real-time database that allows a list of objects to be stored in a tree format. We can synchronize data between devices as shown in Fig. 9B.

Authentication:

- Organizing and managing users.
- We support email and password login only.

Real time database: The Firebase real-time database uses data synchronization instead of using HTTP requests. Any user can receive the updates within milliseconds like user’s details and history of health and prediction. We have three tables:

1. User table that contains needed info.

- This table includes nodes, each node is the ID of a user.
- When a new account is created a new node is generated with randomly auto generated string ID.
- The string ID node has some attributes (name, phone, profile-image, security-level, user-id, age, emergency-num)

2. Prediction history table that contains CHD prediction results.

- Includes string ID nodes as user table.
- In this table each node has attributes that are auto generated with current date.
- The attribute contains the prediction info.

3. Health history table that are contain daily steps count and BPM.

- Includes string ID nodes as user table.
- In this table each node has attributes that are auto generated with current date.
- The attribute contains the history info like BPM and steps count.

**Figure 10:**
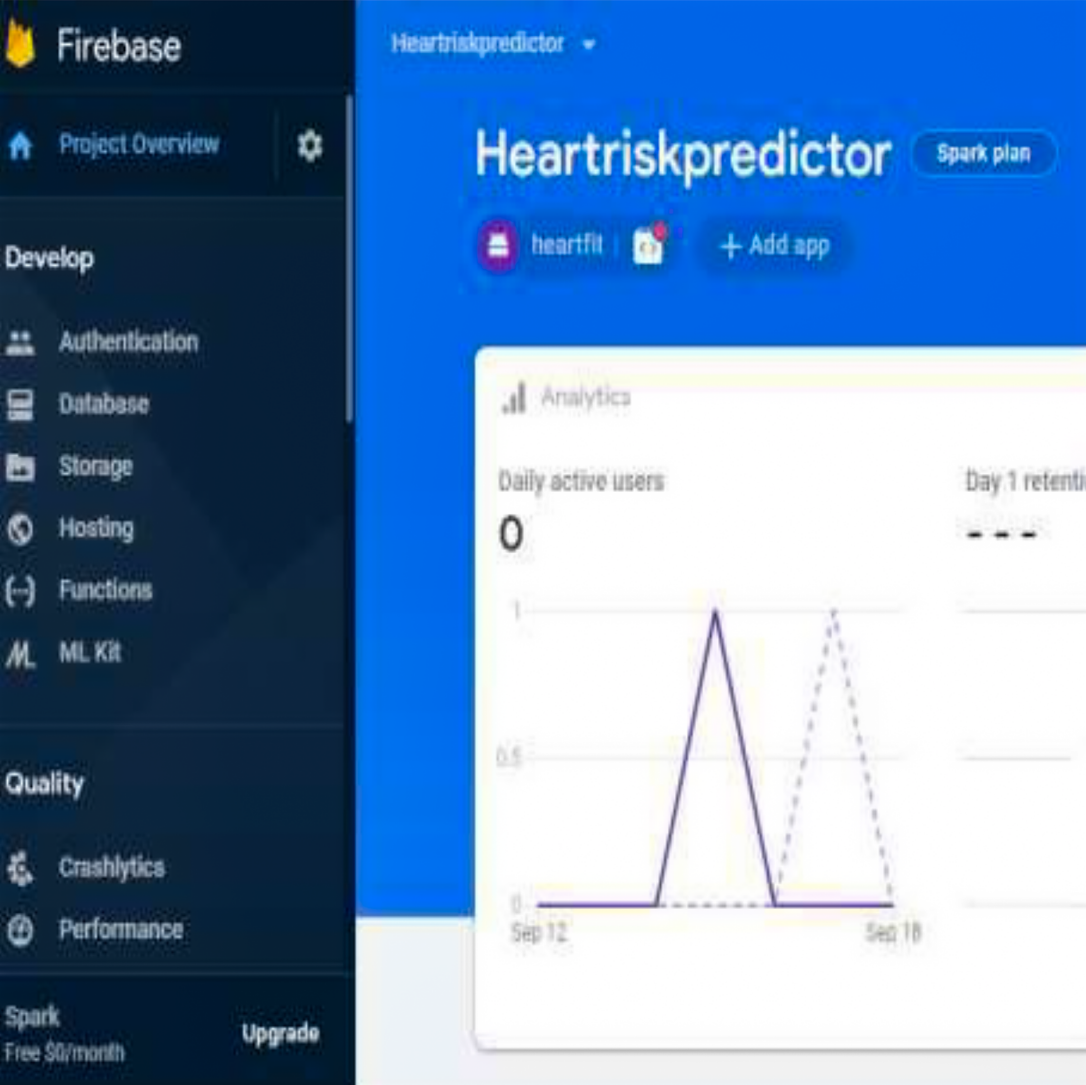
A folder generated in Firebase.

Cloud Storage:

- Easy to use through android SDK.
- Enable users to stores files or media data like profile. pictures. A folder is generated named with the string ID of the user; this folder contains the profile image as shown in Fig. 10.

To do data preprocessing for making the data readable and understandable for the machine module just like cleaning the data for making it null-free. The last phase is to do some data transformation like normalizing or scaling (a simple function for overcoming skewed data). The fact of using models with different validation and training distributions, could compromise the generalizability of this model and produce overfitting. This is a matter to take into account in future evaluations. In addition to when validating the data the ideal would have been having more than one distribution and with a greater amount of data. In this way, it would have been possible to reserve data for a final evaluation, after the construction of the joint model.

Data Preparation Process: Data Collection:

- The data were collected as previously described in Dileep (2019). Specifically, the dataset is publicly available on the Kaggle website, and it is from an ongoing cardiovascular study on residents of the town of Framingham, Massachusetts, and real data from. The classification goal is to predict whether the patient has a 10-year risk of future coronary heart disease (CHD). The dataset provides the patients’ information. It includes over 4,000 records and 15 attributes.
- Variables: Each attribute is a potential risk factor. There are both demographic, behavioral, and medical risk factors. Demographic such as (sex, age), Behavioral such as (currentSmoker, Risperdal), Medical whether history or current and Predict variable such as CHD.

**The Second Stage**

- Machine learning model: Machine learning has several algorithms that can be used for finding the relations among the data, it can be simply chosen based on the type of results (classification or regression). Thus, we have chosen the best algorithm for our result type which will be a fraction that indicates the user situation. We tried algorithms as SVM, Logistics regression, Naive Bayes, ANN and Decision Tree with optimization (SGD), and Splitting data to train and test split. In our framework, the the Streamline the Gradient Descent (SGD) algorithm using learning rate is learned as a part of the training process. These are based on saving information of the gradients result in previous iterations and using this information to improve updating the values in the current iteration. In this way, the descent of the gradient in those directions is enhanced that allow obtaining the best fitness function. In the first we have Used the SGD algorithm and update the weights as follows as shown in Framwork SGD Algorithm Optimization.
- DEEP LEARNING MODEL: We split the phases of our module into three phases: First phase is Data Preprocessing in which we clean the data from any invalid data or null values and drop them from the data, then We have normalized the data because of the data’s swerve. We normalized the data due to its skew by minimize the difference between values. It’s the easiest way in dealing with the data. Finally we use one—encoding that help us with categorical data of being indicated as a value and transfer it into the vectors. Second phase of our module is splitting the data and find the relation between them and the desired feature based on the part of training deep learning module. The rest part of the data used to test and calculate the accuracy of prediction. We have splited our data into 75% of training data and 25% testing data. Third phase of our data is creating the neural network module, neural module act as the nervous system of the human brain. It’s consists of layers the first layer called input layer, the last one called output layer and the middle ones called hidden layers, each layer has a number of neurons that connected with each other’s to generate a new neurons in a new hidden layer after passing into an activation function that turn the result into the final form. In our module we have used a one input layer with the number of our features as neurons, two hidden layers each one have 15 neurons and a one output layer have 2 neurons that 1 for the one who will have the heart attack, 0 for the one will haven’t. Also we used “RELU” as activation function at every because it’s the best activation function that result a best result. After passing the data into the used module to train and get a result and the accuracy was over 93.0% As the average of the values so it’s a great result in predicting which confirms the great benefit of deep learning.

1. The list of data structures used in each item contains: age, sex, name of the derivation and signal of length 256.

2. Each item in this list is evaluated.

3. For each diagnosis, a vector is obtained representing the nine diseases where all values are 0 minus the maximum value obtained.

4. An arithmetic mean of all diagnoses is made.

5. The bias of the model is filtered, for example the model has a clear deviation by which identifies the AVE1 as the vector has as maximum value and Minimum value AVE2.

6. Finally, a vector is returned where all values are zero minus those that are greater than 0.6. If none is greater than 0.6, it is set 1 the maximum value. With this algorithm and the model used, very good results are obtained. In the model with transformations, a bias filter is used between the normal class and the abnormal class. In our model with, no filters are used of biases.

The excellent results obtained in our model due to the evolution of precision during training. we have suggested using data generated from real data as shown in Appendix 2 in training avoid overfitting in the prediction. As you can see, each model has strengths in different classes. For the final framework, it has been decided to use both models(machine learning and deep learning). Our results have given half the importance to each (ML/DL) but finally, the result by DL achieved higher precision.

**Algorithm 1:** SGD algorithm optimization.

Initialization:initial weight vector *W*^(0)^
Parameter:learning rate η ≥ 0
For all *i* = 1, ……,*n* compute *g*_0_(*i*) ←∇ *fi*(*W*^(0)^)
For all *K* = 1,2, …… until convergence do

• Pick uniformly at random *i*_*k*_in 1, ……,*n*
• Compute ∇*f*_*ik*_(*W*^(*k*−1)^)
• Apply }{}W^{(k)} \leftarrow W^{(k-1)} - \eta (\nabla f_{ik}(W^{(k-1)}) - g_{k-1}(ik) + \frac{1}{n}\sum_{i=1}^{n} g_{k-1}(i))
• Store *g*_*k*_(*ik*) ←∇*f*_*ik*_(*W*^(*k*−1)^)
Output: Return last*W*^(*k*)^

**Algorithm 2:** Framework: diagnosis the heart diseases.

Data: model, ecg
*signals*,*n_derivations*,*diagnostic* = *newlist*()
*signals*,*age*, *sex*,*n_derivations* = *process_ecg*(*ecg*)
*diagnoses* = *model.evaluate*(*signs*,*age*, *sex*,*n_derivations*)
*Forddiagnoses*
*d* = *one_hot_con maximo*(*d*)
*diagnoses* = *arithmetic mean*(*diagnoses*)
*diagnoses* = *bias_filter*(*diagnostics*)
*diagnosis* = *get_diagnosis_with*−*threshold or maximum*(*diagnosis*)

## Experimental Result and Comparison

### Prepressing the dataset and transformation

It was superficially mentioned in “The Smart Healthcare Framework SHDML” when normalization was considered: to arrive at a good model you need to have a suitable data set as shown in Table 1. Thus, it is advisable not only that the data be normalized, but also that is be sufficient in quantity and diversity and that have been obtained from the same source or with a unified criterion. You could try to train the model with the data distribution that accurately reflects your data. However, having a uniform distribution is more than desirable to obtain a good model: that fulfills its function for all classes of diseases and generalize appropriately. To do this, in deep learning by generate more data from available data. To augment the data, a wide variety of techniques can be used such as Generative Adversarial Networks (GAN) can generate realistic data, which is beneficial to train the model. In our dataset total number of rows with missing values is 489, since it is only 11% of the entire dataset, the rows with missing values are excluded. We removed the missing values from the dataset as shown in Table 2. There is a set of good practices that allows to achieve more robust models, avoid overfitting and speed up training. One of them is normalization (Mnih & Teh, 2012).The input normalization allows the data to be scaled uniform in all directions, achieving a more symmetric J in all dimensions (w, b) as shown in Table 3. This makes the gradient descent algorithm easier to work with and avoids explosion or fading problems. A similar operation can be performed on the layers of a neural network, commonly known as batch normalization (Salimans & Kingma, 2016). The objective is pursued the normalization of the inputs. Given intermediate values of a layer of the neural network z, they are normalized with the mean and standard deviation.

**Table 1:** Sample of our dataset.

Age	Education	cigsPerDay	totChol	sysBP	diaBP	BMI	hearRate	gluco
39	4	0	195	106	70	26.97	80	77
46	2	0	250	121	81	28.73	95	76
48	1	20	245	127.5	80	25.34	75	70
61	3	30	225	150	95	28.58	65	103
46	3	23	285	130	84	23.1	85	85

Male	Currentsmoker	BPMeds	prevalentStroke	prevalentHyp	Diabetes	TenYearCHD
1	0	0	0	0	0	0
0	0	0	0	0	0	0
1	1	0	0	0	0	0
0	1	0	0	1	0	1
0	1	0	0	0	0	0

**Table 2:** Some of missing value in our dataset.

Sex-male	0
Age	0
currentSmoker	0
cigsPerDay	29
BPMeds	53
prevalentStroke	0
prevalentHyp	0
Diabetes	0
totChol	50
sysBP	0
diaBP	0
BMI	19
heartRate	1
Glucose	388
TenYearCHD	0

**Table 3:** Sample of our dataset normalization.

	C1 95% (2.5%)	C1 95% (97.5%)	Odds ratio	*p* Value
const	0.000043	0.000272	0.000109	0.000
Sex-male	1.455242	2.198536	1.788687	0.000
age	1.054483	1.080969	1.067644	0.000
cigsPerDay	1.011733	1.028128	1.019897	0.000
totChol	1.000158	1.004394	1.002273	0.035
sysBP	1.013292	1.021784	1.017529	0.000
glucose	1.004346	1.010898	1.007617	0.000

*Interpreting the results: Odds Ratio, Confidence Intervals and P values*

- This fitted model shows that, holding all other features constant, the odds of getting diagnosed with heart disease for males (sex-male = 1) over that of females (sex-male = 0) is exp(0.5815) = 1.788687. In terms of percent change, we can say that the odds for males are 78.8% higher than the odds for females.
- The coefficient for age says that, holding all others constant, we will see 7% increase in the odds of getting diagnosed with CDH for a 1 year increase in age since exp(0.0655) = 1.067644.
- Similarly, with every extra cigarette one smokes there is a 2% increase in the odds of CDH.
- For Total cholesterol level and glucose level there is no significant change.
- There is a 1.7% increase in odds for every unit increase in systolic Blood Pressure.

### Feature selection

- The calculated Logistic regression to anticipate the heart malady demonstrate in Dileep (2019) appears that holding all other highlights consistent, the chances of getting analyzed with heart infection for males (sex male = 1) over that of females (sex male = 0) is exp(0.5815) = 1.788687. In terms of percent alter, ready to say that the chances for guys are 78.8% higher than the chances for females.
- The coefficient for age says that, holding all others steady, we are going see 7% increment within the chances of getting analyzed with CDH for a one year increment in age since exp(0.0655) = 1.067644.
- Similarly, with each additional cigarette one smokes there’s a 2% increment within the chances of CDH.
- There’s no noteworthy alter for add up to cholesterol and glucose levels.
- There’s a 1.7% increment in chances for each unit increment in systolic blood pressure.

### Splitting data to train and test split

We split the data into outcomes and features.

- Outcomes are illustrated in the TenYearCHD column.
- Features are all columns excluded from TenYearCHD.
- The splitting ratio is 25% test to 75% train.

*Finding relations and patterns:* Machine learning has several algorithms that can be used for finding the relations among the data. It can be simply chosen based on the type of results (classification or regression), so we chose the Logistic Regression as the best algorithm for our result type which will be a fraction that indicates the user situation.

### Comparison between algorithms results

We tried algorithms as SVM, Logistics regression, Decision Tree, Naïve Bayes, ANN and SHDML.

**Figure 11:**
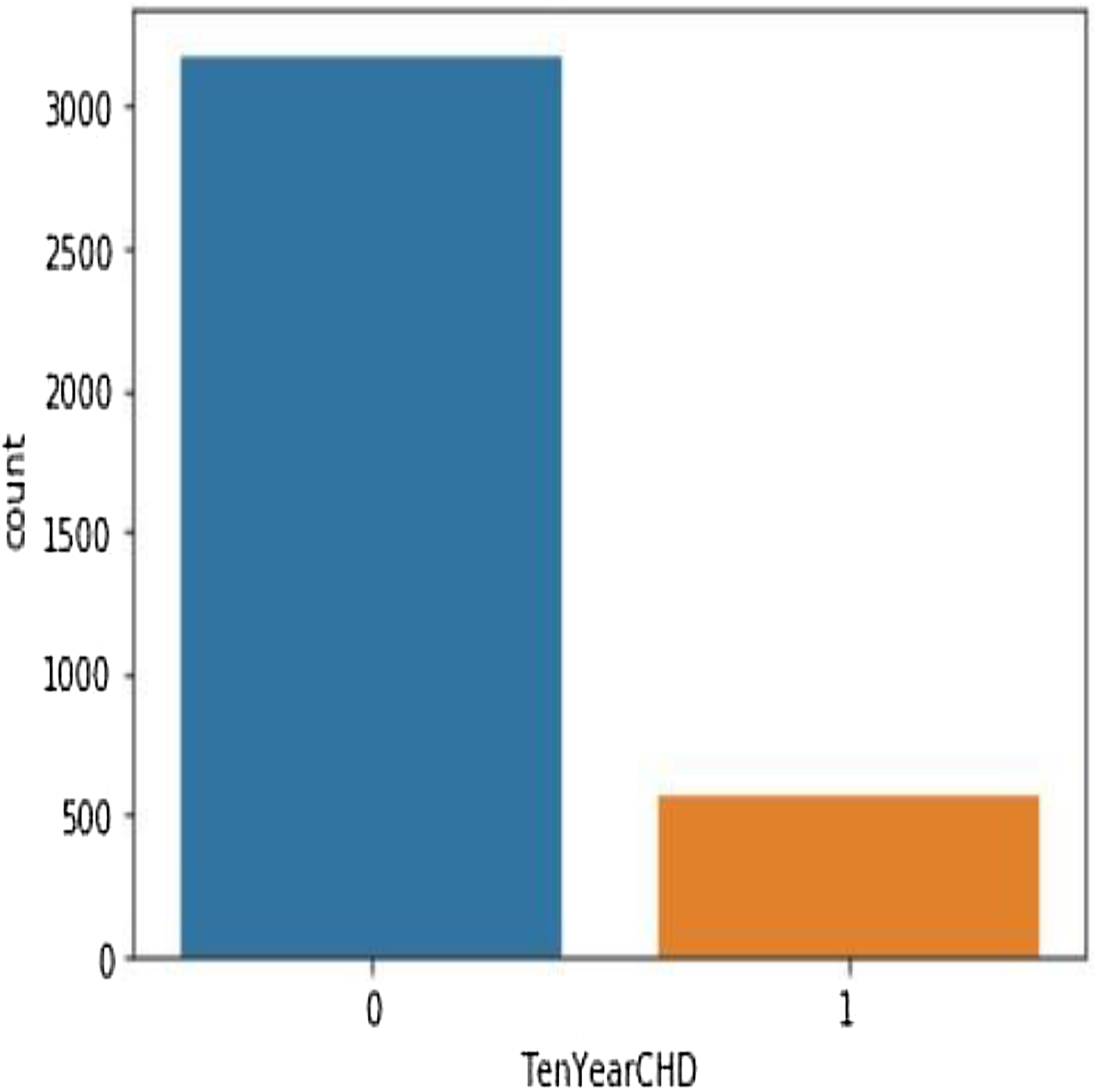
There are 3,179 patents with no heart disease and 572 patients with risk of heart disease.

**Figure 12:**
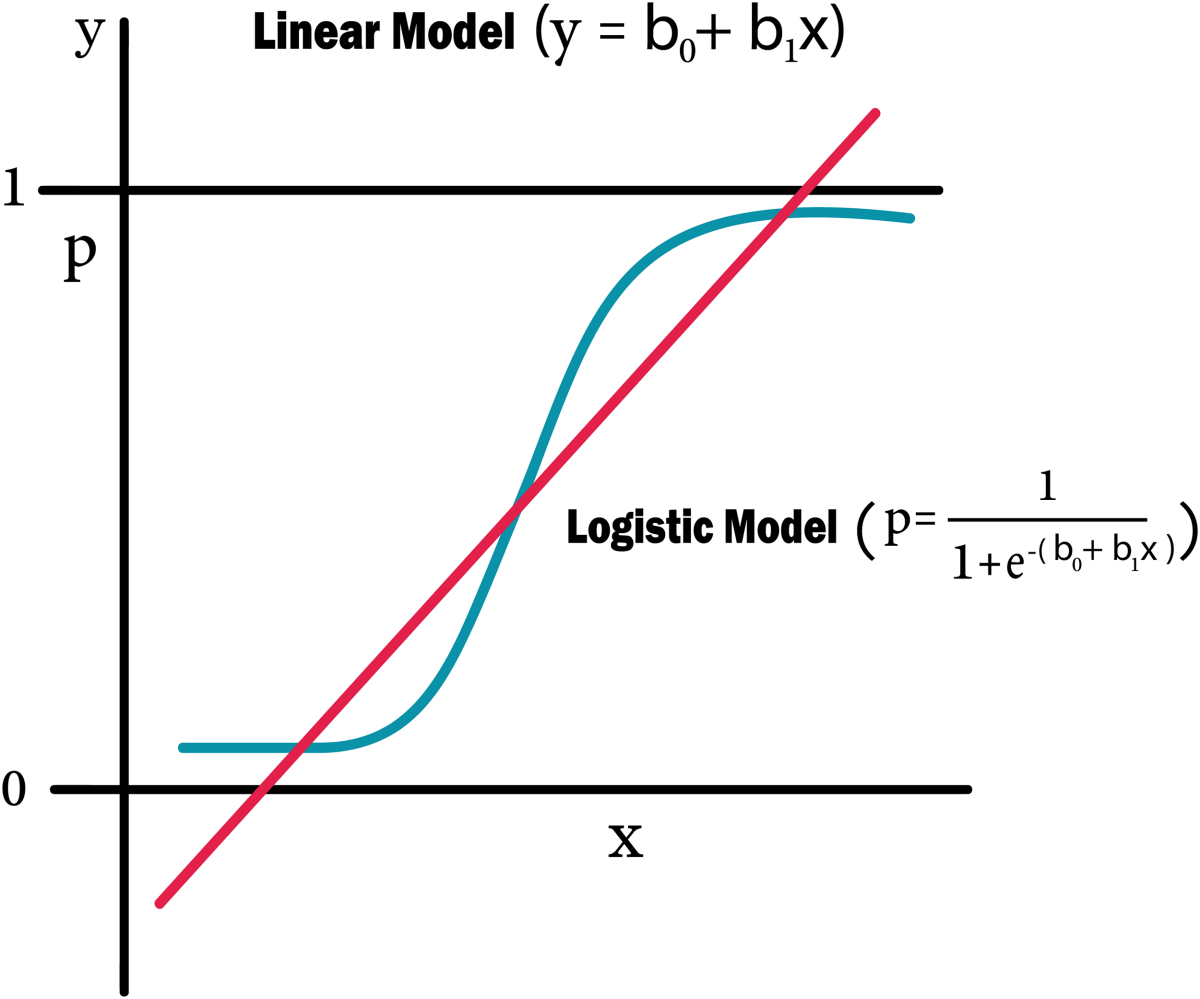
Logistic regression equation.

Table 4 shows the performance analysis of five machine learning algorithms. Comparison of the performance of various ML algorithms using python language. It has been observed that there is inconsistency between the results of the implementations. The performance analysis of Neural Network algorithm, simple implementation of deep learning architecture in python has resulted in improved performance in big dataset. From the numbers above we deduce that the algorithms that give the most accurate results are SHDML, ANN, SVM and the logistic Regression. From the numbers above we deduce that the algorithm that gives most accurate results is the logistic Regression (Eqs. (2) and (3)) (Preacher & Rucker, 2003). There are 3,179 patents with no heart disease and 572 patients with risk of heart disease, as shown in Fig. 11. This is Eq. (2) used in Logistic Regression as shown in the Fig. 12.

(2) \displaystyle{{{e^{{\beta _0} + {\beta _1}{X_1}}}} \over {1 + {e^{{\beta _0} + {\beta _1}{X_1}}}}}

when all features plugged in:

(3) \eqalignb{\matrix{ {\log it(p) = \log (p/(1 - p)) = {\beta _0} + {\beta _1} \times Sexmale + {\beta _2} \times age + {\beta _3} \times cigsPerDay + } \hfill \cr\hskip 3.7pc {{\beta _4} \times totChol + {\beta _5} \times sysBP + {\beta _6} \times glucose} \hfill \cr }}

**Table 4:** Comparison between machine learning Algorithms.

	SVM	Logistic regression	Decision tress	Naïve Bayes	ANN	SHDML
F1 score training	1.23	15.3	44.8	81.0	93.3	93.3
F1 score testing	2.12	11.6	13.8	80.0	92.0	92.0
Training accuracy	84	85	86.9	87.0	93.0	93.0
Testing accuracy	87.7	87.9	83.3	85.0	94.2	94.9

### Confusion matrix

**Figure 13:**
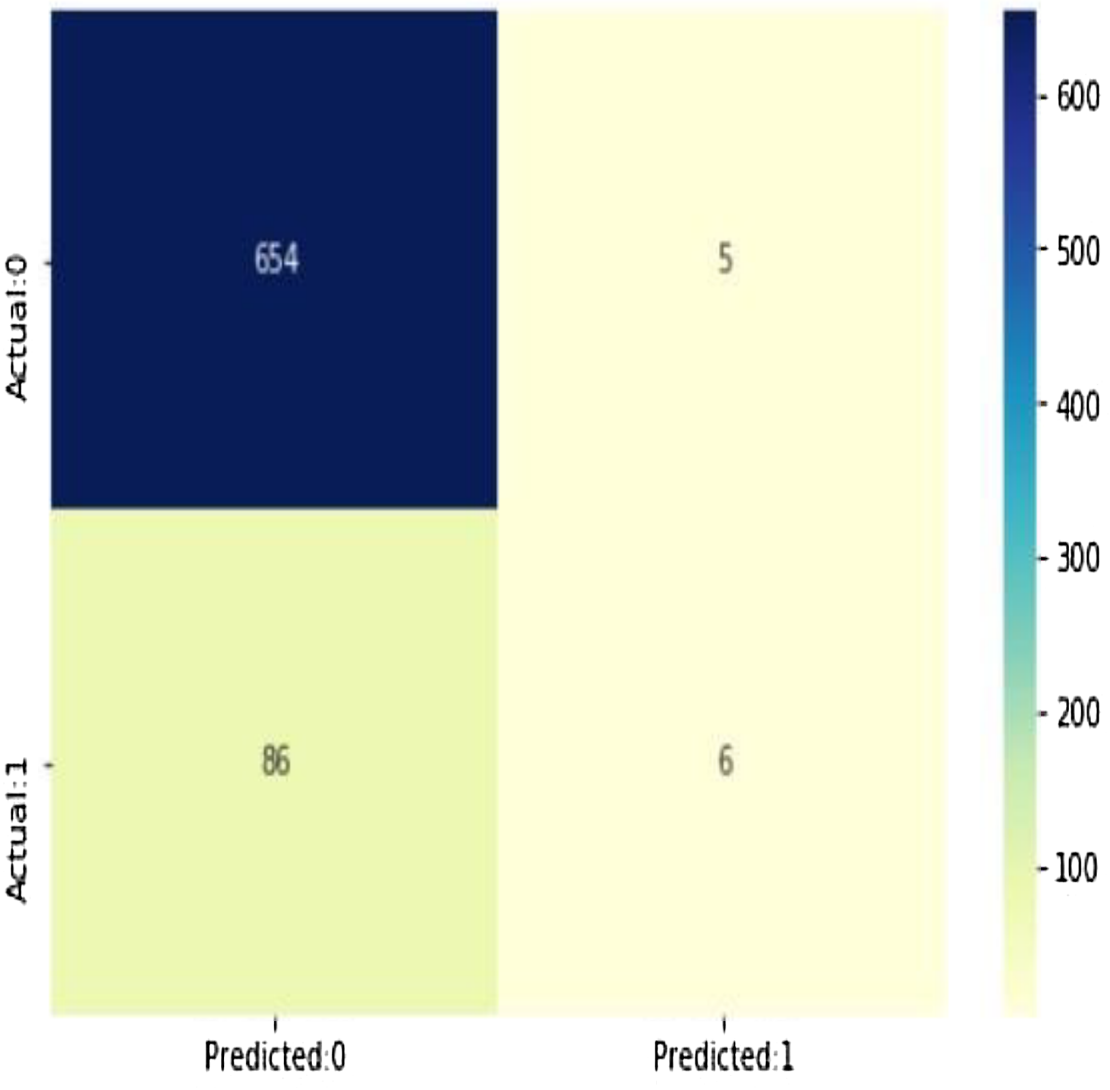
The confusion matrix shows 654 + 6 = 660 correct predictions and 5 + 86 = 91 incorrect ones.

As shown in Fig. 13, (true positives: 4, true negatives: 658, false positives: 1 (Type I error), false negatives: 88 (Type II error) model evolution statistics)

- The accuracy of the model = TP + TN/(TP + TN + FP + FN) = 0.8788282290279628
- The misclassification = 1 − Accuracy = 0.12117177097203724
- Sensitivity or true positive rate = TP/(TP + FN) = 0.06521739130434782
- Specificity or true negative rate = TN/(TN + FP) = 0.992412746585736
- Positive predictive value = TP/(TP + FP) = 0.5454545454545454
- Negative predictive value = TN/(TN + FN) = 0.8837837837837837
- Positive likelihood ratio = Sensitivity/(1 − Specificity) = 0.8595652173913052
- Negative likelihood ratio = (1 − Sensitivity)/Specificity = 0.9419292647254355

According to the above statistics it is clear that the model is highly specific more than being sensitive. The negative values are predicted more accurately than the positives as shown in Table 5. Training in the linear regression model will make it able to generate the best numeric function which will be used to predict the result, this function is characterized by being converged for the values of 0 or 1 which indicate the user situation with an accuracy of 88%.

**Table 5:** Sample of predicted probabilities results.

	Prob of no heart disease (0)	Prob of heart disease (1)
0	0.882486	0.117514
1	0.945754	0.054246
2	0.758568	0.241432
3	0.753151	0.246849
4	0.896659	0.103341

**Figure 14:**
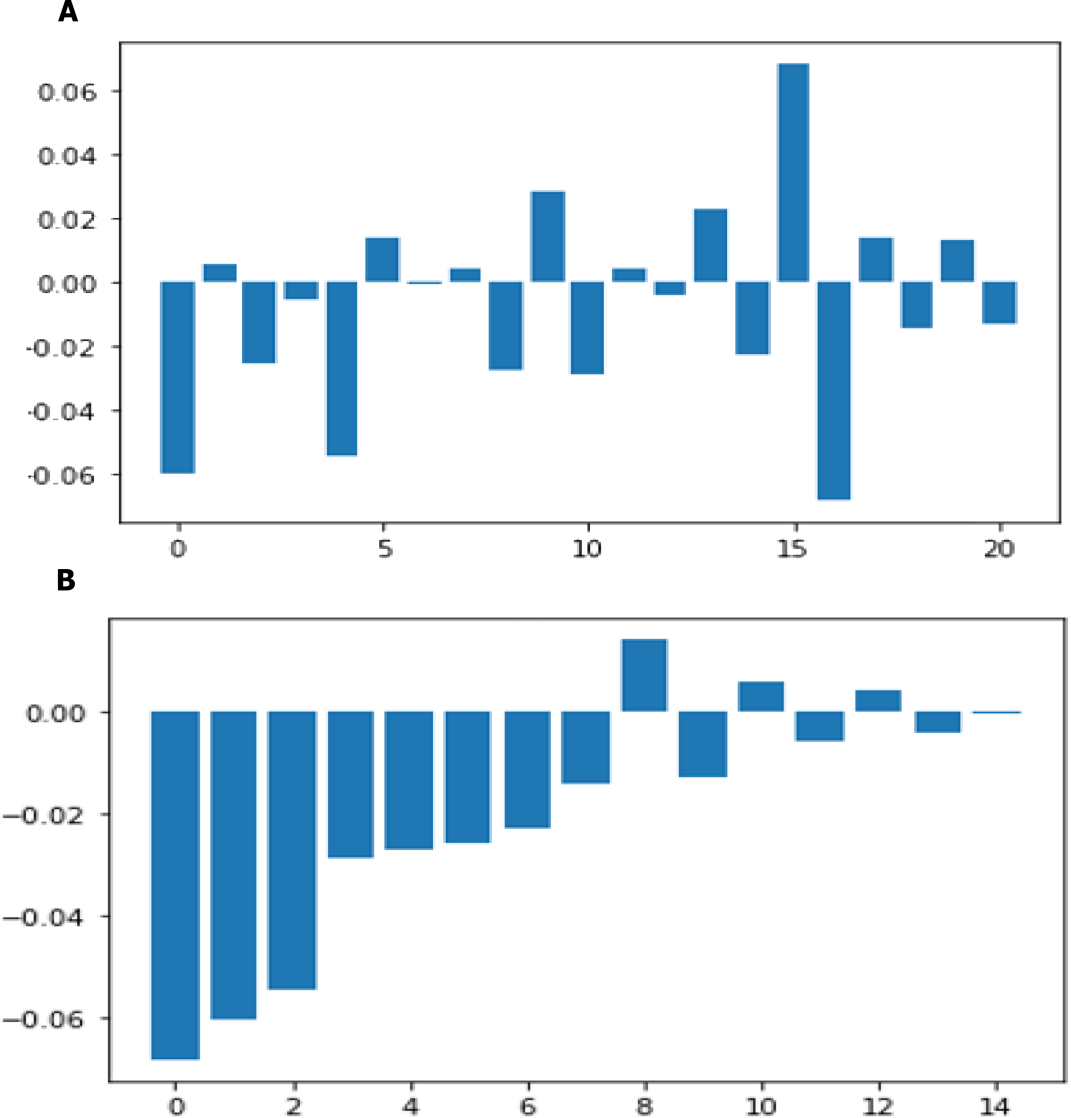
Feature importance before sorting and after sorting.

*Predicted probabilities of 0 (No Coronary Heart Disease) and 1 (Coronary Heart Disease: Yes)*. As shown in Fig. 14, the confusion matrix for decision trees shows 654 + 6 = 660 correct predictions and 5 + 86 = 91 incorrect ones for Cleveland dataset.

- All attributes selected after the elimination process show P values lower than 5% and thereby suggesting significant role in the heart disease prediction.
- TMen appear to be more helpless to heart malady than ladies. An increment in age, number of 546 cigarettes smoked per day and systolic blood weight moreover appear expanding chances of having 547 heart infections.
- Total cholesterol shows no significant change in the odds of CHD. This could be due to the presence of HDL (high-density lipoprotein), or ‘good’ cholesterol in the total cholesterol reading. Glucose too causes a very negligible change in odds (0.2%) The result of twenty one features sorted by their feature importance for dataset in machine learning as shown in Fig. 14A and deep learning in Fig. 14B.
- If we calculate the linear regression model as an example, we will find that it achieved an accuracy ratio representing 88%, the model is more specific than sensitive.
- Using our technique in addition to more data will boost the overall model, which is more specific than sensitive.

The SHDML has been trained in a virtual machine of the platform online Google Colab using size 64 batch training during 400 times for each model. This has been a training time of approximately 7 h. SGD was used as an optimizer with = 10 − 4, 1 = 0, 9 and 2 = 0, 999. Entropy is used as loss function cross-categorical, which is the standard loss function for classification in deep learning. When we used another sample, the data was somewhat larger in our SHDML framework which the the number of true positives in our sample data is 915, while 300 is the number of true negatives. 2 & 1 are the number of errors. Hence, if we calculate the accuracy where TP, FN, FP and TN represent the number of true positives, false negatives, false positives and true negatives. Accuracy = (TP + TN)/(TP + TN + FP + FN). Accuracy = (915 + 300)/(915 + 300 + 2 + 1) = 0.9975 = 99% accuracy.

### Evaluation machine learning algorithms for other dataset

**Figure 15:**
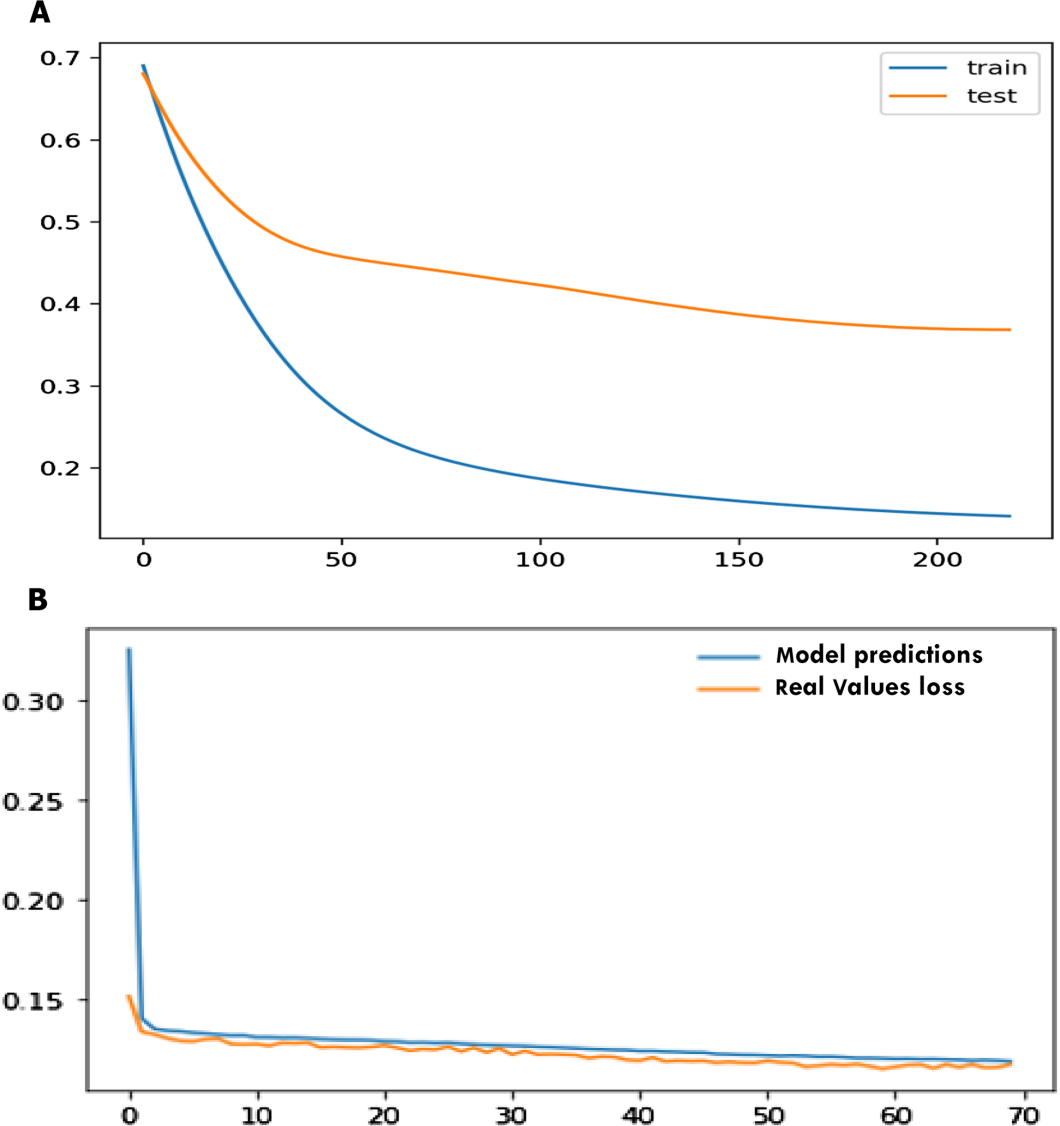
Early stopper and loss function graph to prediction in training and testing.

For the statlog dataset, the F1 score, sensitivity, specificity, accuracy for SVM, logistic regression (LR), decision tree (DT), naïve Bayes (NB), ANN, and SHDML are depicted in Tables 6 for validation, respectively. The accuracy for the Statlog dataset is shown in Table 6 are 97.04%, 95.0%, 97.4%, 95.37%, 97.8%, 98.9% for SVM, logistic regression (LR), decision tree (DT), naïve Bayes (NB), ANN, and SHDML, respectively. The accuracy for Statlog dataset value is 98.9%, for SHDML which is the best value. We can also see that test loss started to increase early 50 epochs as shown in Fig. 15A. This means that although the performance of the model has improved, we may not have the best performing or most stable model at the end of training. We are interested in saving the model with the best accuracy on the test dataset. We could also seek the model with the best loss on the test dataset, with the best accuracy. This highlights an important concept in model selection. The notion of the “best” model during training may conflict when evaluated using different performance measures. Try to choose models based on the metric by which they will be evaluated and presented in the domain. In a balanced binary classification problem, this will most likely be classification accuracy. Therefore, we will use accuracy on the validation to save the best method observed during training the loss function is directly related to the activation function used in the output layer of our framework to to prediction in training and testing as shown in Fig. 15B. The choice of fitness function is tightly coupled with the choice of output unit. Most of the time, we simply use the cross-entropy between the data distribution and the model distribution. The choice of how to represent the output then determines the form of the cross-entropy function.

**Table 6:** Comparison between machine learning algorithms for the Statlog dataset.

	SVM	Logistic regression	Decision tress	Naïve Bayes	ANN	SHDML
F1 score	96.0	96.0	90.0	92.0	98.0	98.3
Sensitivity	95.33	98.67	95.45	90.86	98.0	98.8
Specificity	97.28	92.50	92.42	88.78	95.0	95.46
Accuracy	97.04	95.0	97.4	95.37	97.8	98.9

For the Cleveland dataset, the F1 score, sensitivity, specificity, accuracy for SVM, logistic regression (LR), decision tree (DT), naïve Bayes (NB), ANN, and SHDML are depicted in Table 7 for validation, respectively. The accuracy for the cleveland dataset is shown in Table 7 are 95.00%, 90.0%, 91.0%, 89.8%, 93.0%, 98.0% for SVM, logistic regression (LR), decision tree (DT), naïve Bayes (NB), ANN, and SHDML, respectively. The accuracy for the Cleveland dataset value is 98.0%, for SHDML which is the best value.

**Table 7:** Comparison between machine learning algorithms for the Cleveland dataset.

	SVM	Logistic regression	Decision tress	Naïve Bayes	ANN	SHDML
F1 score	95.0	91.0	89.0	90.0	94.0	97.0
Sensitivity	94.0	91.0	92.0	90.0	92.0	95.0
Specificity	94.0	89.0	90.0	88.0	91.0	94.0
Accuracy	95.0	90.0	91.0	89.8	93.0	98.0

## Discussion and Conclusion

Wearable devices are now used for a wide range of health observation purposes. One of the most important elements of data collection is the sensor. In recent years, with improvements in semiconductor technology, the sensors have made a full range of parameters closer to realization. Wearable devices are becoming more and more popular in various fields, from sport and fitness to health surveillance. In particular, due to the increasing number of elderly people worldwide, wearable devices are becoming increasingly important for long-term health surveillance. The most important criteria in this study are the possibility of using a device in the real world, performance, efficiency and power consumption. In addition, we have considered the price of each device. Two key features are provided to the user in this project: First, an Android app that communicates with a wearable device that measures heart rate through Bluetooth communication. While the BPM of the user becomes unstable, the app sends SMS alerts to a mobile phone belonging to a medical expert or a patient’s family member, or their relatives via SMS containing the current location and patient status of the user. Second, Android/desktop that lets users know if they have heart disease or not by one click running a machine learning module that analyzes user data to detect heart disease. Other features include calculating daily steps and checking the nearest hospitals, cardiac centers, nearest health centers (GYMs) and the current location of users. We used the Java programming language, the Python programming language and the C-programming language to implement this. The C-programming language used to develop embedded devices using the embedded C version with Eclipse IDE which facilitates the development of the process. Java programming is used to develop Android apps with Android Studio 3 that provide a huge number of libraries. The Python programming language was used to develop the desktop app with the Anaconda Jupyter notebook. The machine learning and deep learning models, too. They have all used many important libraries. The project is determined by the merits of the system offered to the user. The merits of the project are as follows:

- Wearable device for calculation of heart rate.
- Real-time heart rate monitor.
- Actual daily steps count.
- Emergency SMS with current location.
- Prediction of high accuracy CHD.
- Real time database, storage and authentication on the Firebase cloud server.
- Nearest hospital, cardiac center and health care gyms.
- Provide the user with his medical history in order to monitor his or her health.

**Some of future works:**

There are some limitations to the current system that can be addressed as a future development:

- The embedded device board (ATmega32) will be reduced in size by printing another PCB board using the (ATmega8) microcontroller.
- Adding other sensors for wearable devices such as (temperature sensor, blood pressure sensor).
- Enhance the body of the wearable device.
- Enhance user interface to make it more user-friendly.
- Adding more features to the app.
- Improve the accuracy of the prediction model.

## Appendix 1

### Heart beat (primary data) algorithm

**Figure A1:**
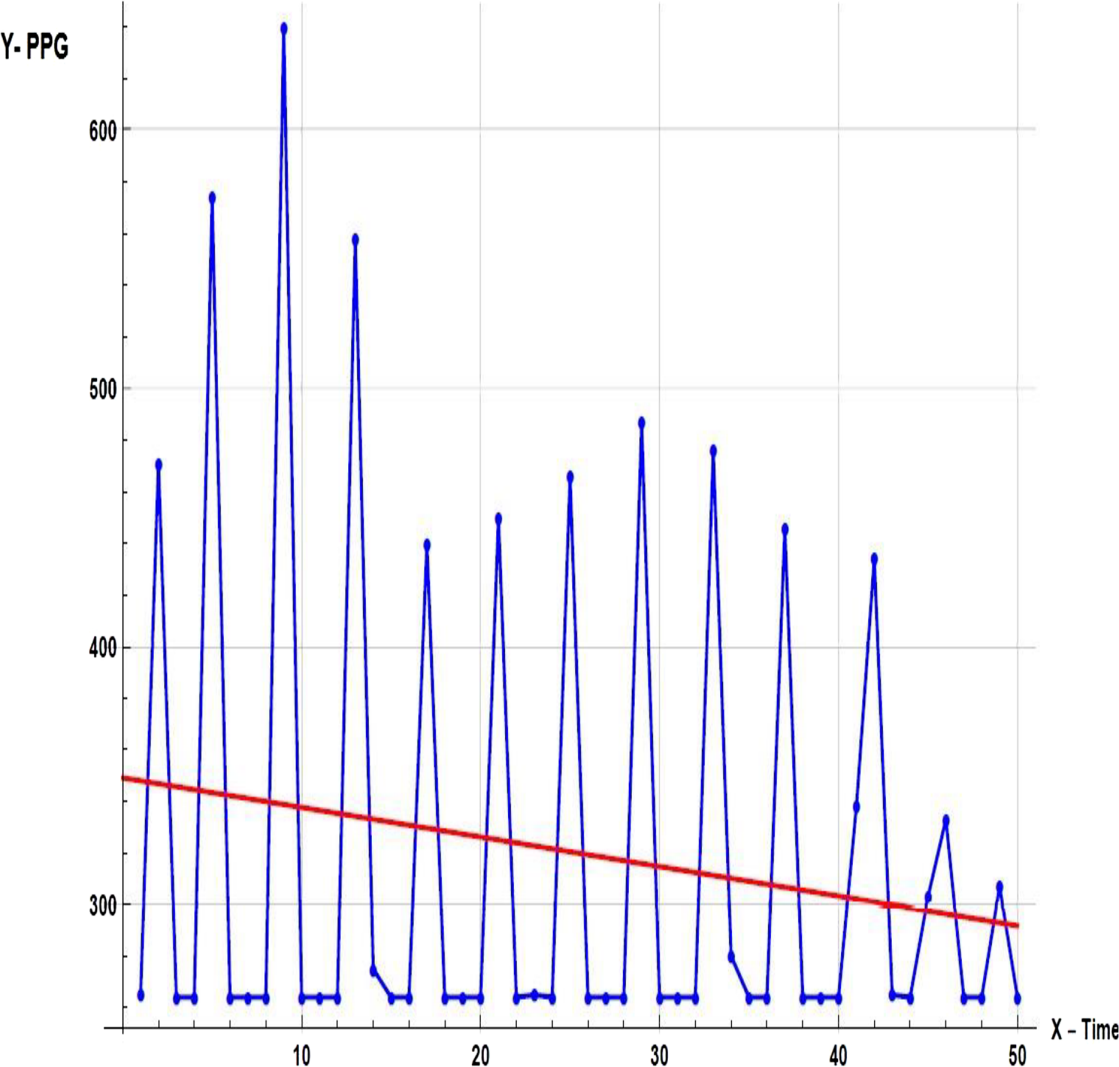
Sample of primary data.

First, it is important to have a regular sample rate with high enough resolution to get reliable measurement of the timing between each beat. To do this, we set up Timer0, an 8-bit hardware timer on the ATmega32, so that it throws an interrupt every other millisecond. That gives us a sample rate of 500 Hz, and beat-to-beat timing resolution of 2 mS. Readings as shown in Fig. A1, had a larger range. There is also a very noticeable second pulse after the main pulse. This second spike should be ignored by our application.

## Appendix 2

**Figure A2:**
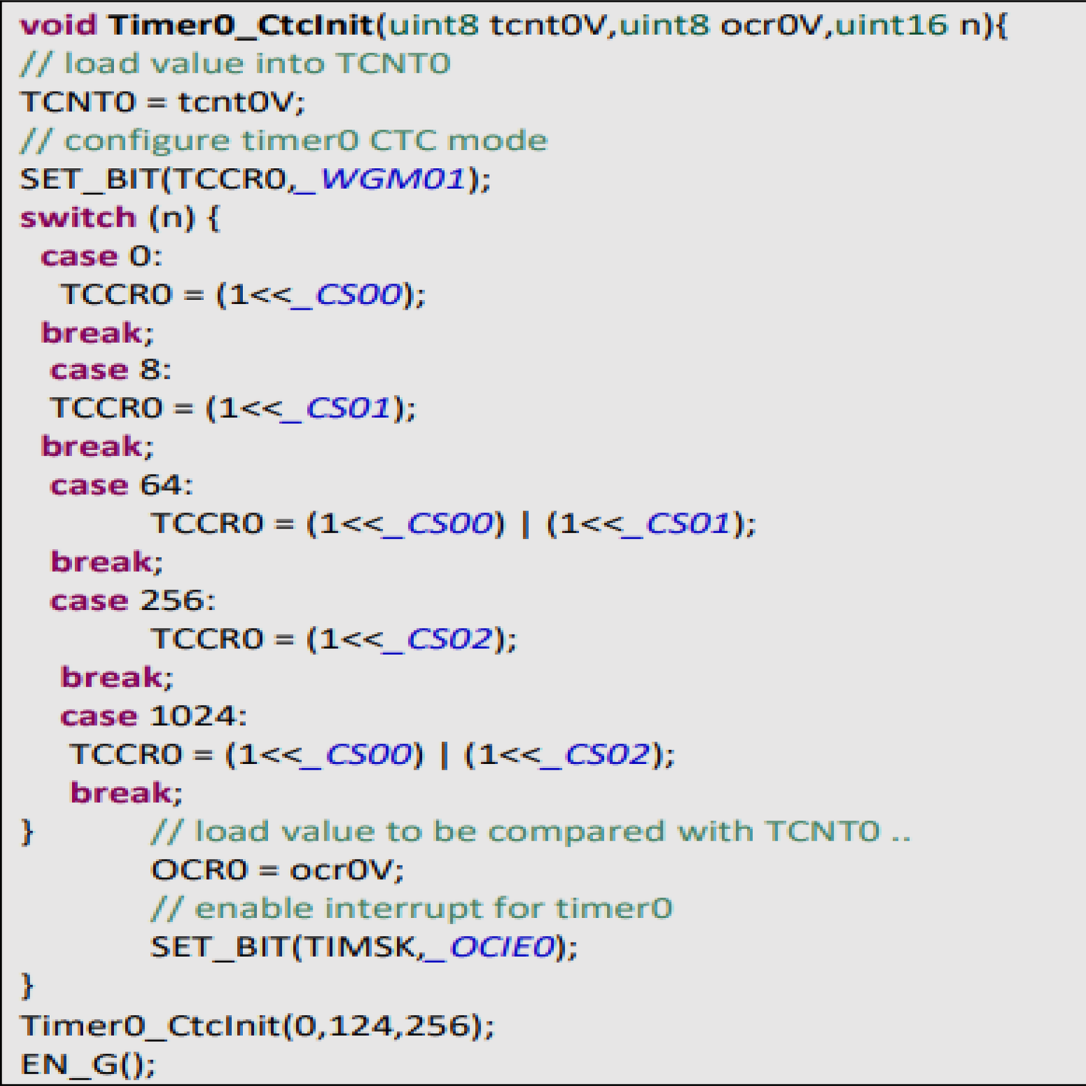
Heart beat algorithm.

- DESKTOP APP The desktop app is developed by Python programming language. The desktop app has the same features of Android app except BPM check and steps count. Our desktop app UI is designed using Tkinter library. It is the standard Python interface to the Tk GUI toolkit. After collecting the data, the next step is the heartbeat algorithm as shown in Fig. A2.

- Window: In different contexts this term has different meanings, but in general it refers to a rectangular area somewhere on the display screen of the user.

- Top level window: A window which exists on the screen independently. This will be designed to the screen manager with the usual frame and controls. It can be pushed around the screen, and can be resized as a rule.

- Widget: The generic term for all of the building blocks in a graphical user interface that make up an application.

- Core widgets: containers: frame, label, top-level, window panel. Buttons: button, button with radiobutton, button with checkbox and button with menu. Widgets for text: label, message, document. Widgets for entry: size, scrollbar, listbox, slider, spinbox, entry (single line), option menu, text (multiline), and canvas (vector and pixel graphics).

- Message Box: Tkinter offers three modules enabling the display of pop-up dialogs, tk.messagebox (confirmation, information, alert and error dialogs), tk.filedialog (single file, multiple file and directory dialogs) and tk.colorchooser (color picker).

- Frame: The Frame widget is the basic organizational unit for complex layouts at Tkinter. A frame is a rectangular region that may have other widgets in it.

- Child and parent: A parent-child relationship is formed when any widget is created. For instance, if you insert a text label inside a frame, the frame is the label parent.

Most important used libraries:

- Firebase: Used to connect to our project’s Firebase Cloud database and use their link string properties then initialize authentication, tables, and storage.

- Pyrebase: Used to build new account, log in, authenticate, verify, retrieve account data and update account data, hospitals, and heart centers.

- OS: This module offers a compact way to use functionalities based on the operating system. open() if you only want to read or write a file, if you want to modify paths, os.path module and see the file input module if you want to read all the lines of all the files on the command line. Used to select a new image profile from a given path.

- Urllib.request: Used in loading the user’s profile image of the current user profile image URL link from cloud firebase database.

- Pillow: The user’s profile image from cloud firebase database is used in preview.

- Requests: Makes it extremely easy to send HTTP/1.1 requests. There is no need to add query strings to your URLs manually, or form-encode your POST info. Because of urllib3. Used to detect user location when requesting information through an IP address.

- Web browser: a module provides a high-level interface which allows users to view Web-based documents. In certain cases, only calling the module’s open() function would do the right thing. Used to open a Google Maps web page on the default user browser for sites like gyms, hospitals, and cardiac centers nearby.

## Supplemental Information

10.7717/peerj-cs.646/supp-1 Supplemental Information 1 Source Code for Machine Learning Model. Source code to get the expected functionality of the project, the application has been programmed by dividing it into Two platforms. ATmega32 is the one which is collect heart beat from sensor and send BPM into android app using Bluetooth communication , the other side android app that receive BPM and path it to Machine learning module to detect heart disease

10.7717/peerj-cs.646/supp-2 Supplemental Information 2 Source Code for Deep Learning Model. The section of source code to deep learning which our system has been programmed by dividing it into Two platforms. ATmega32 is the one which is collect heart beat from sensor and send BPM into android app using Bluetooth communication , the other side android app that receive BPM and path it to Deep learning module to predict heart disease

10.7717/peerj-cs.646/supp-3 Supplemental Information 3 framingham_Dataset.csv. Real-time Database − Firebase supports JSON data and all users connected to it receive live updates after every change.The systeam have a regular sample rate with h-reliable measurement of the timing between each beat. To do this, we set up Timer0, an 8bit hardware timer on the ATmega32, so that it throws an interrupt every other millisecond.beat timing resolution of 2mS.-to-That gives us a sample rate of 500Hz.

10.7717/peerj-cs.646/supp-4 Supplemental Information 4 Machine_Learning_Model.html. CHD Prediction of user’s inputs using Machine Learning model with logistic Regression

10.7717/peerj-cs.646/supp-5 Supplemental Information 5 Deep Learning Model. Coronary heart disease is often caused by the buildup of plaque, a waxy substance, inside the lining of larger coronary arteries. This unique service which predict coronary heart disease for user using Deep Learning module. User insert some data to module using editTexts in page and click on button finally result will be displayed.

10.7717/peerj-cs.646/supp-6 Supplemental Information 6 Statlog_Dataset. This database contains 13 attributes (which have been extracted from a larger set of 75 Attribute Information: ------------------------ -- 1. age -- 2. sex -- 3. chest pain type 4 values -- 4. resting blood pressure -- 5. serum cholestoral in mg/dl -- 6. fasting blood sugar > 120 mg/dl -- 7. resting electrocardiographic results values 0,1,2 -- 8. maximum heart rate achieved -- 9. exercise induced angina -- 10. oldpeak = ST depression induced by exercise relative to rest -- 11. the slope of the peak exercise ST segment -- 12. number of major vessels (0-3) colored by flourosopy -- 13. thal: 3 = normal; 6 = fixed defect; 7 = reversable defect Attributes types ----------------- Real: 1,4,5,8,10,12 Ordered:11, Binary: 2,6,9 Nominal:7,3,13 Variable to be predicted ------------------------ Absence (1) or presence (2) of heart disease Cost Matrix abse pres absence 0 1 presence 5 0 where the rows represent the true values and the columns the predicted. No missing values. 270 observations

10.7717/peerj-cs.646/supp-7 Supplemental Information 7 Cleveland dataset. Cleveland dataset This database contains 76 attributes, but all published experiments refer to using a subset of 14 of them. In particular, the Cleveland database is the only one that has been used by ML researchers to this date. The "goal" field refers to the presence of heart disease in the patient. It is integer valued from 0 (no presence) to 4. Experiments with the Cleveland database have concentrated on simply attempting to distinguish presence (values 1,2,3,4) from absence (value 0). The names and social security numbers of the patients were recently removed from the database, replaced with dummy values. One file has been "processed", that one containing the Cleveland database. All four unprocessed files also exist in this directory. To see Test Costs (donated by Peter Turney), please see the folder "Costs" Attribute Information: Only 14 attributes used: 1. #3 (age) 2. #4 (sex) 3. #9 (cp) 4. #10 (trestbps) 5. #12 (chol) 6. #16 (fbs) 7. #19 (restecg) 8. #32 (thalach) 9. #38 (exang) 10. #40 (oldpeak) 11. #41 (slope) 12. #44 (ca) 13. #51 (thal) 14. #58 (num) (the predicted attribute)

10.7717/peerj-cs.646/supp-8 Supplemental Information 8 Source Code. Description of our system SHDML. We used python programming language for developing our desktop application: 1. Code is easy to read, use and maintain The effectiveness of the application greatly depends on the quality of its source code. 2. Supports multiple programming paradigms Python is constructed with the aim to help developers write logical and clean code for both large-scale and small-scale projects. Procedural programming, Object-oriented programming, Functional programming 3. Compatible with Major Platforms and Systems Python supports all the major operating systems and architectures. Being an interpreted language, Python offers the following benefits over compiled programming languages such as C, C++, Java, etc: It is easier to run the same Python program on multiple platforms, including Windows, Linux, macOS, etc. Since Python code is executed line-by-line, instead of all at once, it is easy to make alterations in the code and run the modified code and see the impact of changes immediately in the result.

## Additional Information and Declarations

### Data Availability

The following information was supplied regarding data availability: The data and code are available in the Supplemental Files.
