# Supplementary material for: A new smart healthcare framework for real-time heart disease detection based on deep and machine learning
Source: PeerJ Comput Sci. 2021 Jul 28;7:e646. doi: 10.7717/peerj-cs.646 (PMC8330430; doi:10.7717/peerj-cs.646)
Supplement: Supplemental Information 4 — CHD Prediction of user’s inputs using Machine Learning model with logistic Regression [file peerj-cs-07-646-s004.html]

Heart Disease Prediction Model


In [2]:

```
import pandas as pd
import numpy as np
import matplotlib.pyplot as Plt
import seaborn as sn
from sklearn.metrics import confusion_matrix
import matplotlib.mlab as mlab
%matplotlib inline
```

In [3]:

```
heart_df=pd.read_csv("framingham.csv")
heart_df.drop(['education'],axis=1,inplace=True)
heart_df.head()
```

Out[3]:

|  | male | age | currentSmoker | cigsPerDay | BPMeds | prevalentStroke | prevalentHyp | diabetes | totChol | sysBP | diaBP | BMI | heartRate | glucose | TenYearCHD |
| --- | --- | --- | --- | --- | --- | --- | --- | --- | --- | --- | --- | --- | --- | --- | --- |
| 0 | 1 | 39 | 0 | 0.0 | 0.0 | 0 | 0 | 0 | 195.0 | 106.0 | 70.0 | 26.97 | 80.0 | 77.0 | 0 |
| 1 | 0 | 46 | 0 | 0.0 | 0.0 | 0 | 0 | 0 | 250.0 | 121.0 | 81.0 | 28.73 | 95.0 | 76.0 | 0 |
| 2 | 1 | 48 | 1 | 20.0 | 0.0 | 0 | 0 | 0 | 245.0 | 127.5 | 80.0 | 25.34 | 75.0 | 70.0 | 0 |
| 3 | 0 | 61 | 1 | 30.0 | 0.0 | 0 | 1 | 0 | 225.0 | 150.0 | 95.0 | 28.58 | 65.0 | 103.0 | 1 |
| 4 | 0 | 46 | 1 | 23.0 | 0.0 | 0 | 0 | 0 | 285.0 | 130.0 | 84.0 | 23.10 | 85.0 | 85.0 | 0 |

In [4]:

```
heart_df.isnull().sum()
```

Out[4]:

```
male                 0
age                  0
currentSmoker        0
cigsPerDay          29
BPMeds              53
prevalentStroke      0
prevalentHyp         0
diabetes             0
totChol             50
sysBP                0
diaBP                0
BMI                 19
heartRate            1
glucose            388
TenYearCHD           0
dtype: int64
```

In [5]:

```
heart_df.dropna(axis=0,inplace=True)
```

## Exploratory Analysis

In [6]:

```
'''def draw_histograms(dataframe, features, rows, cols):
    fig=Plt.figure(figsize=(20,20))
    for i, feature in enumerate(features):
        ax=fig.add_subplot(rows,cols,i+1)
        dataframe[feature].hist(bins=20,ax=ax,facecolor='midnightblue')
        ax.set_title(feature+" Distribution",color='DarkRed')
        
    fig.tight_layout()  
    Plt.show()
draw_histograms(heart_df,heart_df.columns,6,3)'''
```

Out[6]:

```
'def draw_histograms(dataframe, features, rows, cols):\n    fig=Plt.figure(figsize=(20,20))\n    for i, feature in enumerate(features):\n        ax=fig.add_subplot(rows,cols,i+1)\n        dataframe[feature].hist(bins=20,ax=ax,facecolor=\'midnightblue\')\n        ax.set_title(feature+" Distribution",color=\'DarkRed\')\n        \n    fig.tight_layout()  \n    Plt.show()\ndraw_histograms(heart_df,heart_df.columns,6,3)'
```

In [7]:

```
sn.countplot(x='TenYearCHD',data=heart_df)
```

Out[7]:

```
<matplotlib.axes._subplots.AxesSubplot at 0x26ad68d2c48>
```

There are 3179 patents with no heart disease and 572 patients with risk of heart disease.

In [8]:

```
heart_df.describe()
```

Out[8]:

|  | male | age | currentSmoker | cigsPerDay | BPMeds | prevalentStroke | prevalentHyp | diabetes | totChol | sysBP | diaBP | BMI | heartRate | glucose | TenYearCHD |
| --- | --- | --- | --- | --- | --- | --- | --- | --- | --- | --- | --- | --- | --- | --- | --- |
| count | 3751.000000 | 3751.000000 | 3751.000000 | 3751.000000 | 3751.000000 | 3751.000000 | 3751.000000 | 3751.000000 | 3751.000000 | 3751.000000 | 3751.000000 | 3751.000000 | 3751.000000 | 3751.000000 | 3751.000000 |
| mean | 0.445215 | 49.573447 | 0.488403 | 9.008531 | 0.030392 | 0.005599 | 0.311917 | 0.027193 | 236.928019 | 132.368435 | 82.938550 | 25.808288 | 75.704079 | 81.880032 | 0.152493 |
| std | 0.497056 | 8.570204 | 0.499932 | 11.925097 | 0.171686 | 0.074623 | 0.463338 | 0.162666 | 44.611594 | 22.046522 | 11.932779 | 4.065599 | 11.956382 | 23.882233 | 0.359546 |
| min | 0.000000 | 32.000000 | 0.000000 | 0.000000 | 0.000000 | 0.000000 | 0.000000 | 0.000000 | 113.000000 | 83.500000 | 48.000000 | 15.540000 | 44.000000 | 40.000000 | 0.000000 |
| 25% | 0.000000 | 42.000000 | 0.000000 | 0.000000 | 0.000000 | 0.000000 | 0.000000 | 0.000000 | 206.000000 | 117.000000 | 75.000000 | 23.085000 | 68.000000 | 71.000000 | 0.000000 |
| 50% | 0.000000 | 49.000000 | 0.000000 | 0.000000 | 0.000000 | 0.000000 | 0.000000 | 0.000000 | 234.000000 | 128.000000 | 82.000000 | 25.410000 | 75.000000 | 78.000000 | 0.000000 |
| 75% | 1.000000 | 56.000000 | 1.000000 | 20.000000 | 0.000000 | 0.000000 | 1.000000 | 0.000000 | 264.000000 | 144.000000 | 90.000000 | 28.060000 | 82.000000 | 87.000000 | 0.000000 |
| max | 1.000000 | 70.000000 | 1.000000 | 70.000000 | 1.000000 | 1.000000 | 1.000000 | 1.000000 | 696.000000 | 295.000000 | 142.500000 | 56.800000 | 143.000000 | 394.000000 | 1.000000 |

#### Logistic regression equation

$$P=\hspace{.2cm}e^{\beta\_0 + \beta\_1 X\_1}\hspace{.2cm}/\hspace{.2cm}1+e^{\beta\_0 +\beta\_1 X\_1}$$

When all features plugged in:

$$logit(p) = log(p/(1-p))=\beta\_0 +\beta\_1\hspace{.1cm} \*\hspace{.2cm} Sexmale\hspace{.2cm}+\beta\_2\hspace{.1cm} \* \hspace{.1cm}age\hspace{.2cm}+\hspace{.2cm}\beta\_3\hspace{.1cm} \*\hspace{.1cm} cigsPerDay\hspace{.2cm}+\hspace{.2cm}\beta\_4 \hspace{.1cm}\*\hspace{.1cm} totChol\hspace{.2cm}+\hspace{.2cm}\beta\_5\hspace{.1cm} \*\hspace{.1cm} sysBP\hspace{.2cm}+\hspace{.2cm}\beta\_6\hspace{.1cm} \*\hspace{.1cm} glucose\hspace{.2cm}$$

- **This fitted model shows that, holding all other features constant, the odds of getting diagnosed with heart disease for males (sex\_male = 1)over that of females (sex\_male = 0) is exp(0.5815) = 1.788687. In terms of percent change, we can say that the odds for males are 78.8% higher than the odds for females.**
- **The coefficient for age says that, holding all others constant, we will see 7% increase in the odds of getting diagnosed with CDH for a one year increase in age since exp(0.0655) = 1.067644.**
- **Similarly , with every extra cigarette one smokes thers is a 2% increase in the odds of CDH.**
- **For Total cholosterol level and glucose level there is no significant change.**
- **There is a 1.7% increase in odds for every unit increase in systolic Blood Pressure.**

In [9]:

```
import sklearn
from sklearn.model_selection import train_test_split

outcomes = heart_df['TenYearCHD']
features = heart_df.drop('TenYearCHD', axis = 1)

x_train,x_test,y_train,y_test=train_test_split(features,outcomes,test_size=.20,random_state=5)
```

In [10]:

```
from sklearn.linear_model import LogisticRegression
from sklearn.metrics import accuracy_score
from sklearn.metrics import f1_score

logreg=LogisticRegression(max_iter=1200,C=0.01,random_state=4)
logreg.fit(x_train,y_train)
y_train_pred=logreg.predict(x_train)
y_test_pred=logreg.predict(x_test)

print('The training F1 Score is', f1_score(y_train_pred, y_train))
print('The testing F1 Score is', f1_score(y_test_pred, y_test))
train_accuracy = accuracy_score(y_train, y_train_pred)
test_accuracy = accuracy_score(y_test, y_test_pred)
print('The training accuracy is', train_accuracy)
print('The test accuracy is', test_accuracy)
logreg
```

```
The training F1 Score is 0.15327102803738316
The testing F1 Score is 0.11650485436893204
The training accuracy is 0.849
The test accuracy is 0.8788282290279628
```

Out[10]:

```
LogisticRegression(C=0.01, class_weight=None, dual=False, fit_intercept=True,
                   intercept_scaling=1, l1_ratio=None, max_iter=1200,
                   multi_class='auto', n_jobs=None, penalty='l2',
                   random_state=4, solver='lbfgs', tol=0.0001, verbose=0,
                   warm_start=False)
```

In [11]:

```
from sklearn.tree import DecisionTreeClassifier
from sklearn.metrics import accuracy_score
from sklearn.metrics import f1_score

decTree=DecisionTreeClassifier(max_depth=10,min_samples_leaf=9, min_samples_split=3)
decTree.fit(x_train,y_train)
y_train_pred=decTree.predict(x_train)
y_test_pred=decTree.predict(x_test)

print('The training F1 Score is', f1_score(y_train_pred, y_train))
print('The testing F1 Score is', f1_score(y_test_pred, y_test))
train_accuracy = accuracy_score(y_train, y_train_pred)
test_accuracy = accuracy_score(y_test, y_test_pred)
print('The training accuracy is', train_accuracy)
print('The test accuracy is', test_accuracy)
decTree
```

```
The training F1 Score is 0.45594405594405596
The testing F1 Score is 0.13986013986013987
The training accuracy is 0.8703333333333333
The test accuracy is 0.8362183754993342
```

Out[11]:

```
DecisionTreeClassifier(ccp_alpha=0.0, class_weight=None, criterion='gini',
                       max_depth=10, max_features=None, max_leaf_nodes=None,
                       min_impurity_decrease=0.0, min_impurity_split=None,
                       min_samples_leaf=9, min_samples_split=3,
                       min_weight_fraction_leaf=0.0, presort='deprecated',
                       random_state=None, splitter='best')
```

In [12]:

```
from sklearn.svm import SVC
from sklearn.metrics import accuracy_score
from sklearn.metrics import f1_score

svm=SVC()
svm.fit(x_train,y_train)
y_train_pred=svm.predict(x_train)
y_test_pred=svm.predict(x_test)

print('The training F1 Score is', f1_score(y_train_pred, y_train))
print('The testing F1 Score is', f1_score(y_test_pred, y_test))
train_accuracy = accuracy_score(y_train, y_train_pred)
test_accuracy = accuracy_score(y_test, y_test_pred)
print('The training accuracy is', train_accuracy)
print('The test accuracy is', test_accuracy)
svm
```

```
The training F1 Score is 0.012371134020618558
The testing F1 Score is 0.021276595744680847
The training accuracy is 0.8403333333333334
The test accuracy is 0.877496671105193
```

Out[12]:

```
SVC(C=1.0, break_ties=False, cache_size=200, class_weight=None, coef0=0.0,
    decision_function_shape='ovr', degree=3, gamma='scale', kernel='rbf',
    max_iter=-1, probability=False, random_state=None, shrinking=True,
    tol=0.001, verbose=False)
```

In [13]:

```
from sklearn.neural_network import MLPClassifier
from sklearn.metrics import f1_score
from sklearn.metrics import accuracy_score

nn = MLPClassifier(activation='relu', solver='adam', learning_rate_init=.01, learning_rate='constant', 
                      max_iter=1200, early_stopping=False, n_iter_no_change=10, shuffle=True, validation_fraction=.1,
                      random_state=42, batch_size=32, hidden_layer_sizes=(100,))
nn.fit(x_train,y_train)

y_train_pred = nn.predict(x_train)
y_test_pred = nn.predict(x_test)

print('The training F1 Score is', f1_score(y_train_pred, y_train))
print('The testing F1 Score is', f1_score(y_test_pred, y_test))
train_accuracy = accuracy_score(y_train, y_train_pred)
test_accuracy = accuracy_score(y_test, y_test_pred)
print('The training accuracy is', train_accuracy)
print('The test accuracy is', test_accuracy)
nn
```

```
The training F1 Score is 0.02862985685071575
The testing F1 Score is 0.06249999999999999
The training accuracy is 0.8416666666666667
The test accuracy is 0.8801597869507324
```

Out[13]:

```
MLPClassifier(activation='relu', alpha=0.0001, batch_size=32, beta_1=0.9,
              beta_2=0.999, early_stopping=False, epsilon=1e-08,
              hidden_layer_sizes=(100,), learning_rate='constant',
              learning_rate_init=0.01, max_fun=15000, max_iter=1200,
              momentum=0.9, n_iter_no_change=10, nesterovs_momentum=True,
              power_t=0.5, random_state=42, shuffle=True, solver='adam',
              tol=0.0001, validation_fraction=0.1, verbose=False,
              warm_start=False)
```

In [ ]:

```

```

#### Accuracy of the model is 0.8788

In [14]:

```
from sklearn.metrics import confusion_matrix
cm=confusion_matrix(y_test,y_test_pred)
conf_matrix=pd.DataFrame(data=cm,columns=['Predicted:0','Predicted:1'],index=['Actual:0','Actual:1'])
Plt.figure(figsize = (8,5))
sn.heatmap(conf_matrix, annot=True,fmt='d',cmap="YlGnBu")
```

Out[14]:

```
<matplotlib.axes._subplots.AxesSubplot at 0x26ad70748c8>
```

The confusion matrix shows 658+4 = 662 correct predictions and 88+1= 89 incorrect ones.

**True Positives:** 4

**True Negatives:** 658

**False Positives:** 1 (*Type I error*)

**False Negatives:** 88 ( *Type II error*)

In [15]:

```
TN=cm[0,0]
TP=cm[1,1]
FN=cm[1,0]
FP=cm[0,1]
sensitivity=TP/float(TP+FN)
specificity=TN/float(TN+FP)
```

In [16]:

```
print('The acuuracy of the model = TP+TN/(TP+TN+FP+FN) = ',(TP+TN)/float(TP+TN+FP+FN),'\n',

'The Missclassification = 1-Accuracy = ',1-((TP+TN)/float(TP+TN+FP+FN)),'\n',

'Sensitivity or True Positive Rate = TP/(TP+FN) = ',TP/float(TP+FN),'\n',

'Specificity or True Negative Rate = TN/(TN+FP) = ',TN/float(TN+FP),'\n',

'Positive Predictive value = TP/(TP+FP) = ',TP/float(TP+FP),'\n',

'Negative predictive Value = TN/(TN+FN) = ',TN/float(TN+FN),'\n',

'Positive Likelihood Ratio = Sensitivity/(1-Specificity) = ',sensitivity/(1-specificity),'\n',

'Negative likelihood Ratio = (1-Sensitivity)/Specificity = ',(1-sensitivity)/specificity)
```

```
The acuuracy of the model = TP+TN/(TP+TN+FP+FN) =  0.8801597869507324 
 The Missclassification = 1-Accuracy =  0.11984021304926762 
 Sensitivity or True Positive Rate = TP/(TP+FN) =  0.03260869565217391 
 Specificity or True Negative Rate = TN/(TN+FP) =  0.9984825493171472 
 Positive Predictive value = TP/(TP+FP) =  0.75 
 Negative predictive Value = TN/(TN+FN) =  0.8808567603748326 
 Positive Likelihood Ratio = Sensitivity/(1-Specificity) =  21.48913043478263 
 Negative likelihood Ratio = (1-Sensitivity)/Specificity =  0.9688615038985067
```

- \*\*All attributes selected after the elimination process show Pvalues lower than 5% and thereby suggesting significant role in the Heart disease prediction.\*\*
  
  
- \*\*Men seem to be more susceptible to heart disease than women.Increase in Age,number of cigarettes smoked per day and systolic Blood Pressure also show increasing odds of having heart disease.\*\*
  
  
- \*\*Total cholesterol shows no significant change in the odds of CHD. This could be due to the presence of 'good cholesterol(HDL) in the total cholesterol reading.Glucose too causes a very negligible change in odds (0.2%)\*\*
  
  
- \*\*The model predicted with 0.88 accuracy. The model is more specific than sensitive.\*\*
  
  
- \*\*The Area under the ROC curve is 73.5 which is somewhat satisfactory. \*\*
  
  
- \*\* Overall model could be improved with more data.\*\*

In [17]:

```
'''import pickle
filename = 'LR_model.sav'
pickle.dump(logreg, open(filename, 'wb'))'''
```

Out[17]:

```
"import pickle\nfilename = 'LR_model.sav'\npickle.dump(logreg, open(filename, 'wb'))"
```

In [ ]:

```

```
