# Supplementary material for: A new smart healthcare framework for real-time heart disease detection based on deep and machine learning
Source: PeerJ Comput Sci. 2021 Jul 28;7:e646. doi: 10.7717/peerj-cs.646 (PMC8330430; doi:10.7717/peerj-cs.646)
Supplement: Supplemental Information 5 — Coronary heart disease is often caused by the buildup of plaque, a waxy substance, inside the lining of larger coronary arteries. This unique service which predict coronary heart disease for user using Deep Learning module. User insert some data to module using editTexts in page and click on button finally result will be displayed. [file peerj-cs-07-646-s005.html]

Deep Learning Model


In [4]:

```
# low level
from enum import Enum
import os
import random
from time import time
import datetime
import math
 
# middle level
import numpy as np
import pandas as pd
import matplotlib.pyplot as plt
import matplotlib.image as mpimg
from PIL import Image
import pandas as pd
import sklearn
 
# frameworkscompute the correlation coefficient between multiple variables python
import torch 
from torch import nn, optim
from torch.nn import functional as F
from torch.utils.data import Dataset, DataLoader
from torch.utils.data.sampler import SubsetRandomSampler
from torchvision import transforms
```

# Data¶

In [5]:

```
data = pd.read_csv("framingham.csv")
data = data.dropna()
```

In [6]:

```
data
```

Out[6]:

|  | male | age | education | currentSmoker | cigsPerDay | BPMeds | prevalentStroke | prevalentHyp | diabetes | totChol | sysBP | diaBP | BMI | heartRate | glucose | TenYearCHD |
| --- | --- | --- | --- | --- | --- | --- | --- | --- | --- | --- | --- | --- | --- | --- | --- | --- |
| 0 | 1 | 39 | 4.0 | 0 | 0.0 | 0.0 | 0 | 0 | 0 | 195.0 | 106.0 | 70.0 | 26.97 | 80.0 | 77.0 | 0 |
| 1 | 0 | 46 | 2.0 | 0 | 0.0 | 0.0 | 0 | 0 | 0 | 250.0 | 121.0 | 81.0 | 28.73 | 95.0 | 76.0 | 0 |
| 2 | 1 | 48 | 1.0 | 1 | 20.0 | 0.0 | 0 | 0 | 0 | 245.0 | 127.5 | 80.0 | 25.34 | 75.0 | 70.0 | 0 |
| 3 | 0 | 61 | 3.0 | 1 | 30.0 | 0.0 | 0 | 1 | 0 | 225.0 | 150.0 | 95.0 | 28.58 | 65.0 | 103.0 | 1 |
| 4 | 0 | 46 | 3.0 | 1 | 23.0 | 0.0 | 0 | 0 | 0 | 285.0 | 130.0 | 84.0 | 23.10 | 85.0 | 85.0 | 0 |
| ... | ... | ... | ... | ... | ... | ... | ... | ... | ... | ... | ... | ... | ... | ... | ... | ... |
| 4233 | 1 | 50 | 1.0 | 1 | 1.0 | 0.0 | 0 | 1 | 0 | 313.0 | 179.0 | 92.0 | 25.97 | 66.0 | 86.0 | 1 |
| 4234 | 1 | 51 | 3.0 | 1 | 43.0 | 0.0 | 0 | 0 | 0 | 207.0 | 126.5 | 80.0 | 19.71 | 65.0 | 68.0 | 0 |
| 4237 | 0 | 52 | 2.0 | 0 | 0.0 | 0.0 | 0 | 0 | 0 | 269.0 | 133.5 | 83.0 | 21.47 | 80.0 | 107.0 | 0 |
| 4238 | 1 | 40 | 3.0 | 0 | 0.0 | 0.0 | 0 | 1 | 0 | 185.0 | 141.0 | 98.0 | 25.60 | 67.0 | 72.0 | 0 |
| 4239 | 0 | 39 | 3.0 | 1 | 30.0 | 0.0 | 0 | 0 | 0 | 196.0 | 133.0 | 86.0 | 20.91 | 85.0 | 80.0 | 0 |

3658 rows × 16 columns

## Normalizations¶

In [7]:

```
to_dummy_data = data.copy()
```

In [8]:

```
for d in ["age", "education", "cigsPerDay", "totChol", "sysBP", "diaBP", "BMI", "heartRate", "glucose"]:
    to_dummy_data.pop(d)
```

In [9]:

```
to_dummy_data
```

Out[9]:

|  | male | currentSmoker | BPMeds | prevalentStroke | prevalentHyp | diabetes | TenYearCHD |
| --- | --- | --- | --- | --- | --- | --- | --- |
| 0 | 1 | 0 | 0.0 | 0 | 0 | 0 | 0 |
| 1 | 0 | 0 | 0.0 | 0 | 0 | 0 | 0 |
| 2 | 1 | 1 | 0.0 | 0 | 0 | 0 | 0 |
| 3 | 0 | 1 | 0.0 | 0 | 1 | 0 | 1 |
| 4 | 0 | 1 | 0.0 | 0 | 0 | 0 | 0 |
| ... | ... | ... | ... | ... | ... | ... | ... |
| 4233 | 1 | 1 | 0.0 | 0 | 1 | 0 | 1 |
| 4234 | 1 | 1 | 0.0 | 0 | 0 | 0 | 0 |
| 4237 | 0 | 0 | 0.0 | 0 | 0 | 0 | 0 |
| 4238 | 1 | 0 | 0.0 | 0 | 1 | 0 | 0 |
| 4239 | 0 | 1 | 0.0 | 0 | 0 | 0 | 0 |

3658 rows × 7 columns

In [10]:

```
to_normalize_data = data.copy()
```

In [11]:

```
for d in ["male", "currentSmoker", "BPMeds", "prevalentStroke", "prevalentHyp", "diabetes", "TenYearCHD"]:
    to_normalize_data.pop(d)
```

In [12]:

```
to_normalize_data
```

Out[12]:

|  | age | education | cigsPerDay | totChol | sysBP | diaBP | BMI | heartRate | glucose |
| --- | --- | --- | --- | --- | --- | --- | --- | --- | --- |
| 0 | 39 | 4.0 | 0.0 | 195.0 | 106.0 | 70.0 | 26.97 | 80.0 | 77.0 |
| 1 | 46 | 2.0 | 0.0 | 250.0 | 121.0 | 81.0 | 28.73 | 95.0 | 76.0 |
| 2 | 48 | 1.0 | 20.0 | 245.0 | 127.5 | 80.0 | 25.34 | 75.0 | 70.0 |
| 3 | 61 | 3.0 | 30.0 | 225.0 | 150.0 | 95.0 | 28.58 | 65.0 | 103.0 |
| 4 | 46 | 3.0 | 23.0 | 285.0 | 130.0 | 84.0 | 23.10 | 85.0 | 85.0 |
| ... | ... | ... | ... | ... | ... | ... | ... | ... | ... |
| 4233 | 50 | 1.0 | 1.0 | 313.0 | 179.0 | 92.0 | 25.97 | 66.0 | 86.0 |
| 4234 | 51 | 3.0 | 43.0 | 207.0 | 126.5 | 80.0 | 19.71 | 65.0 | 68.0 |
| 4237 | 52 | 2.0 | 0.0 | 269.0 | 133.5 | 83.0 | 21.47 | 80.0 | 107.0 |
| 4238 | 40 | 3.0 | 0.0 | 185.0 | 141.0 | 98.0 | 25.60 | 67.0 | 72.0 |
| 4239 | 39 | 3.0 | 30.0 | 196.0 | 133.0 | 86.0 | 20.91 | 85.0 | 80.0 |

3658 rows × 9 columns

In [13]:

```
to_normalize_data_stats = to_normalize_data.describe()
to_normalize_data_stats = to_normalize_data_stats.transpose()
to_normalize_data_stats
```

Out[13]:

|  | count | mean | std | min | 25% | 50% | 75% | max |
| --- | --- | --- | --- | --- | --- | --- | --- | --- |
| age | 3658.0 | 49.551941 | 8.562029 | 32.00 | 42.00 | 49.00 | 56.0000 | 70.0 |
| education | 3658.0 | 1.980317 | 1.022656 | 1.00 | 1.00 | 2.00 | 3.0000 | 4.0 |
| cigsPerDay | 3658.0 | 9.025424 | 11.921590 | 0.00 | 0.00 | 0.00 | 20.0000 | 70.0 |
| totChol | 3658.0 | 236.847731 | 44.097681 | 113.00 | 206.00 | 234.00 | 263.0000 | 600.0 |
| sysBP | 3658.0 | 132.370558 | 22.086866 | 83.50 | 117.00 | 128.00 | 143.8750 | 295.0 |
| diaBP | 3658.0 | 82.917031 | 11.974258 | 48.00 | 75.00 | 82.00 | 90.0000 | 142.5 |
| BMI | 3658.0 | 25.782802 | 4.065601 | 15.54 | 23.08 | 25.38 | 28.0375 | 56.8 |
| heartRate | 3658.0 | 75.730727 | 11.981525 | 44.00 | 68.00 | 75.00 | 82.0000 | 143.0 |
| glucose | 3658.0 | 81.852925 | 23.904164 | 40.00 | 71.00 | 78.00 | 87.0000 | 394.0 |

In [14]:

```
def norm(x):
  return (x - to_normalize_data_stats['mean']) / to_normalize_data_stats['std']
normalize_data = norm(to_normalize_data)
```

In [15]:

```
normalize_data
```

Out[15]:

|  | age | education | cigsPerDay | totChol | sysBP | diaBP | BMI | heartRate | glucose |
| --- | --- | --- | --- | --- | --- | --- | --- | --- | --- |
| 0 | -1.232411 | 1.974939 | -0.757065 | -0.948978 | -1.193947 | -1.078733 | 0.292010 | 0.356321 | -0.203016 |
| 1 | -0.414848 | 0.019247 | -0.757065 | 0.298253 | -0.514811 | -0.160096 | 0.724911 | 1.608249 | -0.244850 |
| 2 | -0.181259 | -0.958599 | 0.920563 | 0.184868 | -0.220518 | -0.243609 | -0.108914 | -0.060988 | -0.495852 |
| 3 | 1.337073 | 0.997093 | 1.759377 | -0.268670 | 0.798187 | 1.009079 | 0.688016 | -0.895606 | 0.884661 |
| 4 | -0.414848 | 0.997093 | 1.172207 | 1.091946 | -0.107329 | 0.090441 | -0.659878 | 0.773630 | 0.131654 |
| ... | ... | ... | ... | ... | ... | ... | ... | ... | ... |
| 4233 | 0.052331 | -0.958599 | -0.673184 | 1.726900 | 2.111184 | 0.758541 | 0.046044 | -0.812144 | 0.173488 |
| 4234 | 0.169126 | 0.997093 | 2.849836 | -0.676855 | -0.265794 | -0.243609 | -1.493704 | -0.895606 | -0.579519 |
| 4237 | 0.285920 | 0.019247 | -0.757065 | 0.729115 | 0.051136 | 0.006929 | -1.060803 | 0.356321 | 1.051996 |
| 4238 | -1.115616 | 0.997093 | -0.757065 | -1.175747 | 0.390705 | 1.259616 | -0.044963 | -0.728682 | -0.412184 |
| 4239 | -1.232411 | 0.997093 | 1.759377 | -0.926301 | 0.028498 | 0.257466 | -1.198544 | 0.773630 | -0.077515 |

3658 rows × 9 columns

In [16]:

```
dummy_data = {}
for d in to_dummy_data:
    dummy_data[d] = pd.get_dummies(to_dummy_data[d])
```

In [17]:

```
for d in dummy_data:
    print(d,'\n',dummy_data[d][:10])
```

```
male 
    0  1
0  0  1
1  1  0
2  0  1
3  1  0
4  1  0
5  1  0
6  1  0
7  1  0
8  0  1
9  0  1
currentSmoker 
    0  1
0  1  0
1  1  0
2  0  1
3  0  1
4  0  1
5  1  0
6  1  0
7  0  1
8  1  0
9  0  1
BPMeds 
    0.0  1.0
0    1    0
1    1    0
2    1    0
3    1    0
4    1    0
5    1    0
6    1    0
7    1    0
8    1    0
9    1    0
prevalentStroke 
    0  1
0  1  0
1  1  0
2  1  0
3  1  0
4  1  0
5  1  0
6  1  0
7  1  0
8  1  0
9  1  0
prevalentHyp 
    0  1
0  1  0
1  1  0
2  1  0
3  0  1
4  1  0
5  0  1
6  1  0
7  1  0
8  0  1
9  0  1
diabetes 
    0  1
0  1  0
1  1  0
2  1  0
3  1  0
4  1  0
5  1  0
6  1  0
7  1  0
8  1  0
9  1  0
TenYearCHD 
    0  1
0  1  0
1  1  0
2  1  0
3  0  1
4  1  0
5  1  0
6  0  1
7  1  0
8  1  0
9  1  0
```

## Reset Index¶

In [18]:

```
normalize_data = normalize_data.reset_index()
for d in dummy_data:
    dummy_data[d] = dummy_data[d].reset_index()
```

## Data Class¶

In [19]:

```
class DataClass(Dataset):
    def __init__(self, normalize_data, dummy_data, targed_features):
        self.normalize_data = normalize_data
        self.dummy_data = dummy_data
        self.targed_features = targed_features        
    def __len__(self):
        return len(self.normalize_data)
    
    def __getitem__(self, indx):
        inputs = []
        inputs_labels = []
        for d in self.normalize_data:
            if d not in self.targed_features:
                continue
            inputs.append(self.normalize_data[d][indx])
            inputs_labels.append(d)
        
        for d in self.dummy_data:
            if d not in self.targed_features:
                continue
            inputs.append(self.dummy_data[d][0][indx])
            inputs.append(self.dummy_data[d][1][indx])
            inputs_labels.append("not"+d)
            inputs_labels.append(d)
        outputs = [self.dummy_data["TenYearCHD"][0][indx], self.dummy_data["TenYearCHD"][1][indx]]
        return [inputs, outputs, inputs_labels]
```

In [20]:

```
targeted_features =['age','education','cigsPerDay','totChol','sysBP','diaBP','BMI','heartRate','glucose','male','currentSmoker','BPMeds','prevalentStroke','prevalentHyp','diabetes',]
```

In [21]:

```
d = DataClass(normalize_data,dummy_data, targeted_features)
```

In [22]:

```
d.__getitem__(14)
```

Out[22]:

```
[[-1.3492059314898386,
  0.019246831306134114,
  0.920563099449568,
  -0.35937787790841474,
  0.3454289290146149,
  0.5915163212132383,
  -1.0903191118876747,
  1.6082487197905315,
  -0.49585190372336974,
  1,
  0,
  0,
  1,
  1,
  0,
  1,
  0,
  0,
  1,
  1,
  0],
 [0, 1],
 ['age',
  'education',
  'cigsPerDay',
  'totChol',
  'sysBP',
  'diaBP',
  'BMI',
  'heartRate',
  'glucose',
  'notmale',
  'male',
  'notcurrentSmoker',
  'currentSmoker',
  'notBPMeds',
  'BPMeds',
  'notprevalentStroke',
  'prevalentStroke',
  'notprevalentHyp',
  'prevalentHyp',
  'notdiabetes',
  'diabetes']]
```

In [23]:

```
len(d) == len(dummy_data['male']) == len(normalize_data)
```

Out[23]:

```
True
```

## Feature Importance¶

In [24]:

```
data = DataClass(normalize_data,dummy_data,targeted_features)
inputs = []
outputs = []
for i in range(len(data)):
    input, output, input_labels = data.__getitem__(i)
    inputs.append(input)
    outputs.append(output)
    print(f"\r{int(((i+1)/len(data)) *100)} % {i}",end='')
```

```
100 % 3657
```

In [25]:

```
inputs = np.asarray(inputs)
outputs = np.asarray(outputs)
```

### LinearRegression¶

In [26]:

```
# linear regression feature importance
from sklearn.datasets import make_regression
from sklearn.linear_model import LinearRegression
from matplotlib import pyplot

# define dataset
X, y = np.copy(inputs), np.copy(outputs)
print("done")
# define the model
model = LinearRegression()
# fit the model
model.fit(X, y)
# get importance
importance = model.coef_
# summarize feature importance
importance_list = []
for i,v in enumerate(importance[0]):
	importance_list.append([i,v])
	print('Feature: %0d, Score: %.5f' % (i,v))
# plot feature importance
pyplot.bar([x for x in range(len(importance[0]))], importance[0])

pyplot.show()
```

```
done
Feature: 0, Score: -0.06043
Feature: 1, Score: 0.00574
Feature: 2, Score: -0.02579
Feature: 3, Score: -0.00570
Feature: 4, Score: -0.05463
Feature: 5, Score: 0.01405
Feature: 6, Score: -0.00032
Feature: 7, Score: 0.00430
Feature: 8, Score: -0.02735
Feature: 9, Score: 0.02871
Feature: 10, Score: -0.02871
Feature: 11, Score: 0.00405
Feature: 12, Score: -0.00405
Feature: 13, Score: 0.02295
Feature: 14, Score: -0.02295
Feature: 15, Score: 0.06842
Feature: 16, Score: -0.06842
Feature: 17, Score: 0.01415
Feature: 18, Score: -0.01415
Feature: 19, Score: 0.01291
Feature: 20, Score: -0.01291
```

In [27]:

```
importance_list.sort(key=lambda x: abs(x[1]))
```

In [28]:

```
x = []
# i = 0
for indx, score in importance_list[::-1]:
    if input_labels[indx][:3] != "not": 
        x.append(score)
    # i+=1
    # if i==11:
        # break
        print(indx, input_labels[indx], score)
```

```
16 prevalentStroke -0.06841967836762754
0 age -0.06042639981908759
4 sysBP -0.05463045245561907
10 male -0.028712306641171654
8 glucose -0.027349990142217284
2 cigsPerDay -0.025793166697435047
14 BPMeds -0.02295383369362662
18 prevalentHyp -0.014153943400747711
5 diaBP 0.014047173120159599
20 diabetes -0.01291175019746795
1 education 0.005743317456592498
3 totChol -0.005702426498800216
7 heartRate 0.004301560047801274
12 currentSmoker -0.0040535080286050375
6 BMI -0.00032117823529004237
```

In [29]:

```
pyplot.bar([x for x in range(len(x))], x)

pyplot.show()
```

## Data Creator¶

In [30]:

```
def create_datasets(normalize_data=normalize_data, dummy_data=dummy_data, targeted_features=targeted_features, batch_size=32, valid_size = 0.25):

    train_dataset = DataClass(normalize_data,dummy_data, targeted_features)
    valid_dataset = DataClass(normalize_data,dummy_data, targeted_features)

    train_size = len(train_dataset)

    indices = list(range(train_size))
    np.random.shuffle(indices)

    valid_split_size = int(valid_size * train_size)

    train_indices, valid_indices = indices[valid_split_size:], indices[:valid_split_size]

    train_sampler = SubsetRandomSampler(train_indices)
    valid_sampler = SubsetRandomSampler(valid_indices)
   
    
    train_loader = DataLoader(train_dataset, batch_size=batch_size,sampler=train_sampler)
    valid_loader = DataLoader(valid_dataset, batch_size=batch_size,sampler=valid_sampler)
    
    print(f"\nTrain set: {len(train_indices)} sample")
    print(f"Valid set: {len(valid_indices)} sample")


    return train_loader, valid_loader
```

In [31]:

```
train_loader, valid_loader = create_datasets()
```

```
Train set: 2744 sample
Valid set: 914 sample
```

# Model¶

In [32]:

```
class Model(nn.Module):
    def __init__(self, input_size=20, n=15):
        super(Model, self).__init__()
        self.fc1 = nn.Linear(input_size, n)
        self.fc2 = nn.Linear(n, n)
        self.fc3 = nn.Linear(n, n)
        self.fc4 = nn.Linear(n, 2)
        self.ReLU = nn.ReLU()
    
    def forward(self, x):
        x = self.fc1(x)
        x = self.ReLU(x)
        x = self.fc2(x)
        x = self.ReLU(x)
        x = self.fc3(x)
        x = self.ReLU(x)
        x = self.fc4(x)
        return x
```

In [33]:

```
model = Model(21)
model(torch.rand(1,21))
```

Out[33]:

```
tensor([[-0.1802, -0.1827]], grad_fn=<AddmmBackward>)
```

# Workers¶

## Early Stopper¶

In [34]:

```
class EarlyStopping:
    def __init__(self,patience=15, path='/content'):
        self.patience = patience
        self.path = path
        self.counter = 0
        self.best_score = None
        self.early_stop = False
        self.val_loss_min = np.Inf
    
    def __call__(self, val_loss, model, epoch):
        score = -val_loss
        if self.best_score is None:
            self.best_score = score
        if score < self.best_score:
            self.counter += 1
            print(f'\rEpoch {epoch} EarlyStopping counter: {self.counter} out of {self.patience}')
            if self.counter >= self.patience:
                self.early_stop = True
        else:
            print("\rEpoch {} loss reduced from {} to {}".format(epoch, -self.best_score, val_loss))
            self.best_score = score
            self.save_checkpoint(val_loss, model)
            self.counter = 0
    
    def save_checkpoint(self, val_loss, model):
        torch.save(model.state_dict(), self.path)
        self.val_loss_min = val_loss
```

## Training¶

In [35]:

```
x = None
def train(train_loader, model, optimizer, criterion):
    global x
    model.train()
    t = time()
    train_loss = 0
    for i, data in enumerate(train_loader):
        inputs, outputs, labels = data

        l=[]
        for inp in inputs:
            l.append(inp.double())
        inputs = torch.stack(l,1)
        
        l=[]
        for out in outputs: 
            l.append(out.double())
        outputs = torch.stack(l,1)
        inputs = inputs.float()
        outputs = outputs.float()
        
        optimizer.zero_grad()
        
       
        _output = model(inputs)
        loss = criterion(_output, outputs)
        loss.backward()
        optimizer.step()
        
        train_loss += 1/(i + 1) * (loss.data - train_loss)
        

        torch.cuda.empty_cache()
        
        delay = time() - t
        hms = str(datetime.timedelta(seconds=int(delay* (len(train_loader)-(i+1)))))

        print(f"\rTaining finished batches {i+1}/{len(train_loader)}   {int(((i+1)/len(train_loader))*100)}%   delay {int(delay)}s   time left {hms}   loss {loss.item()}",end='')
        
        t = time()
        
    return train_loss
```

## Valid¶

In [36]:

```
def validation(valid_loader, model, criterion):
    model.eval()
    loss, running_loss = 0.0, 0.0
    t = time()
    with torch.no_grad():
        for i, data in enumerate(valid_loader):
            inputs, outputs, labels = data

            l=[]
            for inp in inputs:
                l.append(inp.double())
            inputs = torch.stack(l,1)
            
            l=[]
            for out in outputs:
                l.append(out.double())
            outputs = torch.stack(l,1)
            
            inputs = inputs.float()
            outputs = outputs.float()

            _output = model(inputs)
            loss = criterion(_output, outputs)
            running_loss += loss.item()
         

            delay = time() - t
            hms = str(datetime.timedelta(seconds=int(delay * (len(valid_loader)-(i+1)))))
            print(f"\rValidation finished batches {i+1}/{len(valid_loader)}   {int(((i+1)/len(valid_loader))*100)}%   delay {int(delay)}s   time left {hms}   loss { loss.item()} ",end='')
            t = time()
    avg_loss = running_loss / (i+1)
    model.train()
    return avg_loss
```

## Worker¶

In [37]:

```
def worker(n_epochs, train_loader, valid_loader, model, optimizer, criterion, early_stop):
    
    all_train_loss, all_val_loss  = [], []
    for epoch in range(n_epochs):
        train_loss = train(train_loader, model, optimizer, criterion)
        val_loss = validation(valid_loader, model, criterion)
        early_stop(val_loss, model, epoch)
        all_train_loss.append(train_loss)
        all_val_loss.append(val_loss)

        if early_stop.early_stop:
            break    
            
    return all_train_loss, all_val_loss
```

# Start¶

In [38]:

```
# optimizer = optim.SGD(model.parameters(), 0.01)
# criterion = nn.MSELoss()
# train(train_loader,model,optimizer,criterion);
```

In [39]:

```
optimizer = optim.SGD(model.parameters(), 0.01)
criterion = nn.MSELoss()
validation(valid_loader,model,criterion);
```

```
Validation finished batches 29/29   100%   delay 0s   time left 0:00:00   loss 0.7040398120880127
```

In [40]:

```
es = EarlyStopping(10,"model.pt")
all_train_loss, all_val_loss = worker(10000, train_loader, valid_loader, model, optimizer, criterion,es)
```

```
Epoch 0 loss reduced from 0.17187014067995138 to 0.17187014067995138:00   loss 0.1578494906425476 
Epoch 1 loss reduced from 0.17187014067995138 to 0.12437771813109003:00   loss 0.10504702478647232 
Epoch 2 loss reduced from 0.12437771813109003 to 0.12259651746215491:00   loss 0.09787362068891525 
Epoch 3 loss reduced from 0.12259651746215491 to 0.12239506147031126:00   loss 0.09848381578922272 
Epoch 4 EarlyStopping counter: 1 out of 10 delay 0s   time left 0:00:00   loss 0.17265361547470093 
Epoch 5 EarlyStopping counter: 2 out of 10 delay 0s   time left 0:00:00   loss 0.21791400015354156 
Epoch 6 loss reduced from 0.12239506147031126 to 0.12195394933223724:00   loss 0.10132371634244919 
Epoch 7 EarlyStopping counter: 1 out of 10 delay 0s   time left 0:00:00   loss 0.1360958218574524  
Epoch 8 loss reduced from 0.12195394933223724 to 0.12151846662163734:00   loss 0.0980958566069603  
Epoch 9 EarlyStopping counter: 1 out of 10 delay 0s   time left 0:00:00   loss 0.13561813533306122 
Epoch 10 EarlyStopping counter: 2 out of 10delay 0s   time left 0:00:00   loss 0.20030832290649414 
Epoch 11 EarlyStopping counter: 3 out of 10delay 0s   time left 0:00:00   loss 0.17234808206558228 
Epoch 12 EarlyStopping counter: 4 out of 10delay 0s   time left 0:00:00   loss 0.13997845351696014 
Epoch 13 loss reduced from 0.12151846662163734 to 0.1206322255191104200   loss 0.1002550721168518  
Epoch 14 loss reduced from 0.12063222551911042 to 0.1204521905245452100   loss 0.10518863797187805 
Epoch 15 loss reduced from 0.12045219052454521 to 0.1195267404204812500   loss 0.055721137672662735 
Epoch 16 loss reduced from 0.11952674042048125 to 0.1194252626135431500   loss 0.06381804496049881 
Epoch 17 EarlyStopping counter: 1 out of 10delay 0s   time left 0:00:00   loss 0.17394661903381348 
Epoch 18 EarlyStopping counter: 2 out of 10delay 0s   time left 0:00:00   loss 0.10077020525932312 
Epoch 19 EarlyStopping counter: 3 out of 10delay 0s   time left 0:00:00   loss 0.13921502232551575 
Epoch 20 EarlyStopping counter: 4 out of 10delay 0s   time left 0:00:00   loss 0.20868077874183655 
Epoch 21 EarlyStopping counter: 5 out of 10delay 0s   time left 0:00:00   loss 0.13550324738025665 
Epoch 22 loss reduced from 0.11942526261354315 to 0.1181319791438250700   loss 0.060830261558294296 
Epoch 23 EarlyStopping counter: 1 out of 10delay 0s   time left 0:00:00   loss 0.10432088375091553 
Epoch 24 loss reduced from 0.11813197914382507 to 0.1177225864396013:00   loss 0.05759265646338463 
Epoch 25 EarlyStopping counter: 1 out of 10delay 0s   time left 0:00:00   loss 0.09420490264892578 
Epoch 26 loss reduced from 0.1177225864396013 to 0.11720586658037942:00   loss 0.05475360155105591 
Epoch 27 loss reduced from 0.11720586658037942 to 0.1172040878184910500   loss 0.06359968334436417 
Epoch 28 EarlyStopping counter: 1 out of 10delay 0s   time left 0:00:00   loss 0.29403170943260193 
Epoch 29 EarlyStopping counter: 2 out of 10delay 0s   time left 0:00:00   loss 0.13924618065357208 
Epoch 30 loss reduced from 0.11720408781849105 to 0.1161267615469365300   loss 0.029057307168841362 
Epoch 31 EarlyStopping counter: 1 out of 10delay 0s   time left 0:00:00   loss 0.12999072670936584 
Epoch 32 EarlyStopping counter: 2 out of 10delay 0s   time left 0:00:00   loss 0.29421302676200867 
Epoch 33 loss reduced from 0.11612676154693653 to 0.1158665087202499600   loss 0.0321711041033268  
Epoch 34 EarlyStopping counter: 1 out of 10delay 0s   time left 0:00:00   loss 0.18382877111434937 
Epoch 35 EarlyStopping counter: 2 out of 10delay 0s   time left 0:00:00   loss 0.10015826672315598 
Epoch 36 EarlyStopping counter: 3 out of 10delay 0s   time left 0:00:00   loss 0.0886777937412262  
Epoch 37 EarlyStopping counter: 4 out of 10delay 0s   time left 0:00:00   loss 0.09276650846004486 
Epoch 38 EarlyStopping counter: 5 out of 10delay 0s   time left 0:00:00   loss 0.11908981204032898 
Epoch 39 EarlyStopping counter: 6 out of 10delay 0s   time left 0:00:00   loss 0.2653452157974243 
Epoch 40 loss reduced from 0.11586650872024996 to 0.1156925953410822800   loss 0.06251540780067444 
Epoch 41 loss reduced from 0.11569259534108228 to 0.1155709724487929600   loss 0.06417503952980042 
Epoch 42 EarlyStopping counter: 1 out of 10delay 0s   time left 0:00:00   loss 0.13389715552330017 
Epoch 43 EarlyStopping counter: 2 out of 10delay 0s   time left 0:00:00   loss 0.12954643368721008 
Epoch 44 EarlyStopping counter: 3 out of 10delay 0s   time left 0:00:00   loss 0.1145554929971695 
Epoch 45 EarlyStopping counter: 4 out of 10delay 0s   time left 0:00:00   loss 0.1771407276391983  
Epoch 46 loss reduced from 0.11557097244879296 to 0.1146952699998329400   loss 0.03353329002857208 
Epoch 47 EarlyStopping counter: 1 out of 10delay 0s   time left 0:00:00   loss 0.07772193104028702 
Epoch 48 EarlyStopping counter: 2 out of 10delay 0s   time left 0:00:00   loss 0.12299394607543945 
Epoch 49 EarlyStopping counter: 3 out of 10delay 0s   time left 0:00:00   loss 0.07734134793281555 
Epoch 50 EarlyStopping counter: 4 out of 10delay 0s   time left 0:00:00   loss 0.11347424983978271 
Epoch 51 loss reduced from 0.11469526999983294 to 0.1144855499010661600   loss 0.036694612354040146 
Epoch 52 EarlyStopping counter: 1 out of 10delay 0s   time left 0:00:00   loss 0.0975470170378685  
Epoch 53 loss reduced from 0.11448554990106616 to 0.1144664394187516200   loss 0.032128021121025085 
Epoch 54 EarlyStopping counter: 1 out of 10delay 0s   time left 0:00:00   loss 0.14945657551288605 
Epoch 55 EarlyStopping counter: 2 out of 10delay 0s   time left 0:00:00   loss 0.058722276240587234 
Epoch 56 EarlyStopping counter: 3 out of 10delay 0s   time left 0:00:00   loss 0.16971631348133087 
Epoch 57 EarlyStopping counter: 4 out of 10delay 0s   time left 0:00:00   loss 0.09440155327320099 
Epoch 58 EarlyStopping counter: 5 out of 10delay 0s   time left 0:00:00   loss 0.12843073904514313 
Epoch 59 loss reduced from 0.11446643941875162 to 0.1143369148003644:00   loss 0.03316545486450195 
Epoch 60 EarlyStopping counter: 1 out of 10delay 0s   time left 0:00:00   loss 0.05340813845396042 
Epoch 61 EarlyStopping counter: 2 out of 10delay 0s   time left 0:00:00   loss 0.08137177675962448 
Epoch 62 EarlyStopping counter: 3 out of 10delay 0s   time left 0:00:00   loss 0.08578494936227798 
Epoch 63 loss reduced from 0.1143369148003644 to 0.11420265996250613:00   loss 0.027384229004383087 
Epoch 64 EarlyStopping counter: 1 out of 10delay 0s   time left 0:00:00   loss 0.10893986374139786 
Epoch 65 EarlyStopping counter: 2 out of 10delay 0s   time left 0:00:00   loss 0.06369736790657043 
Epoch 66 EarlyStopping counter: 3 out of 10delay 0s   time left 0:00:00   loss 0.08266186714172363 
Epoch 67 EarlyStopping counter: 4 out of 10delay 0s   time left 0:00:00   loss 0.10450325161218643 
Epoch 68 EarlyStopping counter: 5 out of 10delay 0s   time left 0:00:00   loss 0.11989137530326843 
Epoch 69 EarlyStopping counter: 6 out of 10delay 0s   time left 0:00:00   loss 0.11246158182621002 
Epoch 70 EarlyStopping counter: 7 out of 10delay 0s   time left 0:00:00   loss 0.05497648939490318 
Epoch 71 EarlyStopping counter: 8 out of 10delay 0s   time left 0:00:00   loss 0.15880447626113892 
Epoch 72 EarlyStopping counter: 9 out of 10delay 0s   time left 0:00:00   loss 0.19269604980945587 
Epoch 73 EarlyStopping counter: 10 out of 10elay 0s   time left 0:00:00   loss 0.06451895087957382
```

In [41]:

```
def display_graph(train_losses, valid_losses):
    plt.plot(train_losses, label='Model pridictions')
    plt.plot(valid_losses, label='Real Valuse loss')

    plt.legend(frameon=False)
```

In [42]:

```
display_graph(all_train_loss, all_val_loss)
```

In [43]:

```
'''model.load_state_dict(torch.load("/content/model.pt"))'''
```

Out[43]:

```
'model.load_state_dict(torch.load("/content/model.pt"))'
```

In [44]:

```
'''model.eval()
example = torch.rand(1, 21)
traced_script_module = torch.jit.trace(model, example)
traced_script_module.save("/content/andoirdmodel.pt")'''
```

Out[44]:

```
'model.eval()\nexample = torch.rand(1, 21)\ntraced_script_module = torch.jit.trace(model, example)\ntraced_script_module.save("/content/andoirdmodel.pt")'
```

In [ ]:

```

```
