# Supplementary material for: A new smart healthcare framework for real-time heart disease detection based on deep and machine learning
Source: PeerJ Comput Sci. 2021 Jul 28;7:e646. doi: 10.7717/peerj-cs.646 (PMC8330430; doi:10.7717/peerj-cs.646)
Supplement: Supplemental Information 8 — Description of our system SHDML. We used python programming language for developing our desktop application: 1. Code is easy to read, use and maintain The effectiveness of the application greatly depends on the quality of its source code. 2. Supports multiple programming paradigms Python is constructed with the aim to help developers write logical and clean code for both large-scale and small-scale projects. Procedural programming, Object-oriented programming, Functional programming 3. Compatible with Major Platforms and Systems Python supports all the major operating systems and architectures. Being an interpreted language, Python offers the following benefits over compiled programming languages such as C, C++, Java, etc: It is easier to run the same Python program on multiple platforms, including Windows, Linux, macOS, etc. Since Python code is executed line-by-line, instead of all at once, it is easy to make alterations in the code and run the modified code and see the impact of changes immediately in the result. [file peerj-cs-07-646-s008.pdf]

5.1 General overview of application

To get the expected functionality of the project, the application has been programmed by dividing it into Two platforms. ATmega32 is the one which is collect heart beat from sensor and send BPM into android app using Bluetooth communication , the other side android app that receive BPM and path it to Machine learning module to detect heart disease

5.2 Embedded system Programming

5.2.1 Heart Beat Algorithm

First off, it's important to have a regular sample rate with high enough resolution to get reliable measurement of the timing between each beat. To do this, we set up Timer0, an 8-bit hardware timer on the ATmega32, so that it throws an interrupt every other millisecond. That gives us a sample rate of 500Hz, and beat-to-beat timing resolution of 2mS.

```
void Timer0_CtcInit(uint8 tcnt0V,uint8 ocr0V,uint16 n){
// load value into TCNT0
TCNT0 = tcnt0V;
// configure timer0 CTC mode
SET_BIT(TCCR0,_WGM01);
switch (n) {
  case 0:
   TCCR0 = (1<<_CS00);
  break;
   case 8:
   TCCR0 = (1<<_CS01);
  break;
   case 64:
         TCCR0 = (1<<_CS00) | (1<<_CS01);
   break;
   case 256:
         TCCR0 = (1<<_CS02);
    break;
    case 1024:
     TCCR0 = (1<<_CS00) | (1<<_CS02);
     break;
}          // load value to be compared with TCNT0 ..
           OCR0 = ocr0V;
           // enable interrupt for timer0
           SET_BIT(TIMSK,_OCIE0);
}
Timer0_CtcInit(0,124,256);
EN_G();
```

The register settings above tell Timer0 to go into CTC mode, and to count up to 124 (0x7C) over and over and over again. A prescaler of 256 is used to get the timing right so that it takes 2 milliseconds to count to 124. An interrupt flag is set every time Timer0 reaches 124, and a special function called an Interrupt Service Routine (ISR) that we wrote is run at the very next possible moment, no matter what the rest of the program is doing.
EN_G()ensures that global interrupts are enabled. Timing is important.

So, when the ATmega32 is powered up and running with Pulse Sensor Amped plugged into analog pin 0, it constantly (every 2 mS) reads the sensor value and looks for the heart beat. Here's how that works:

```
ISR(TIMER0_COMP_vect){
 Signal = Read_Analog(ADC0);
 sampleCounter += 2
 N_cnt = sampleCounter - lastBeatTime;
```

This function is called every 2 milliseconds. First thing to do is to take an analog reading of the Pulse Sensor. Next, we increment the variable sampleCounter. The sampleCounter variable is what we use to keep track of time. The variable N will help avoid noise later.

Next, we keep track of the highest and lowest values of the PPG wave, to get an accurate measure of amplitude.

```
if(Signal < thresh && N_cnt > (IBI/5)*3){
        if (Signal < Trough){
        Trough = Signal;
        }
        }
        if(Signal > thresh && Signal > P_cnt){
        P_cnt = Signal;
        }
```

Variable P_cnt and Trough hold peak and trough values, respectively. The thresh variable is initialized at 512 (middle of analog range) and changes during run time to track a point at 50% of amplitude as we will see later. There is a time period of 3/5 IBI that must pass before Trough gets updated as a way to avoid noise and false readings from the dicrotic notch.

Now, let's check and see if we have a pulse.

```
if (N_cnt > 250){ // avoid high frequency noise
        if ( (Signal > thresh) && (Pulse == 0) && (N_cnt > (IBI/5)*3) ){
        Pulse = 1; // set the Pulse flag when we think there is a pulse
        IBI = sampleCounter - lastBeatTime; // measure time between beats in mS
        lastBeatTime = sampleCounter; // keep track of time for next pulse
```

Before we even consider looking for a heart beat, a minimum amount of time has to pass. This helps avoid high frequency noise. 250 millisecond minimum N places an upper limit of 240 BPM. If you expect to have a higher BPM, adjust this accordingly and see a doctor. When the waveform rises past the thresh value, and 3/5 of the last IBI has

passed, we have a pulse Then we calculate the time since the last beat to get IBI, and update the lastBeatTime
The next bit is used to make sure we begin with a realistic BPM value on startup.

```
if(secondBeat){ // if this is the second beat, if secondBeat == TRUE
        secondBeat = 0; // clear secondBeat flag
        for(i=0; i<=9; i++){ // seed the running total to get a realisitic BPM at startup
        rate[i] = IBI;
        }
        }

        if(firstBeat){ // if it's the first time we found a beat, if firstBeat == TRUE
        firstBeat = 0; // clear firstBeat flag
        secondBeat = 1; // set the second beat flag

        return; // IBI value is unreliable so discard it
        }
```

The boolean firstBeat is initialized as true and secondBeat is initialized as false on start up, so the very first time we find a beat and get this far in the ISR, we get kicked out by the return; in the firstBeat conditional. That will end up throwing the first IBI reading away, cause it's lousy. The second time through, we can trust (more or less) the IBI, and use it to seed the rate[] array in order to start with a more accurate BPM. The BPM is derived from an average of the last 10 IBI values, hence the need to seed.

Let's calculate BPM!

```
runningTotal = 0; // clear the runningTotal variable

        for(i=0; i<=8; i++){ // shift data in the rate array
        rate[i] = rate[i+1]; // and drop the oldest IBI value
        runningTotal += rate[i]; // add up the 9 oldest IBI values
        }

        rate[9] = IBI; // add the latest IBI to the rate array
        runningTotal += rate[9]; // add the latest IBI to runningTotal
        runningTotal /= 10; // average the last 10 IBI values
        BPM = 60000/runningTotal; // how many beats can fit into a minute? that's
BPM!
        QS = 1; // set Quantified Self flag
        // QS FLAG IS NOT CLEARED INSIDE THIS ISR
        }
```

First, we grab a large variable, runningTotal, to collect the IBIs, then the contents of rate[] are shifted over and added to runnungTotal. The oldest IBI (11 beats ago) falls out of position 0, and the fresh IBI gets put into position 9. Then it's a simple process to average the array and calculate BPM. Last thing to do is to set the QS flag (short for

Quantified Self)so the rest of the program knows we have found the beat. That's it for the things to do when we find the beat.

```
if (Signal < thresh && Pulse == 1){
   Pulse = 0;
   amp = P - T;
   thresh = amp/2 + T;
   P = thresh;
   T = thresh;
 }
```

Pulse was declared true during the upward rise in Pulse Sensor signal when we found the beat, above, so when the signal crosses thresh going down, we can figure that the pulse is over. Then the amplitude of the wave that just passed is measured, and thresh is updated with the new 50% mark. P and T are reset to the new thresh. The algorithm is now primed and ready to find the next beat.

There's one more question to ask before the ISR is done. What if there are no beats?

```
if (N > 2500){
   thresh = 512;
   P = 512;
   T = 512;
   firstBeat = 1;
   secondBeat = 0;
   lastBeatTime = sampleCounter;
 }
```

If there is no beat event for 2.5 seconds, variables used to find the heartbeat are reinitialized to the start up values. Sort of a soft soft-reset. That's the end of the ISR!

Main Function that handle algorithm:

```
int main(void){

        Pulse = 0;
        QS = 0;
        firstBeat = 1;
        secondBeat = 0;
        DIO_SetPinDirection(PD,Pin0,INFREE);
        DIO_SetPinDirection(PD,Pin1,OUT);

  LCD_Init(); //initialize LCD
        ADC_Int();
        USART_Init(51);
        Timer0_CtcInit(0,124,256);
        EN_G();


        while(1){
        if (QS == 1){
                LCD_PutChar_XY(1,1);
                LCD_IntegerToString(BPM);
        USART_TxChar(BPM);

        }


        }



        return 0;
}
```

5.2.2 Bluetooth module

Bluetooth driver is based on UART communication protocol

Some UART communication protocol functions implemented in project to handle Bluetooth communication

1_ USART_INIT:-

-function used to initialize the UART module

```
void USART_Init(unsigned long baud){
	/* Set baud rate */
		UBRRH = (uint8)(baud>>8);
		UBRRL = (uint8)baud;
		/* Enable receiver and transmitter */
		UCSRB = (1<<RXEN)|(1<<TXEN);
		/* Set frame format: set select register  , 2stop bit,8data */
		UCSRC = (1<<URSEL)|(1<<USBS)|(3<<UCSZ0);
}
```

2_ USART_RxChar() :-

- Function used to receive data serially

```
char USART_RxChar()
{
	while ( !(UCSRA & (1<<RXC)) );
		/* Get and return received data from buffer */
		return UDR;
}
```

3_ USART_TxChar :-

-function used to transfer data serially

```
void USART_TxChar(char data)
{
	/* Wait for empty transmit buffer */
		while ( !( UCSRA & (1<<UDRE)) );
		/* Put data into buffer, sends the data */
		UDR = data;
}
```

5.2.3 LCD Configuration

Some function for LCD driver implemented in project

```
LCD_STATUS LCD_Init(){

	LCD_STATUS status = _NOK;
// set direction output for [ CONTROL PINS & DATA PINS ]
	DIO_SetPinDirection(LCD_CONTROL_PORT,LCD_RS,OUT);
	DIO_SetPinDirection(LCD_CONTROL_PORT,LCD_RW,OUT);
	DIO_SetPinDirection(LCD_CONTROL_PORT,LCD_E,OUT);
	// Set Direction for Data port
	DIO_SetPortDirection(LCD_DATA_PORT,0xff);
	_delay_ms(40);
	LCD_SendCmd(CMD_FUNCTION_SET_CMD);
	_delay_us(40);
	LCD_SendCmd(CMD_DISPLAY_ON_CURSOR_OFF);
	_delay_us(40);
	LCD_SendCmd(CMD_DISPLAY_CLEAR);
	_delay_ms(2);
	LCD_SendCmd(CMD_ENTRY_MODE_SET);

// end of initialization.....


	return status;


}
LCD_STATUS LCD_SendCmd(uint8 Cmd){
	LCD_STATUS status = 0;
	//RS = 0 (register command)
	//RW = 0 (Write on LCD)
	//send argument into LCD_DATA_PORT
	// Latching + delay....
	// E = HIGH
	//delay
	//E  = LOW
	// status  = _OK
	if(sizeof(Cmd) == sizeof(uint8)){
		DIO_WritePin(LCD_CONTROL_PORT,LCD_RS,LOW);
		DIO_WritePin(LCD_CONTROL_PORT,LCD_RW,LOW);
		DIO_WritePort(LCD_DATA_PORT,Cmd);
```

```c
			DIO_WritePin(LCD_CONTROL_PORT,LCD_E,HIGH);
			_delay_ms(1);
			DIO_WritePin(LCD_CONTROL_PORT,LCD_E,LOW);

status = _OK;
		}else {
			status = _NOK;
		}
			return status;
	}

void LCD_IntegerToString(uint16 data)
{
		char SHOWA [3];
		itoa(data,SHOWA,10);
		LCD_send_a_string(SHOWA);
	}
```

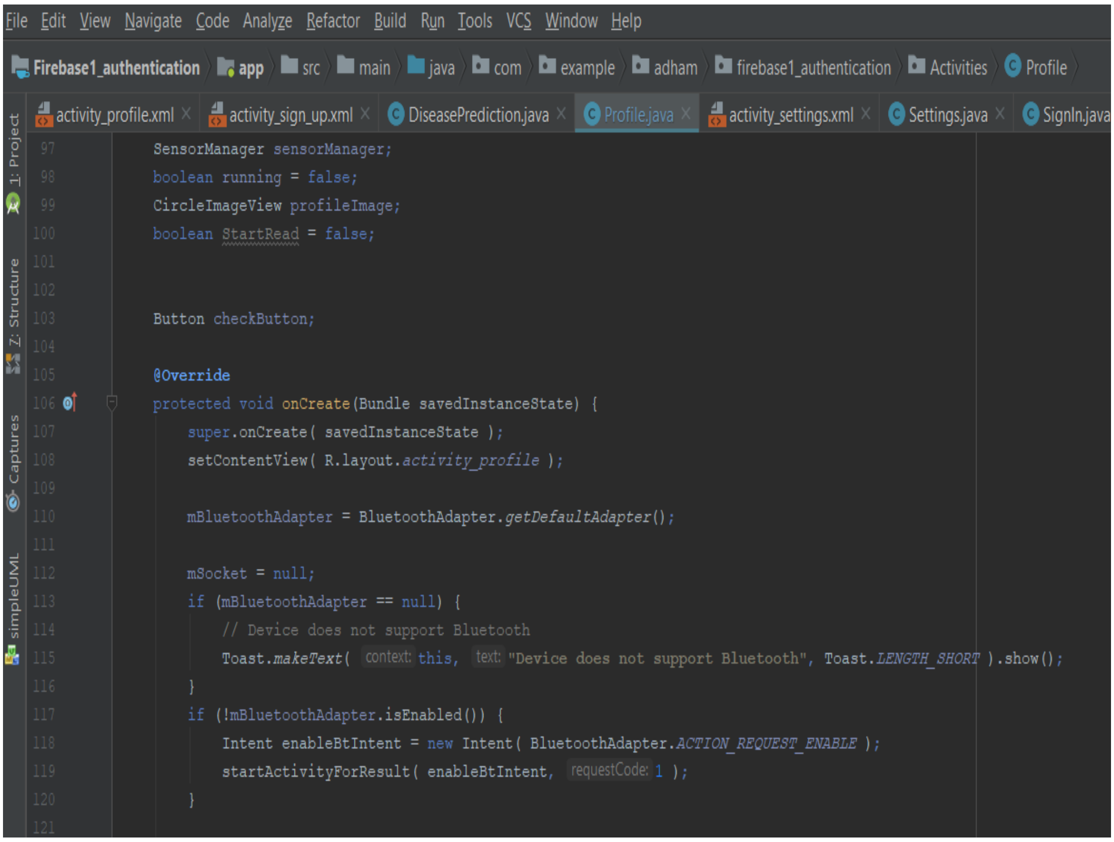

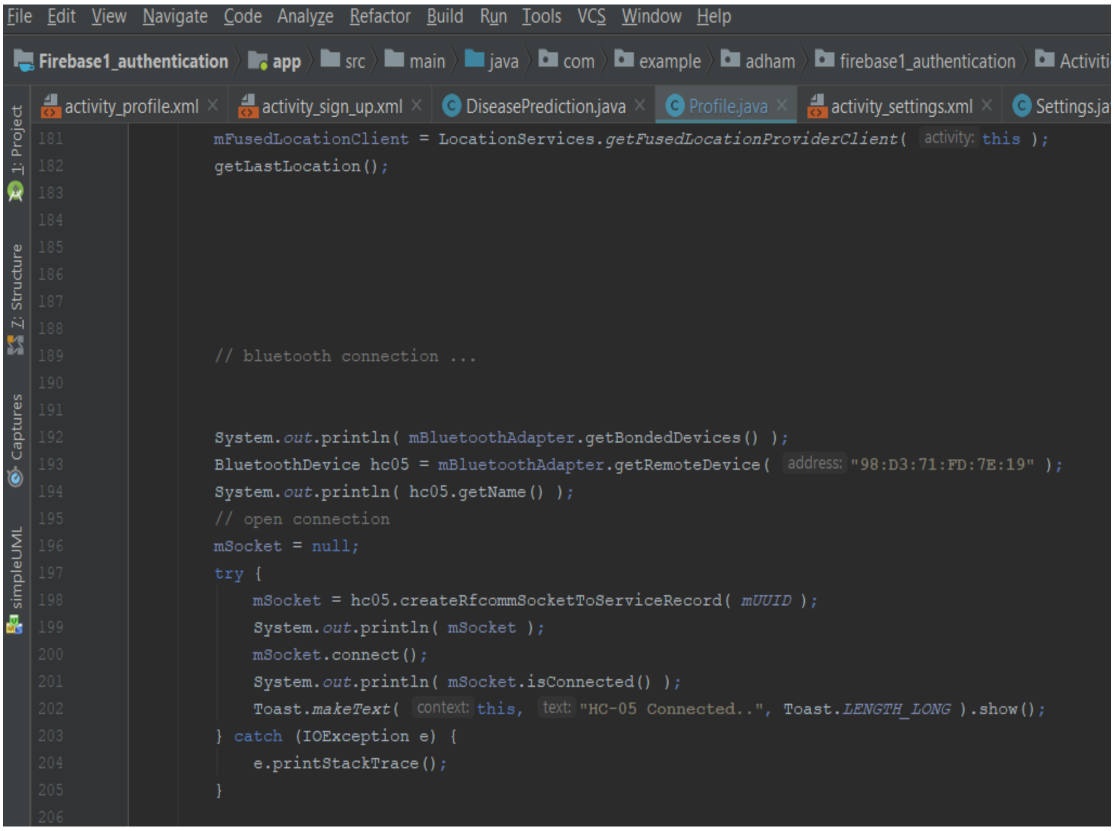

Source code for Data transfer using Thread run on background to not interrupt any Main Thread 'UI' functionality

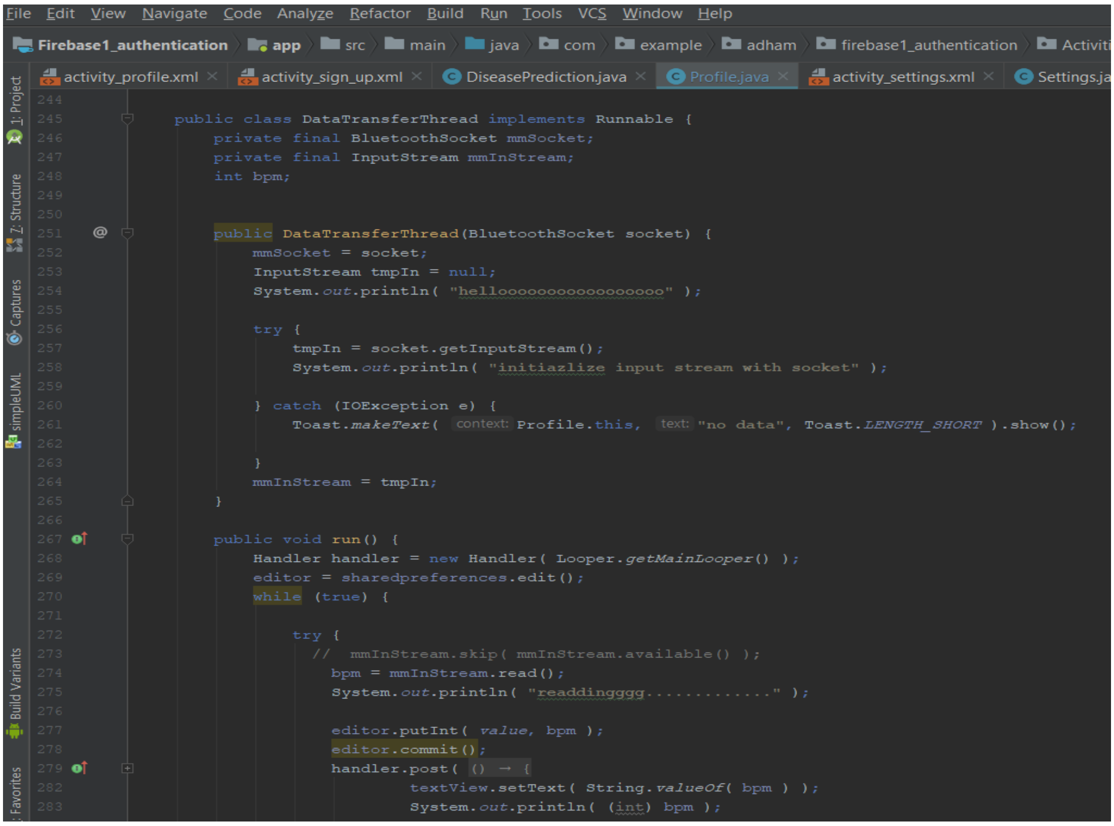

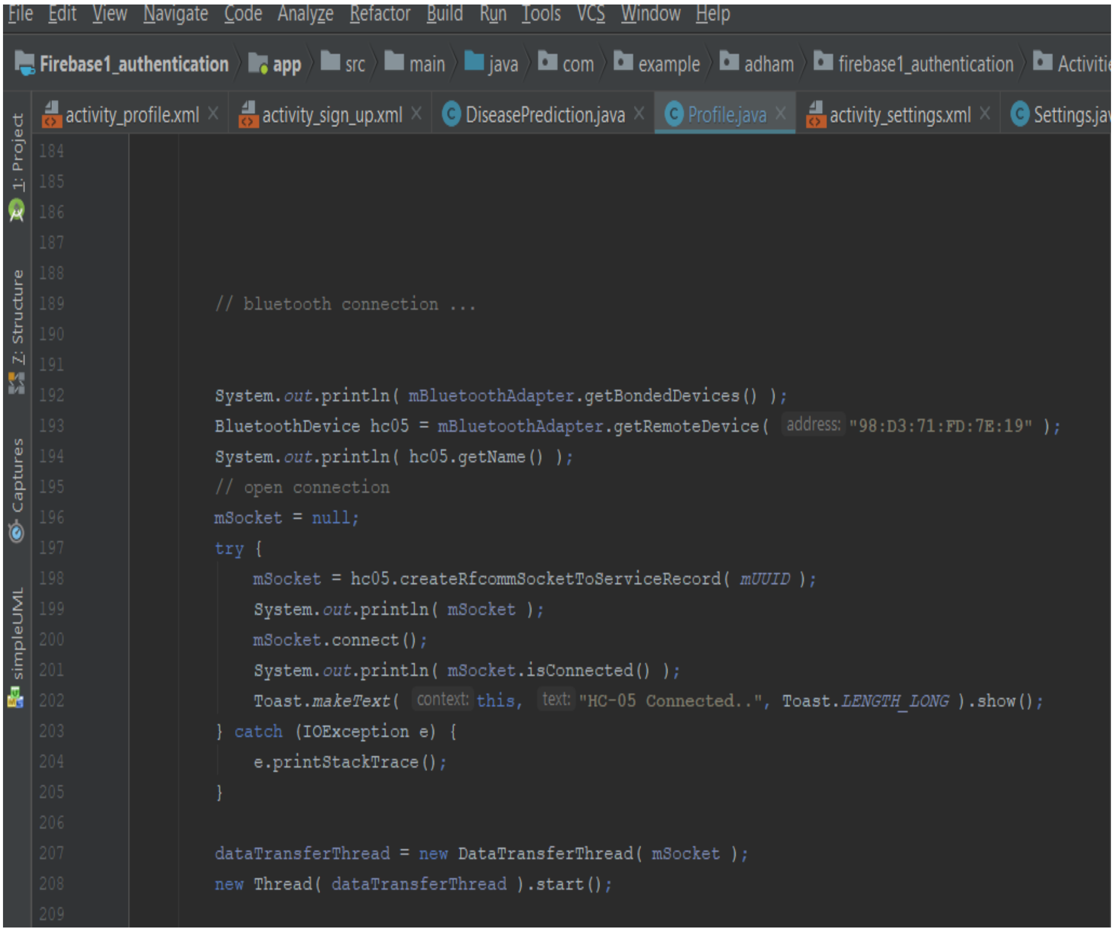

SMS service

Android platform provide API for handle SMS message transfer using SmsManager.SmsManager Manages SMS operations such as sending data, text, and SMS messages.

To use this library. First add some permissions in manifest file

```
<uses-permission android:name="android.permission.SEND_SMS"/>
<uses-permission android:name="android.permission.RECEIVE_SMS" />
<uses-permission android:name="android.permission.READ_SMS" />
```

Then use SmsManager class to handle SMS messages, in our project SMS Messages is handled also using own background thread to improve performance. Message will be sent when heart rate is not stable

```
public class SmsTransfer implements Runnable {


  @Override
  public void run() {
    while (true) {
      intent = new Intent( getApplicationContext(), Profile.class );
      pi = PendingIntent.getActivity( getApplicationContext(), 0, intent, 0 );
```

```
        //Get the SmsManager instance and call the sendTextMessage method to send message
        sms = SmsManager.getDefault();
        if (sharedpreferences.getInt( value, 0 ) > 185 || sharedpreferences.getInt( value, 0) < 50) {
            sms.sendTextMessage( "01003465433", null, "help me dad iam in "+sharedpreferences.getString(
longKey,null )+" .. "+sharedpreferences.getString( latKey,null ), null, null );
        }

        try {
            Thread.sleep( 10000 );
        } catch (InterruptedException e) {
            e.printStackTrace();
        }

      }
    }
}
```

Maps Service

One of the unique features of mobile application is location awareness. App users take their devices with them everywhere, and adding location awareness to our app offers users a more contextual experience. The location APIs available in Google Play services facilitate adding location awareness to our app with automated location tracking, geofencing, and activity recognition.

Some permissions are needed for foreground location when app requests either the `ACCESS_COARSE_LOCATION` permission or the `ACCESS_FINE_LOCATION` permission

```
<uses-permission android:name="android.permission.ACCESS_COARSE_LOCATION" />
<uses-permission android:name="android.permission.ACCESS_FINE_LOCATION" />
```

Create location services client

In your activity's onCreate() method, create an instance of the Fused Location Provider Client as the following code snippet shows.

```
FusedLocationProviderClient mFusedLocationClient;


@Override
   protected void onCreate(Bundle savedInstanceState) {
      super.onCreate( savedInstanceState );
      setContentView( R.layout.activity_profile );


      // handling location ...
      mFusedLocationClient = LocationServices.getFusedLocationProviderClient( this );
      getLastLocation();
```

Once I have created the Location Services client i can get the last known location of device. When app is connected to these can use the fused location

provider's `getLastLocation()` method to retrieve the device location. The precision of the location returned by this call is determined by the permission setting you put in app manifest

```java
@SuppressLint("MissingPermission")
private void getLastLocation () {
    if (checkPermissions()) {
        if (isLocationEnabled()) {
            mFusedLocationClient.getLastLocation().addOnCompleteListener(
                    new OnCompleteListener<Location>() {
                        @Override
                        public void onComplete(@NonNull Task<Location> task) {
                            Location location = task.getResult();
                            if (location == null) {
                                requestNewLocationData();
                            } else {
                                SharedPreferences.Editor editor = sharedpreferences.edit();
                                latitude = location.getLatitude();
                                longitude = location.getLongitude();
                                editor.putString( longKey,String.valueOf( longitude ) );
                                editor.putString( latKey,String.valueOf( latitude ));
                                editor.commit();
                            }
                        }
                    }
            );
        } else {
            Toast.makeText( context: this, text: "Turn on location", Toast.LENGTH_LONG ).show();
            Intent intent = new Intent( android.provider.Settings.ACTION_LOCATION_SOURCE_SETTINGS );
            startActivity( intent );
        }
    } else {
        requestPermissions();
    }
}
```

```java
@SuppressLint("MissingPermission")
private void requestNewLocationData () {

    LocationRequest mLocationRequest = new LocationRequest();
    mLocationRequest.setPriority( LocationRequest.PRIORITY_HIGH_ACCURACY );
    mLocationRequest.setInterval( 0 );
    mLocationRequest.setFastestInterval( 0 );
    mLocationRequest.setNumUpdates( 1 );

    mFusedLocationClient = LocationServices.getFusedLocationProviderClient( activity: this );
    mFusedLocationClient.requestLocationUpdates(
            mLocationRequest, mLocationCallback,
            Looper.myLooper()
    );

}

private LocationCallback mLocationCallback = new LocationCallback() {
    @Override
    public void onLocationResult(LocationResult locationResult) {
        Location mLastLocation = locationResult.getLastLocation();
    }
};



private boolean checkPermissions () {
    if (ActivityCompat.checkSelfPermission( context: this, Manifest.permission.ACCESS_COARSE_LOCATION ) == PackageManager.PERMISSION_GRANTED &&
            ActivityCompat.checkSelfPermission( context: this, Manifest.permission.ACCESS_FINE_LOCATION ) == PackageManager.PERMISSION_GRANTED )

    {
        return true;

    }
    return false;
}
```

Step Tracker Service

The Android sensor framework supports a wide variety of sensor types to measure the conditions of the physical environment and read the raw data from apps. The data from these sensors is delivered through the same SensorManager APIs as the built-in Android sensors.

App track user steps and continuously display it to user in Profile Page

```
sensorManager = (SensorManager) getSystemService( Context.SENSOR_SERVICE );

@Override
public void onSensorChanged(SensorEvent event) {



    stepCounts.setText( String.valueOf( event.values[0]));

}

@Override
public void onAccuracyChanged(Sensor sensor, int accuracy) {

}

@Override
public void onResume () {
  super.onResume();
  checkAuthenticationState();

  if (checkPermissions()) {
    getLastLocation();
  }
  running  = true;
  Sensor countsensor = sensorManager.getDefaultSensor( Sensor.TYPE_STEP_COUNTER );
  if(countsensor !=null){

    sensorManager.registerListener( this,countsensor,SensorManager.SENSOR_DELAY_UI );

  }
  else{
    Toast.makeText( this, "sensor not found..", Toast.LENGTH_SHORT ).show();

  }


}
```

Coronary hearts disease

Coronary heart disease is often caused by the buildup of plaque, a waxy substance, inside the lining of larger coronary arteries.

This unique service which predict coronary heart disease for user using Deep Learning module. User insert some data to module using editTexts in page and click on button finally result will be displayed.

```
public static String assetFilePath(Context context, String assetName) throws IOException {
    File file = new File(context.getFilesDir(), assetName);
    if (file.exists() && file.length() > 0) {
        return file.getAbsolutePath();
    }

    try (InputStream is = context.getAssets().open(assetName)) {
        try (OutputStream os = new FileOutputStream(file)) {
            byte[] buffer = new byte[4 * 1024];
            int read;
            while ((read = is.read(buffer)) != -1) {
                os.write(buffer, 0, read);
            }
            os.flush();
        }
        return file.getAbsolutePath();
    }
}

private String getPredictions(float age, float education, float cigsPerDay, float totChol,
                              float sysBP, float diaBP, float BMI, float heartRate, float glucose,
                              float male, float currentSmoker, float BPMeds, float prevalentStroke,
                              float prevalentHyp, float diabetes) {

    float notmale = male == 1 ? 0 : 1;
    float notcurrentSmoker = currentSmoker == 1 ? 0 : 1;
    float notBPMeds = BPMeds == 1 ? 0 : 1;
    float notprevalentStroke = prevalentStroke == 1 ? 0 : 1;
    float notprevalentHyp = prevalentHyp == 1 ? 0 : 1;
    float notdiabetes = diabetes == 1 ? 0 : 1;

    age = (float) ((age - 49.551941    ) / 8.562029);
    education = (float) ((education - 1.980317 ) / 1.022656);
    cigsPerDay = (float) ((cigsPerDay -9.025424 ) / 11.921590);
    totChol  = (float) ((totChol - 236.847731) / 44.097681);
    sysBP  = (float) ((sysBP - 132.370558) / 22.086866);
    diaBP  = (float) ((diaBP - 82.917031) / 11.974258);
    BMI   = (float) ((BMI - 25.782802) / 4.065601);
    heartRate = (float) ((heartRate- 75.730727) / 11.981525);
    glucose = (float) ((glucose - 81.852925) / 23.904164);


    float [] inputs = {age,education,cigsPerDay,totChol,sysBP,diaBP,BMI,heartRate,glucose,
            notmale,male,notcurrentSmoker,currentSmoker,notBPMeds,BPMeds,notprevalentStroke,
            prevalentStroke,notprevalentHyp,prevalentHyp,notdiabetes,diabetes};
    final long[] shape = new long[]{1, inputs.length};

    final Tensor inputTensor = Tensor.fromBlob(inputs, shape);
```

```
    Tensor outputTensor = module.forward(IValue.from(inputTensor)).toTensor();
    float[] scores = outputTensor.getDataAsFloatArray();
    if (scores[0]>scores[1])
        return "no TenYearCHD";
    return "yes TenYearCHD";
}
```

module added in android app as a file so Write operation into file is easy way to insert user data into module and get prediction using some libraries implemented in android

```
import org.pytorch.IValue;
import org.pytorch.Module;
import org.pytorch.Tensor;
```

Firebase Service

Firebase can power your app's backend, including data storage, user authentication, static hosting, and more. Focus on creating extraordinary user experiences.

Our app use firebase for many services

- **Real-time Database** – Firebase supports JSON data and all users connected to it receive live updates after every change.
- **Authentication** – We can use anonymous, password or different social authentications.
- **Hosting** – The applications can be deployed over secured connection to Firebase servers.

Add Firebase Authentication to your app

```
implementation 'com.google.firebase:firebase-auth:19.3.1'
```

Check current auth state

1. Declare an instance of FirebaseAuth.

```
private FirebaseAuth mAuth;
```

2. In the onCreate() method, initialize the FirebaseAuth instance.

```
// Initialize Firebase Auth
mAuth = FirebaseAuth.getInstance();
```

3. When initializing your Activity, check to see if the user is currently signed in.

```
@Override
public void onStart() {
    super.onStart();
    // Check if user is signed in (non-null) and update UI accordingly.
    FirebaseUser currentUser = mAuth.getCurrentUser();
    updateUI(currentUser);
}
```

Sign up new users

Create a new createAccount method that takes in an email address and password, validates them, and then creates a new user with the [createUserWithEmailAndPassword]

```
firebaseAuth.createUserWithEmailAndPassword( email, Pass ).addOnCompleteListener( new
OnCompleteListener<AuthResult>() {
    @Override
    public void onComplete(@NonNull Task<AuthResult> task) {
      progressBar.setVisibility( View.GONE );
      if (task.isSuccessful()) {
        // send Verification Email
        EmailVerification();
        // Save User Information -------------------------------------------------------------------
        User user = new User(  );
        user.setName( User_Name.getText().toString().trim() );
        user.setPhone( Phone_Number.getText().toString().trim() );
        user.setProfile_image( "" );
        user.setSecurity_level( "1" );
        user.setUser_id( FirebaseAuth.getInstance().getCurrentUser().getUid() );

        FirebaseDatabase.getInstance().getReference().child( getString(R.string.User_nodes) )
            .child( FirebaseAuth.getInstance().getCurrentUser().getUid() )
            .setValue( user )
            .addOnCompleteListener( new OnCompleteListener<Void>() {
          @Override
          public void onComplete(@NonNull Task<Void> task) {

            FirebaseAuth.getInstance().signOut();
            // redirected to login screen to login
            GoToSignIn();

          }
        } ).addOnFailureListener( new OnFailureListener() {
          @Override
          public void onFailure(@NonNull Exception e) {
            FirebaseAuth.getInstance().signOut();
            // redirected to login screen to login
            GoToSignIn();
            Toast.makeText( SignUp.this, "SomeThing Wrong ,Try again", Toast.LENGTH_SHORT ).show();
          }
        } );

        //------------------------------------------------------------------------------------------------------------


      }
      // check if the email is already registered in database :
      else if (task.getException() instanceof FirebaseAuthUserCollisionException) {
        Email.setError( "This Email is already registered" );
        Email.requestFocus();
        return;
      } else {
        Toast.makeText( SignUp.this, "Registeration Failed", SHORT ).show();
```

Add a form to register new users with their email and password and call this new method when it is submitted.

Sign in existing users

Create a new signIn method which takes in an email address and password, validates them, and then signs a user in with the signInWithEmailAndPassword method.

```
firebaseAuth.signInWithEmailAndPassword(email,Pass ).addOnCompleteListener( new
OnCompleteListener<AuthResult>() {
    @Override
    public void onComplete(@NonNull Task<AuthResult> task) {
        FirebaseUser user = firebaseAuth.getCurrentUser();
        progressBar.setVisibility( View.GONE );
        if(task.isSuccessful())
        {
            // When the user  attempts to sign into the app,
            // code can be added to check that the user's email address has been verified by calling the
isEmailVerified()
            // method of the FirebaseUser instance:
            if (user.isEmailVerified()) {
                OpenProfile();
            }
            else {
                Toast.makeText( com.example.adham.firebase1_authentication.Activities.SignIn.this, "Check Your Email
Inbox for a Verification link", Toast.LENGTH_SHORT).show();
                FirebaseAuth.getInstance().signOut();
            }

        }

    }

    } ).addOnFailureListener( new OnFailureListener() {
    @Override
    public void onFailure(@NonNull Exception e) {
        if (e instanceof FirebaseAuthInvalidCredentialsException) {
            Toast.makeText( SignIn.this, "Invalid password", Toast.LENGTH_SHORT ).show();
        } else if (e instanceof FirebaseAuthInvalidUserException) {
           // Toast.makeText( SignIn.this, "Incorrect email address", Toast.LENGTH_SHORT ).show();
          //  These error codes are provided in the form of string objects containing the error code name.
            //Testing for an error, therefore, simply involves performing string comparisons against the error code.
            String errorCode =((FirebaseAuthInvalidUserException) e).getErrorCode();
            if (errorCode.equals("ERROR_USER_NOT_FOUND")) {
                Toast.makeText( SignIn.this,  "No matching account found", Toast.LENGTH_SHORT ).show();
            } else if (errorCode.equals("ERROR_USER_DISABLED")) {
                Toast.makeText( SignIn.this, "User account has been disabled", Toast.LENGTH_SHORT ).show();
            }
        } else {
            Toast.makeText( SignIn.this, "Error", Toast.LENGTH_SHORT ).show();
```

Add the Realtime Database SDK to app

```
implementation 'com.google.firebase:firebase-database:19.3.0'
```

Configure Realtime Database Rules

The Realtime Database provides a declarative rules language that allows you to define how your data should be structured, how it should be indexed, and when your data can be read from and written to.

By default, read and write access to your database is restricted so only authenticated users can read or write data. To get started without setting up [Authentication](), you can [configure your rules for public access](). This does make your database open to anyone, even people not using your app, so be sure to restrict your database again when you set up authentication.

Write to database

Retrieve an instance of your database using `getInstance()` and reference the location you want to write to.

Snipptes codes from Setting page for Writing "updating" User data in firebase

```
DatabaseReference reference = FirebaseDatabase.getInstance().getReference();
      /*
      ------ Change Name -----
       */
      if(!UserName.getText().toString().equals("")){
         reference.child(getString(R.string.User_nodes))
               .child(FirebaseAuth.getInstance().getCurrentUser().getUid())
               .child(getString(R.string.name))
               .setValue(UserName.getText().toString());
      }


      /*
      ------ Change Phone Number -----
       */
      if(!PhoneNumber.getText().toString().equals("")){
         reference.child(getString(R.string.User_nodes))
               .child(FirebaseAuth.getInstance().getCurrentUser().getUid())
               .child(getString(R.string.phone))
               .setValue(PhoneNumber.getText().toString());
      }

      // Upload Image into Firebase Storage
      if(mSelectedImageUri != null){
         uploadNewPhoto(mSelectedImageUri);
      }else if(mSelectedImageBitmap  != null){
         uploadNewPhoto(mSelectedImageBitmap);
      }

      Toast.makeText(Settings.this, "saved", Toast.LENGTH_SHORT).show();
   }
});
```

Read from database

Snipptes codes from Setting page for reading User data in firebase into Setting page

```
private void getUserAccountsData(){
   Log.v("", "getUserAccountsData: getting the users account information");

   DatabaseReference reference = FirebaseDatabase.getInstance().getReference();

   /*
      --------- Query method 1 ----------
    */
   Query query1 = reference.child(getString(R.string.User_nodes))
         .orderByKey()
         .equalTo(FirebaseAuth.getInstance().getCurrentUser().getUid());

   query1.addListenerForSingleValueEvent(new ValueEventListener() {
      @Override
      public void onDataChange(DataSnapshot dataSnapshot) {

         for(DataSnapshot singleSnapshot : dataSnapshot.getChildren()){
            User user = singleSnapshot.getValue(User.class);
            Log.v("=============", "onDataChange: (QUERY METHOD 1) found user: " + user.toString());

            UserName.setText(user.getName());
            PhoneNumber.setText(user.getPhone());
            ImageLoader.getInstance().displayImage( user.getProfile_image(),profileImage);
         }
      }
```

Python Desktop app

We used python programming language for developing our desktop application due to

1. Code is easy to read, use and maintain

The effectiveness of the application greatly depends on the quality of its source code.

2. Supports multiple programming paradigms

Python is constructed with the aim to help developers write logical and clean code for both large-scale and small-scale projects.

Procedural programming, Object-oriented programming, Functional programming

3. Compatible with Major Platforms and Systems

Python supports all the major operating systems and architectures.

Being an interpreted language, Python offers the following benefits over compiled programming languages such as C, C++, Java, etc:

It is easier to run the same Python program on multiple platforms, including Windows, Linux, macOS, etc.

Since Python code is executed line-by-line, instead of all at once, it is easy to make alterations in the code and run the modified code and see the impact of changes immediately in the result.

4. Large standard library

Python has a robust and large standard library that makes it stand out from other programming languages. Its standard library contains a wide range of modules, operations and web service tools that you can select and use for your applications without writing additional code.

Importing Principals libraries

```
from tkinter import *
from tkinter import messagebox
from tkinter import font as tkFont
from operator import itemgetter
import numpy as np
import pickle
from firebase import firebase
import pyrebase
import os
from PIL import Image, ImageTk
from urllib.request import urlopen
from io import BytesIO
```

Tkinter for GUI interface objects.

Operator is a library for getting items data.

Numpy for dealing with numpy arrays.

Pickle for loading ML model.

Firebase and pyrebase for firebase cloud databse connection.

OS for dealing with windows function.

PIL for using images.

Urllib for dealing with url links from web.

IO for bytes inputs and outputs.

Initializing the Tkinter

```
if __name__ == "__main__":
    root = Tk()
    root.geometry("780x340")
    root.configure(bg='deepskyblue')
    app = MyApp(root)
    root.mainloop()
```

Whenever the Python interpreter reads a source file, it does two things:

1. it sets a few special variables like `__name__`, and then
2. it executes all of the code found in the file.

When the Python interpreter reads a source file, it first defines a few special variables. In this case, we care about the `__name__` variable, the interpreter will assign the hard-coded string `"__main__"` to the `__name__` variable, After the special variables are set up, the interpreter executes all the code in the module, one statement at a time.

So first, we start with if __name__ = “__main__” to make the interpreter to read these lines first then the other code, inside the if condition we declare the tkinter to root variable to start the window and settings the dimensions and background of the app then call MyApp class and pass the root variable as object. root.mainloop() is for continuous run of the window.

Defining the MyApp Class as the startup window

```
class MyApp(object):

    global firebaseConfig
    global fb
    global authen
    global db
    global st
    global user

    def __init__(self, parent):
        """Constructor"""
        self.root = parent
        self.root.title("Heart Disease Prediction")
        self.root.iconbitmap(r'heart-512.ico')

        self.root.bind('<Escape>', lambda e: self.root.destroy())

        self.frame = Frame(parent)
        self.frame.pack()
        self.frame.configure(bg='deepskyblue')
```

The first class is MyApp that receive the root object to config the window.

The MyApp class have some global variables that is used for defining the variables of firebase connection and can use these variables with other classes easily.

We make def __init__ with two parameters self and parent.

Self is used to instance the root properties and parent to assign these def as the parent of child classes, after that we define the constructor and set its new properties then start a new frame to organize the window components.

Setup Firebase Connection

```
#Firebase connection
MyApp.firebaseConfig = {

    "apiKey":"AIzaSyD7brf97i_NLnjba5DWw5Laae28wTYp-as",
    "authDomain": "authenticationapp-4b9db.firebaseapp.com",
    "databaseURL": "https://authenticationapp-4b9db.firebaseio.com",
    "projectId": "authenticationapp-4b9db",
    "storageBucket": "authenticationapp-4b9db.appspot.com",
    "messagingSenderId": "918890077991",
    "appId": "1:918890077991:web:e24e7ff29bc94ca7605302"

}

MyApp.fb = pyrebase.initialize_app(MyApp.firebaseConfig)
MyApp.db= MyApp.fb.database()
MyApp.st = MyApp.fb.storage()
MyApp.authen = MyApp.fb.auth()
```

Setting up the firebase connection to the global variables of MyApp class.

Firebaseconfig variable is a dictionary contains a script of connection with our firebase cloud database. Then we initialize this script with firebase library to start the connection.

Firebase has 3 main parameters (Authentication, Database, Storage), we declare all of them by using pyrebase and firebase libraries.

Creating Account and Login Processes Using Pyrebase

```
#creating account process
def create(self):
    if (passEntry.get() == cpassEntry.get()):
        if (len(nameEntry.get()) == 0 or len(mailEntry.get()) == 0 or len(passEntry.get()) == 0
           or len(cpassEntry.get()) == 0 or len(pnEntry.get()) == 0):
            messagebox.showerror('Data missed','Please, enter all data')
        else:
            try:
                user  = authen.create_user_with_email_and_password(mailEntry.get(),passEntry.get())
                authen.send_email_verification(user['idToken'])
                data =  { 'name':nameEntry.get(),'phone':pnEntry.get(), 'profile_image':'', 'security_level':'1'
                         , 'user_id':user['localId'] }
                db.child("User").child(user['localId']).set(data)
                messagebox.showinfo('Sign Up Successfull','Done :D\nPlease, Check your e-mail for verfication')
            except:
                messagebox.showinfo('Change the email','This email already exist')
    else:
        messagebox.showwarning('attention','The confirm password is not matched')

#login to account process
def login(self):
    if (len(LmailEntry.get()) == 0 and len(LpassEntry.get()) == 0):
        messagebox.showinfo('No info','Please, enter your info')
    else:
        try:
            email = LmailEntry.get()
            password = LpassEntry.get()
            MyApp.user  = MyApp.authen.sign_in_with_email_and_password(email,password)
            check = MyApp.authen.get_account_info(MyApp.user['idToken'])
            verify = check['users']
            valid = [y['emailVerified'] for y in verify]
            if (valid == [True]):
                self.hide()
                subFrame = mainFrame(self)
            else:
                messagebox.showinfo('Attention','This email is not verified\nPlease, Check you email to verify')
        except:
            messagebox.showerror('Failed','email or passwrod is invalid')
```

Creating account def use some conditions to guarantee that no problems happens during creating account. Check that the password and confirm password entries are the same inputs with outer if condition, then inner if condition to check that all entries has inputs, the else of the inner if condition has a try except to restrict to make a new account with an email that already has been used for another account.

Authen.create_user_with_email_and_passwrod() is a function of authentication attribute inside pyrebase library for passing the data to cloud firebase server.

Authen.send_email_verfication() is a function for sending verification email for the new account to be able to login.

Login to an existing account also has condition to guarantee that no problems happens in login process. Outer if condition for checking that the user have entered the email and password, the else condition has try except to deny the login process

to an un-verified account, if the account is verified the login process complete successful and open mainframe window.

Authen.sign_in_with_email_and_passwrod() is a function of authentication attribute inside pyrebase library for login to already existed account on cloud firebase server.

MyApp.authen.get_account_info(MyApp.user['idToken'])

By these function we have a dictionary that contain some info connected with user idToken such as email is verified or not, so we splitting this dictionary to get emailVerified attribute to know whether it is True of False.

Forgot Password and Login saved Data

```
#forgetpassword process
def forgetPass(self):
    if (len(LmailEntry.get()) == 0):
        messagebox.showwarning('attention','Please, Enter the email')
    else:
        try:
            authen.send_password_reset_email(LmailEntry.get())
            messagebox.showinfo('Info','Recovery email has been sent\nPlease, Check your email')
        except:
            messagebox.showinfo('Info','This email does not exist')

#login from saved data
def rem_login(self):
    hand = open("rem.txt","r")
    data = hand.read()
    data = data.split("\n")
    email = str(data[0])
    password = str(data[1])
    hand.close()
    MyApp.user = MyApp.authen.sign_in_with_email_and_password(email,password)
    self.hide()
    subFrame = mainFrame(self)

#save login data
def remmeber(self):
    if (rem.get() == 1):
        hand = open("rem.txt","w+")
        hand.write(LmailEntry.get()+"\n")
        hand.write(LpassEntry.get()+"\n")
        hand.close()
    else:
        hand = open("rem.txt","r+")
        hand.truncate(0)
        hand.close()
```

If the user forgot his password, we provide a reset password feature by forget pass def.

It contains Authen.send_password_reset_email () is a function of authentication attribute inside pyrebase library for sending an email for resetting the password of a existing account, so we did we try except to deny sending forgot password email to non-existing email in our database.

Also, there is an if condition to ensure that the user entered his email.

Another feature that called remember me as it saves the login info to auto login the user account if he didn’t log out but it needs the user to check the remember me checkbox.

MyApp Class Tasks rules

```
#show this window
def show(self):
    self.root.update()
    self.root.deiconify()

#hidding this window
def hide(self):
    self.root.withdraw()

#exit this window
def quit(self):
    self.root.destroy()
```

The MyApp class has some def that used for show, hide and exit tasks with some rules to each task.

Creating mainframe Class as the main app window

```
class mainFrame(Toplevel):

    global name
    global mail
    global phone
    global profImg

    def __init__(self, main):
        #Constructor
        self.main_frame = main
        Toplevel.__init__(self)
        self.geometry("750x300")
        self.title('Heart Disease Prediction')
        self.configure(bg='deepskyblue')
        self.iconbitmap(r'heart-512.ico')

        self.protocol("WM_DELETE_WINDOW", self.Exit)
        self.bind('<Escape>', lambda e: self.Exit())
```

The second class is the mainframe that contain the main app interface that send Toplevel object, also have global variables name, mail, phone and profImg, then building the constructer inside __init__ def that initializes the variables, windows dimensions and configurations then other properties. This window is child from the parent MyApp class.

Retrieve account data from firebase using Pyrebase

```
#get account data
info = MyApp.db.child("User").child(MyApp.user['localId']).get()
x = MyApp.authen.get_account_info(MyApp.user['idToken'])
y = x['users']
z = [i['email'] for i in y]
mainFrame.mail = z[0]
mainFrame.name = info[0].val()
mainFrame.phone = info[1].val()
mainFrame.profImg = info[2].val()
```

Here w get the user info name, phone number, image and email after the user login to his account to use his data in the app from the already defined variable that start the cloud firebase server connection.

MyApp.db.child("User").child(MyApp.user['localId']).get()

Child refers to access to tables in the firebase database, we called User table, then the localId attribute of the logged in user. By that we gain name, phone and image only.The data received in a list so we retrieve them with indexs.

x = MyApp.authen.get_account_info(MyApp.user['idToken'])

By these functions we have a dictionary that contain some info connected with user idToken such as the email, to get the email we have to split the dictionary to get the email.

CHD Prediction of user's inputs using Machine Learning model with logistic Regression

```
#prediction
def predict(self):
    model = pickle.load(open('LR_model.sav', 'rb'))
    try:
        record = [[int(genderEntry.get()),int(ageEntry.get()),int(smokerEntry.get()),int(cigEntry.get()),
                int(medicEntry.get()),int(strokeEntry.get()),int(hypEntry.get()),int(diabetesEntry.get()),
                int(cholEntry.get()),int(sysBPEntry.get()),int(diaBPEntry.get()),int(massEntry.get()),
                int(hrEntry.get()),int(glEntry.get())]]
        p = model.predict_proba(record)
        r = model.predict(record)
        print(record)
        print(p)
        print(r)
        if (p[0,1] > 0.30):
            r = [1]
        if (r == [0]):
            messagebox.showwarning('The Result','No TenYearCHD')
            print(r)
        elif (r == [1]):
            messagebox.showinfo('The Result','Yes TenYearCHD')
            print(r)
    except ValueError:
        messagebox.showerror('Attention','There is no data or the data is not in digits form\nPlease, Enter your dat
```

These is the def that have the model of ML for prediction process.

model = pickle.load(open('LR_model.sav', 'rb'))

First, we load the saved model file with pickle library, then we check that each input contain digits with try except. After that we pass all inputs to the model to predict and lower the threshold to get more accuracy result.

profFrame Class for Profile windows

```
class profFrame(Toplevel):
    def __init__(self, prof):
        #Constructor
        self.prof_frame = prof
        Toplevel.__init__(self)
        self.geometry("500x500")
        self.configure(bg='deepskyblue')
        self.title('Profile')
        self.iconbitmap(r'heart-512.ico')

        #fonts
        helv10 = tkFont.Font(family='Helvetica', size=12, weight='bold')

        #load profile image
        URL = mainFrame.profImg
        u = urlopen(URL)
        raw_data = u.read()
        u.close()
        im = Image.open(BytesIO(raw_data))
        resized = im.resize((160, 180),Image.ANTIALIAS)
        photo = ImageTk.PhotoImage(resized)
```

We did the same thing with others child frames.

Build a new class with new constructor that inherited from MyApp and initializes new settings.

In Profile frame we used urllib to load the profile link in the app and display it with PIL and configure its dimensions and resize the image.

Logout process and others windows transitions

```
#logout from account
def logout(self):
    self.destroy()
    self.main_frame.show()
    self.main_frame.clearinfo()
    hand = open("rem.txt","r+")
    hand.truncate(0)
    hand.close()
    messagebox.showinfo('Successfull','Logged Out')

#While exsiting this window
def Exit(self):
    self.destroy()
    self.main_frame.quit()

def profile(self):
        subFrame = profFrame(self)

def helps(self):
        subFrame = helpFrame(self)

def about(self):
        subFrame = aboutFrame(self)

def predhist(self):
        subFrame = predhistFrame(self)

def hist(self):
        subFrame = histFrame(self)
```

Here is where the user can log out by logout def that destroy every opened session, windows and process then return to the MyApp class to display the parent windows of the app.

When the user log out the remember me data is being cleard.

Ex: subFrame = predhistFrame(self)

This line that open specific window. Like this one for profile window.

The def profile can be run by a command in a button or menubar command as shown in the next page.

Some design code for menu bar, labels, entries, buttons and checkbox and their properties.

Some lines of Design Code using Tkinter

```
#UI
#menubar
menubar = Menu(self)

settingsMenu = Menu(menubar, tearoff=0)
settingsMenu.add_command(label="Profile", command=self.profile)
settingsMenu.add_command(label="Logout", command=self.logout)
settingsMenu.add_command(label="Save")
settingsMenu.add_separator()
settingsMenu.add_command(label="Help", command=self.helps)
settingsMenu.add_command(label="About", command=self.about)
settingsMenu.add_separator()
settingsMenu.add_command(label="Exit", command=self.Exit)
menubar.add_cascade(label="Settings", menu=settingsMenu)
```

```
#Design
ageLabel = Label(self, text = "Age", width = 10, fg='white', bg='deepskyblue')
ageLabel['font'] = helv10
ageLabel.grid(row = 3, column = 3)
global ageEntry
ageEntry = Entry(self, width = 20)
ageEntry.grid(row = 3, column = 4)
```

```
global rem
rem = IntVar()
remCheck = Checkbutton(self.frame, text = "Remmeber me", variable = rem, onvalue = 1, offvalue = 0, height=5
                       , width = 20, bg='deepskyblue',activebackground='deepskyblue')
remCheck['font'] = tkFont.Font(size=10)
remCheck.grid(row = 8, column = 6)

forgetLabel = Label(self.frame, text="forget your password?", fg="blue", cursor="hand2", bg='deepskyblue')
forgetLabel['font'] = tkFont.Font(size=10, underline=1)
forgetLabel.grid(row = 8, column = 7)
forgetLabel.bind("<Button-1>", lambda e: self.forgetPass())

logbtn = Button(self.frame, text="Log in", command=lambda:[self.login(), self.remmeber()],
                cursor="hand2", bd= 1, fg='deepskyblue', bg='white')
logbtn['font'] = helv12
logbtn.grid(row = 10, column = 6,columnspan=2)
```
